# DJ‐1 depletion prevents immunoaging in T‐cell compartments

**DOI:** 10.15252/embr.202153302

**Published:** 2022-01-17

**Authors:** Ni Zeng, Christophe M Capelle, Alexandre Baron, Takumi Kobayashi, Severine Cire, Vera Tslaf, Cathy Leonard, Djalil Coowar, Haruhiko Koseki, Astrid M Westendorf, Jan Buer, Dirk Brenner, Rejko Krüger, Rudi Balling, Markus Ollert, Feng Q Hefeng

**Affiliations:** ^1^ Department of Infection and Immunity Luxembourg Institute of Health (LIH) Esch‐sur‐Alzette Luxembourg; ^2^ Faculty of Science, Technology and Medicine University of Luxembourg Esch‐sur‐Alzette Luxembourg; ^3^ Transversal Translational Medicine Luxembourg Institute of Health (LIH) Strassen Luxembourg; ^4^ Luxembourg Centre for Systems Biomedicine (LCSB) University of Luxembourg Belvaux Luxembourg; ^5^ Laboratory for Developmental Genetics RIKEN Center for Integrative Medical Sciences Yokohama Japan; ^6^ AMED‐CREST Japanese Agency for Medical Research and Development Yokohama Japan; ^7^ Institute of Medical Microbiology University Hospital Essen University Duisburg‐Essen Essen Germany; ^8^ Centre Hospitalier de Luxembourg (CHL) Luxembourg Luxembourg; ^9^ Department of Dermatology and Allergy Center Odense Research Center for Anaphylaxis (ORCA) University of Southern Denmark Odense Denmark; ^10^ Present address: Institute of Molecular Psychiatry University of Bonn Bonn Germany

**Keywords:** aging, CD8 T cells, immune aging, immunosenescence, PARK7/*DJ‐1*, Immunology, Molecular Biology of Disease

## Abstract

Decline in immune function during aging increases susceptibility to different aging‐related diseases. However, the underlying molecular mechanisms, especially the genetic factors contributing to imbalance of naïve/memory T‐cell subpopulations, still remain largely elusive. Here, we show that loss of DJ‐1 encoded by *PARK7*/DJ‐1, causing early‐onset familial Parkinson’s disease (PD), unexpectedly diminished signs of immunoaging in T‐cell compartments of both human and mice. Compared with two gender‐matched unaffected siblings of similar ages, the index PD patient with DJ‐1 deficiency showed a decline in many critical immunoaging features, including almost doubled non‐senescent T cells. The observation was further consolidated by the results in 45‐week‐old DJ‐1 knockout mice. Our data demonstrated that DJ‐1 regulates several immunoaging features via hematopoietic‐intrinsic and naïve‐CD8‐intrinsic mechanisms. Mechanistically, DJ‐1 depletion reduced oxidative phosphorylation (OXPHOS) and impaired TCR sensitivity in naïve CD8 T cells at a young age, accumulatively leading to a reduced aging process in T‐cell compartments in older mice. Our finding suggests an unrecognized critical role of DJ‐1 in regulating immunoaging, discovering a potent target to interfere with immunoaging‐ and aging‐associated diseases.

## Graphical abstract

Deficiency in a key familial Parkinson’s disease gene, PARK7/DJ‐1, reduces immunoaging in T cell compartments of both human and mice at both cellular phenotypic and functional levels.

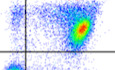

## Introduction

Decline in immune function during aging, known as immunoaging (Gruver *et al*, 2007; Nikolich‐Žugich, 2018), increases susceptibility to different complex non‐communicable (Niccoli & Partridge, 2012) and infectious diseases (CDC, 2019; Akbar & Gilroy, 2020), therefore creating a primary healthcare burden with the increasing elderly populations worldwide (Aw *et al*, 2007; National Institute on Aging, National Institutes of Health, & World Health Organization (WHO), 2011). A growing body of evidence has linked the chronic infection, especially cytomegalovirus (CMV) (Nikolich‐Zugich, 2008; Brunner *et al*, 2011; Pawelec & Derhovanessian, 2011; Fulop *et al*, 2013), to the cause of immunoaging in the organismal level. A number of pathways (Cavanagh *et al*, 2011; Mannick *et al*, 2014; Lanna *et al*, 2017; Pereira *et al*, 2020) have been associated with immunoaging. However, the genetic factors contributing to the regulation of the ratio alteration among different T‐cell subpopulations during aging as well as the regulation of other immunoaging features still remain largely elusive (Sansoni *et al*, 2014; Goronzy & Weyand, 2019).

## Results and Discussion

**Figure 1:**
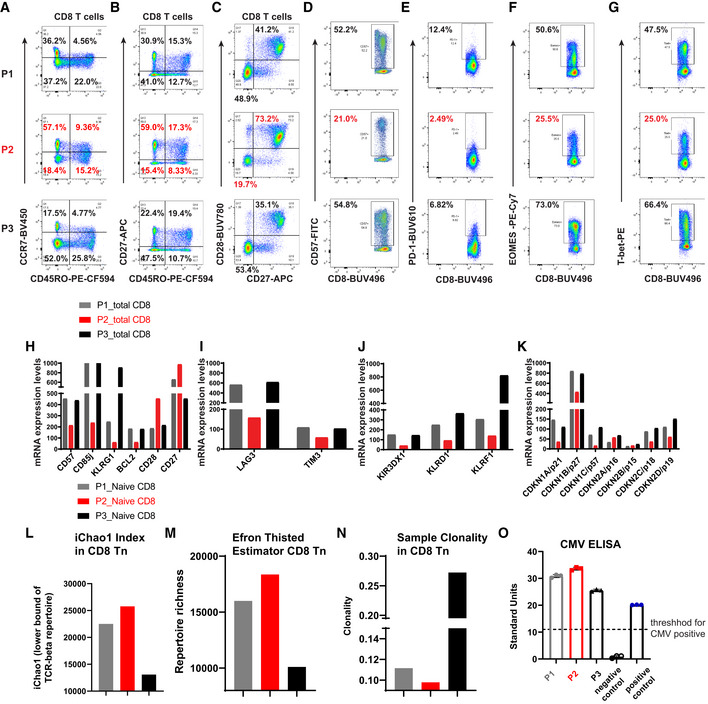
DJ‐1 deficiency diminishes immunoaging in CD8 T cells of an index patient A–CExpression of CD45RO and CCR7 (A), CD45RO and CD27 (B), and CD27 and CD28 (C) on peripheral blood CD8 T cells of three participants [P1 (heterozygous mutation), P2 (homozygous mutation), and P3 (heterozygous) for the c.192G>C mutation in the *DJ‐1* gene].D–GExpression of CD57 (D), PD‐1 (E), EOMES (F), and T‐bet (G) on peripheral blood CD8 T cells of three participants. The enlarged number in the corresponding gate represents the corresponding percentage out of the parent population.H–KmRNA expression of senescence‐related genes (H), exhaustion‐related genes (I), KIR or KLR genes (J), and cyclin‐dependent kinase inhibitor genes (K) in sorted total CD8 T cells of the peripheral blood from three participants.L, MComparison of the lower bound of TCR‐beta repertoire (L) and richness (M) of naïve CD8 T cells among three participants.NThe sample clonality index of TCR repertoire of naïve CD8 T cells among three participants.OSerology detection of CMV infection for three participants. The threshold of 11 was decided according to the manufacture instruction. The data in panel O represent as mean of technical replicates ± standard deviation (SD). Data information: the legends P1_Naive CD8, P2_Naive CD8, and P3_Naive CD8 refer to the panels (L–N). The mRNA transcriptomic analysis was performed in total CD8 T cells.

**Figure EV1:**
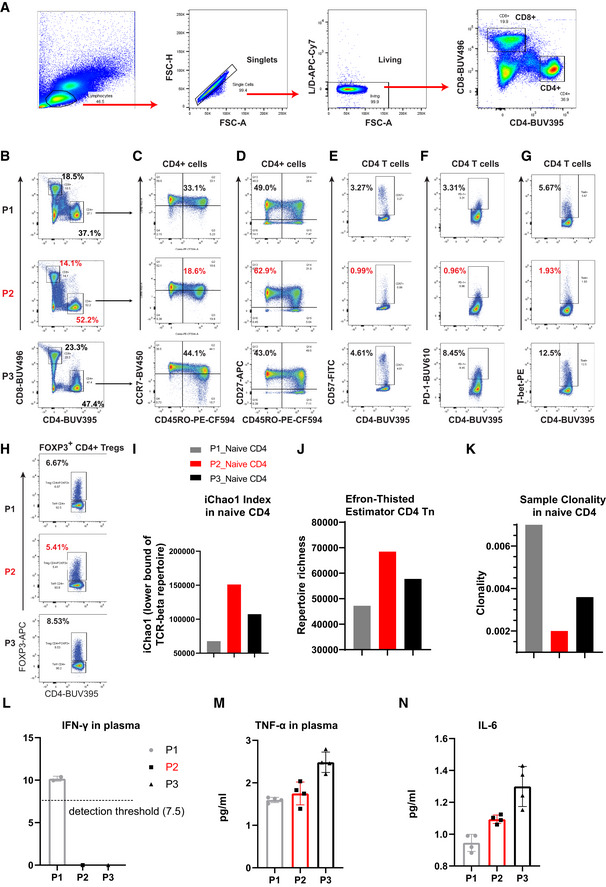
The DJ‐1‐devoid index patient showed diminished immunoaging features also in CD4 T cells AGating strategy to define CD4 and CD8 T cells in human peripheral blood mononuclear cells (PBMC).BPercentages of total CD4 and CD8 T cells among the living lymphocyte singlets of peripheral blood of three participates [P1 (heterozygous mutation), P2 (homozygous mutation), and P3 (heterozygous)].C, DCoexpression of CCR7 and CD45RO (C), CD27 and CD45RO (D) on peripheral blood CD4 T cells of three participants.E–GExpression of CD57 (E), PD‐1 (F), and T‐bet (G) on peripheral blood CD4 T cells of three participants.HFrequency of FOXP3^+^CD4^+^ Tregs among total CD4 T cells in the peripheral blood from three participants.I, JComparison of the lower bound of TCR‐beta repertoire (I) and richness (J) of sorted naïve CD4 T cells of three participants.KThe sample clonality index of TCR repertoire of naïve CD4 T cells of three participants.L–NCytokine measurement of IFN‐γ (L), TNF‐α (M), and IL‐6 (N) in the plasma of three participants. The dots/symbols in cytokine results represent technical replicates. The other five tested cytokines were either undetectable or below fit curve. For cytokine measurement, data are mean ± SD.

Our recent work has demonstrated that DJ‐1 depletion reduced CD4 regulatory T cells (Treg) cellularity only in aged, but not young adult mice (preprint: Danileviciute *et al*, 2019). Since Treg frequency increases during natural aging (Raynor *et al*, 2012), we hypothesized that DJ‐1 might regulate immunoaging in other T‐cell compartments. To leverage the translational potential of this work, we started our analysis from an index Parkinson’s disease (PD) patient carrying the homozygous c.192G>C mutation in the DJ‐1 gene and two of his siblings, who are unaffected heterozygous carriers of the same PD causing mutation (Burbulla *et al*, 2017). In comparison with the unaffected age‐matched siblings (all with the same sex, 56–63‐year old at the time of sampling), the frequency of circulating naïve (CD45RO^−^CCR7^+^CD27^+^) CD8 T cells was approximately doubled, but the frequency of terminally‐differentiated effector (CD45RO^−^CCR7^−^CD27^−^) and effector memory (CD45RO^+^CCR7^−^) CD8 T cells (Fig 1A and B) was much lower in the patient without DJ‐1 expression. Consistent with enhanced proportion of naïve T cells, the proband relative to the two siblings almost doubled the frequency of non‐senescent CD8 T cells, e.g., CD27^+^CD28^+^ CD8 T cells (Fig 1C). The frequency of senescent or exhausted T cells in the index patient, such as CD57^+^, PD‐1^+^, Eomes^+^, and Tbet^+^ CD8 T cells, was reduced to almost half of the two siblings (Fig 1D–G). A similar effect was observed for CD4 T cells (Fig EV1A–G). Consistent with the Treg frequency change in aged whole‐body DJ‐1 KO mice (preprint: Danileviciute *et al*, 2019), the frequency of FOXP3^+^CD4^+^ Tregs also declined in the affected patient versus the two unaffected siblings (Fig EV1H).

To more comprehensively characterize the expression of other senescence‐related genes, we performed genome‐scale transcriptomic analysis in sorted CD8 T cells of the three siblings using microarray. Notably, many immunosenescence marker genes (Goronzy & Weyand, 2013), such as killer immunoglobulin‐like receptors (Braun *et al,* 2020), killer cells lectin‐like receptors (KLRs), and exhaustion markers, e.g., KLRG1, CD85j, LAG3, TIM3, and several cyclin‐dependent kinase inhibitors including p21, known to increase during aging, have all been decreased substantially in the patient with a homozygous DJ‐1 mutation relative to the heterozygous carriers (Fig 1H–K). During aging, the TCR repertoire diversity decreases in naïve T cells (Ahmed *et al*, 2009; Qi *et al*, 2014; Egorov *et al*, 2018). In line with other observations, the TCR repertoire diversity, which is positively correlated with iChao1 index (Fig 1L) and richness (Fig 1M), but negatively correlated with sample clonality index (Fig 1N), was increased in sorted naïve CD8 T cells (CD8 Tn) of the index patient. This also held true for the TCR repertoire diversity in naïve CD4 T cells (CD4 Tn, Fig EV1I–K). It is well accepted that some chronic infections, especially CMV, markedly accelerate immunoaging (Brunner *et al*, 2011). We therefore applied serological testing for CMV IgG antibody titers and found that all the three subjects were CMV positive (Fig 1O). Furthermore, there was no clinical evidence for a specific susceptibility to chronic diseases or repetitive infections of all the three siblings. No increase or decrease trend was observed in systemic levels of relevant pro‐inflammatory cytokines (Ferrucci & Fabbri, 2018) in the index patient versus the two unaffected siblings (e.g., low levels of plasma IFN‐γ, TNF‐α, IL‐6, and undetectable or below fit curve range for IL‐1b, IL‐4, IL‐5, IL‐17a, and IL‐10 among all the three siblings) (Fig EV1L–N). Thus, these data encouraged us to believe that the reduced immunoaging observed in the index patient was driven by DJ‐1 deficiency but not simply due to CMV infection or other chronic infectious diseases or systematic inflammation from either siblings.

**Figure 2:**
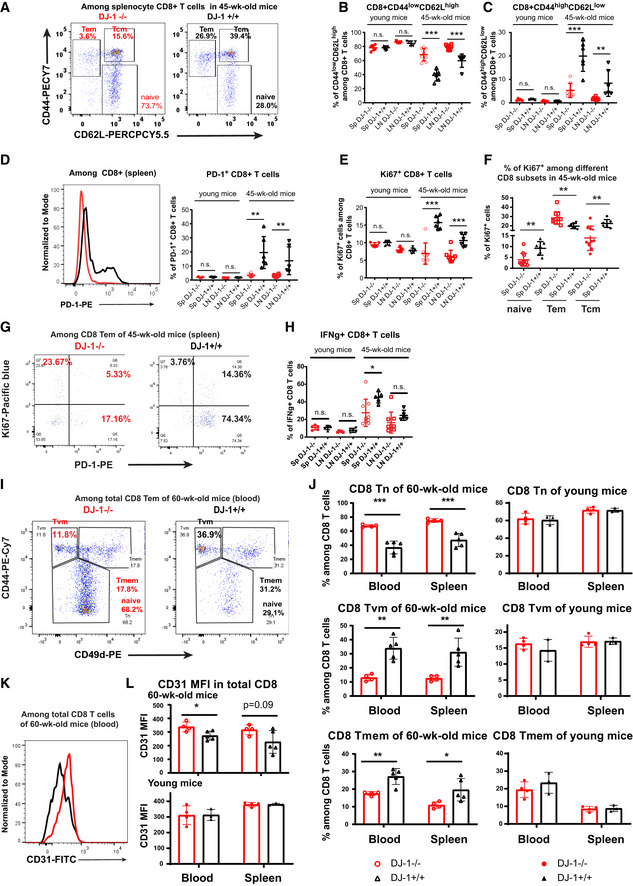
*Dj‐1* depletion reduces signs of immunoaging in murine CD8 T cells ARepresentative flow‐cytometry plots of CD44 and CD62L expression on total CD8 T cells of 45‐week‐old *Dj‐1* KO and age‐ and sex‐matched WT mice.B, CPercentages of CD44^low^ CD62L^high^ (Tn) (B) and CD44^high^ CD62L^low^ (Tem) (C) cells among total CD8 T cells of spleen and pLNs from young and 45‐week‐old *Dj‐1* KO and WT littermates (young KO, *n* = 5; young WT, *n* = 5; 45‐week‐old KO, *n* = 8; 45‐week‐old WT, *n* = 6; for 45‐week‐old mice, data pooled from 2 independent experiments).DRepresentative histogram overlay of PD‐1 expression among total CD8 T cells in spleen of 45‐week‐old mice (left panel) and percentages of PD‐1^+^ cells among total CD8 T cells (right panel).EPercentages of Ki‐67^+^ cells among total CD8 T cells.FPercentage of Ki‐67^+^ cells among splenic CD8 Tn, Tem, and Tcm in 45‐week‐old *Dj‐1* KO and age‐ and sex‐matched WT mice.GRepresentative flow‐cytometry plots of Ki‐67 and PD‐1 among splenic CD8 Tem of 45‐week‐old mice.HIFN‐γ production in CD8 T cells of spleen and pLNs after *in vitro* stimulation using 50 ng/ml of PMA and 750 ng/ml of ionomycin for 5 h.IRepresentative flow‐cytometry plots of CD44 and CD49d among blood CD8 T cells of 60‐week‐old mice.JPercentages of Tn (top), Tvm (middle), and Tmem (bottom) among 60‐week‐old or young mice (young KO, *n* = 4; young WT, *n* = 3; 60‐week‐old KO, *n* = 4; and 60‐week‐old WT, *n* = 5).KRepresentative histogram of CD31 among blood CD8 T cells of 60‐week‐old mice.LCD31 MFI of total CD8 T cells (young KO, *n* = 4; young WT, *n* = 3; 60‐week‐old KO, *n* = 4; and 60‐week‐old WT, *n* = 5). Data information: SP and LN represent spleen and lymph nodes, respectively. Results represent at least four (B‐H) or two (I‐L) independent experiments. Data are mean of biological replicates ± SD. Each dot/symbol represents the measurement from one mouse. The *P*‐values are determined by a two‐tailed non‐paired Student’s *t*‐test. n.s. or unlabeled, not significant, *P ≤ 0.05, **P ≤ 0.01, and ***P ≤ 0.001. Source data are available online for this figure.

**Figure EV2:**
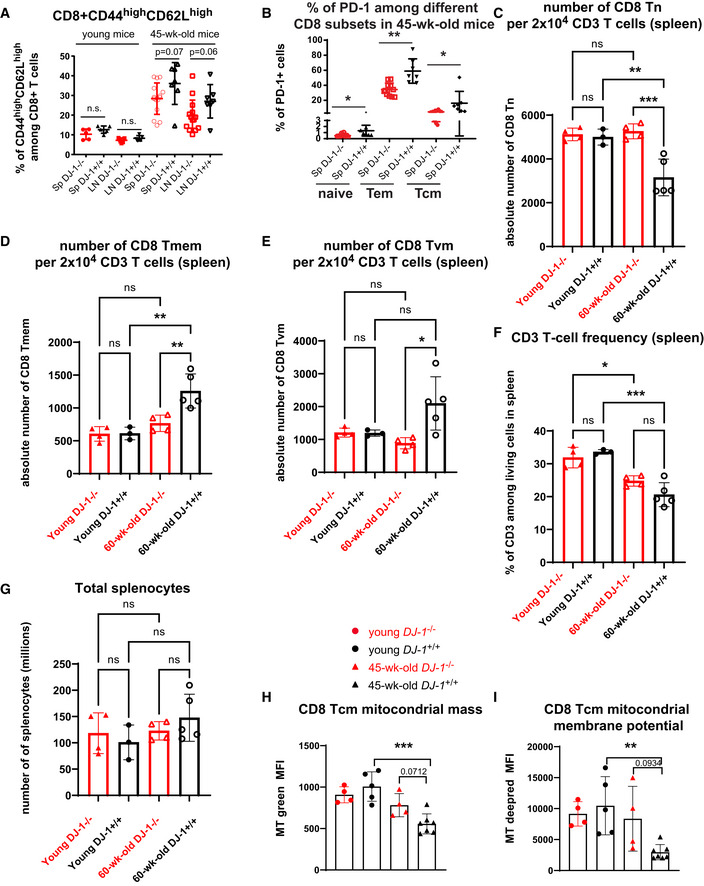
Extended characterization of diminished signs of immunoaging in CD8 T‐cell compartments APercentages of CD44^high^ CD62L^high^ cells (Tcm) among total CD8 T cells of spleen and pLNs from young and 45‐week‐old *Dj‐1* KO and WT littermates (young KO, *n* = 5; young WT, *n* = 5; 45‐week‐old KO, *n* = 15; 45‐week‐old WT, *n* = 7; for 45‐week‐old mice, data pooled from 3 independent experiments).BPercentages of PD‐1^+^ cells among splenic CD8 Tn, Tem, and Tcm subsets in the 45‐week‐old *Dj‐1* KO and age‐ and sex‐matched WT mice (45‐week‐old KO, *n* = 11 and 45‐week‐old WT, *n* = 7).C–EAbsolute number of CD8 Tn (C), Tmem (D), and Tvm (E) within the acquired 20K of living CD3 T cells from spleen of young or 60‐week‐old mice (young KO, *n* = 4; young WT, *n* = 3; 60‐week‐old KO, *n* = 4; and 60‐week‐old WT, *n* = 5).FFrequency of CD3 T cells among living singlet lymphocytes from spleen of young or 60‐week‐old mice (young KO, *n* = 4; young WT, *n* = 3; 60‐week‐old KO, *n* = 4; and 60‐week‐old WT, *n* = 5).GThe total number of splenocytes of young or 60‐week‐old mice (young KO, *n* = 4; young WT, *n* = 3; 60‐week‐old KO, *n* = 4; and 60‐week‐old WT, *n* = 5).H, IComparison of CD8 Tcm mitochondrial mass (H) and membrane potential (I) of young and 45‐week‐old *Dj‐1* KO and WT mice (young KO, *n* = 4; young WT, *n* = 5; 45‐week‐old KO, *n* = 4; and 45‐week‐old WT, *n* = 7). MT, MitoTracker. Data information: Results represent at least four (A, B), two (C–G), and three (H, I) independent experiments. Data are mean ± SD. The *P*‐values are determined by a two‐tailed non‐paired Student’s *t*‐test (A, B, H, and I). Ordinary one‐way ANOVA with Sidak’s multiple comparison test was applied in C‐G. n.s. or unlabeled, not significant, *P ≤ 0.05, **P ≤ 0.01, and ***P ≤ 0.001.

*DJ‐1* loss‐of‐function mutations are a rare cause of monogenic PD (Pankratz *et al*, 2006) and we thus were unable to identify further patients with *PARK7*‐related PD available for biosampling in our extended networks (Boussaad *et al*, 2020). Therefore, to obtain more statistical power and mechanistic insights, we analyzed whole‐body *Dj‐1* knockout (KO, for simplicity “whole‐body” will be left out afterwards unless different lines were used) mice. Here, we examined relevant features of immunoaging in *Dj‐1* KO mice, which were developed elsewhere to study the impact of *Dj‐1* on neurodegenerative diseases, i.e., PD (Pham *et al*, 2010). As a hallmark of natural aging, memory T cells increase while their counterpart, Tn, decrease (Nikolich‐Zugich, 2008). According to others’ observations, only mice older than 18 months are considered as aged mice, while 10–14‐month‐old mice are considered as middle‐aged ones (Flurkey *et al*, 2007). However, we detected a significantly higher frequency of CD8 naïve T cells (Tn, CD44^low^CD62L^high^) accompanied by a lower frequency of effector memory (CD44^high^CD62L^low^, Tem) already in 45‐week‐old (~45 weeks old, i.e., ~10‐month‐old unless otherwise specified) *Dj‐1* KO mice versus age‐ and sex‐matched WT (Fig 2A–C). A tendency for reduced frequency of central memory CD8 T cells (CD44^high^CD62L^high^, CD8 Tcm) was also observed in 45‐week‐old *Dj‐1* KO mice (*P* = 0.07, Fig EV2A). No change in the memory/naïve compartments of CD8 T cells was observed in young adult (8–15‐week‐old, simplified as “young” later on) *Dj‐1* KO mice (Figs 2A–C and EV2A). Although the concept of immunosenescence does not fully overlap with that of exhaustion (Akbar & Henson, 2011; Xu & Larbi, 2017), a considerable intersection exists between these two age‐related immunophenotypes and their functional consequences. In contrast to the increased PD‐1 expression in T cells during natural aging (Lee *et al*, 2016), we observed a significantly lower expression of the key exhaustion marker PD‐1 among homeostatic total CD8 T cells (Fig 2D) and different CD8 subsets (Fig EV2B) in 45‐week‐old, but not young *Dj‐1* KO versus matched WT mice. This observation indicates that the exhaustion process might slow down in 45‐week‐old *Dj‐1* KO mice. In addition to exhaustion, PD‐1 is also regarded as one of the key activation markers of T cells (Ahn *et al*, 2018). Therefore, to determine whether our observation on reduced activation or cell exhaustion is related to altered homeostatic proliferation, we also assessed proliferation markers, e.g., Ki‐67, in different T cell subsets under homeostatic conditions. Although the frequency of Ki‐67^+^ cells among total CD8, Tn, and Tcm was significantly decreased (Fig 2E and F), the homeostatic proliferation of CD8 Tem was significantly augmented (Fig 2F). Furthermore, the expression of PD‐1 and Ki‐67 showed a largely mutually exclusive pattern among CD8 Tem (Fig 2G). These results indicate that reduced PD‐1 expression in 45‐week‐old *Dj‐1* KO mice was not simply attributable to a general reduction in proliferation, at least not in CD8 Tem under homeostatic conditions. Relevant cytokines, e.g., IFN‐γ, increase among CD8 T cells during natural aging (Bandres *et al*, 2000). Therefore, we also analyzed IFN‐γ production in CD8 T cells. Conforming to other diminished immunoaging phenotypes, IFN‐γ was decreased in CD8 T cells of 45‐week‐old *Dj‐1* KO versus WT mice following *in vitro* PMA/ionomycin stimulation (Fig 2H). Consistent with the observations in the index PD patient with *DJ‐1* deficiency, our results show that *Dj‐1* KO mice display reduced immunoaging phenotypes in CD8 T‐cell compartments.

During natural aging, virtual memory (VM) T cells (antigen‐inexperienced memory T cells) accumulate (Chiu *et al*, 2013; Quinn *et al*, 2018). Therefore, we also examined the proportion of CD8 Tvm (CD44^high^CD49d^low^) in *Dj‐1* KO mice. In line with the reduced immunoaging notion, we also observed decreased frequency of both CD8 Tvm and Tmem (CD44^high^CD49d^high^) among total CD8 T cells of 60‐week‐old *Dj‐1* KO versus WT mice (Fig 2I and J). After excluding those antigen‐inexperienced Tvm cells, the frequency of CD8 naïve T cells (CD44^low^) was still higher in 60‐week‐old *Dj‐1* KO versus WT mice (Fig 2I and J), further confirming the reduced immunoaging phenotype in *Dj‐1* KO mice. As expected, the frequency of CD8 Tvm was lower in young WT adult mice compared with 60‐week‐old WT mice. Nevertheless, the frequency of CD8 Tvm did not show any difference between young *Dj‐1* KO and WT mice (Fig 2J), again demonstrating an aging‐related phenotype in *Dj‐1* KO mice. In line with the natural aging process, the absolute number of CD8 Tn was decreased while that of CD8 Tmem was increased within the same amount of total living CD3 T cells already in 60‐week‐old versus young WT mice (Fig EV2C). Notably, the absolute number of CD8 Tn was kept quite constant between 60‐week‐old and young *Dj‐1* KO mice and was higher in 60‐week‐old *Dj‐1* KO relative to WT mice (Fig EV2C). The higher amount of CD8 Tn was accompanied with a lower amount of CD8 Tmem in 60‐week‐old *Dj‐1* KO mice (Fig EV2D). Encouragingly, the absolute number of CD8 Tvm was still lower in 60‐week‐old *Dj‐1* KO versus WT mice, although that did not significantly change in 60‐week‐old mice compared with young mice (Fig EV2E). As reported by others (Pieren *et al*, 2019), we also observed a decline of splenic CD3 frequency in 60‐week‐old versus young mice no matter which genotype groups they belonged to (Fig EV2F). In consideration of the non‐significant change in both CD3 frequency and total splenocytes between 60‐week‐old *Dj‐1* KO and WT mice (Fig EV2G), the estimated absolute numbers of CD8 subsets in total splenocytes should retain a similar pattern as the absolute numbers of CD8 subsets within the same amount of total CD3 T cells. In summary, both the frequency and absolute number of CD8 subsets showed a relatively reduced aging phenotype in *Dj‐1* KO versus WT mice.

**Figure EV3:**
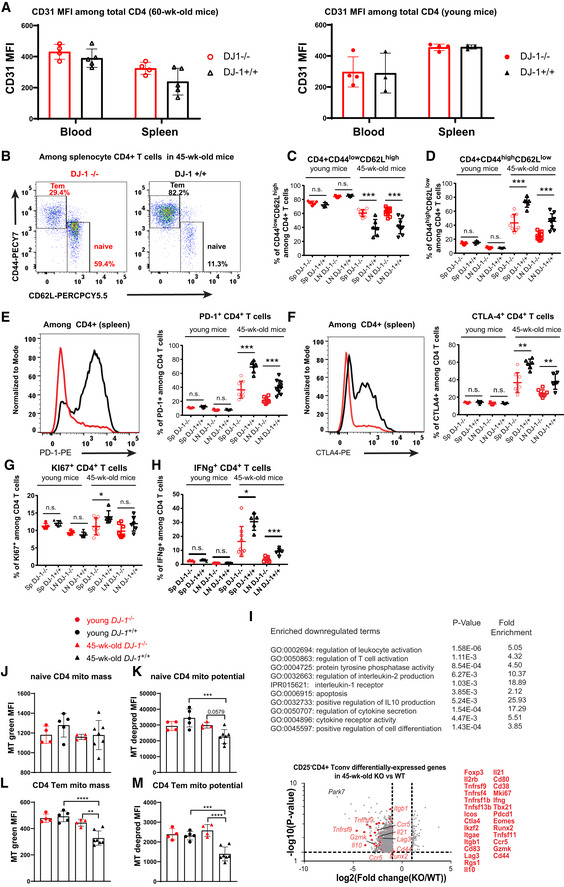
*Dj‐1* depletion also reduced signs of immunoaging in CD4 T‐cell compartments AExpression level of CD31 among total blood or splenocyte CD4 T cells of 45‐week‐old (left) and young (right) mice.BRepresentative flow‐cytometry plots of CD44 and CD62L expression on total CD4 T cells of 45‐week‐old *Dj‐1* KO and age‐ and sex‐matched WT mice (young KO, *n* = 5; young WT, *n* = 5; 45‐week‐old KO, *n* = 8; 45‐week‐old WT, *n* = 6; for 45‐week‐old mice, data pooled from 2 independent experiments; of note, more than one pLNs might be taken from several mice).C, DPercentages of CD44^low^ CD62L^high^ (Tn) (C) and CD44^high^ CD62L^low^ (Tem) (D) cells among total CD4 T cells of spleen and pLNs from young and 45‐week‐old *Dj‐1* KO and WT littermates.ERepresentative histogram overlay of PD‐1 expression among total CD4 T cells in spleen of 45‐week‐old mice (left panel) and percentages of PD‐1^+^ cells among total CD4 T cells (right panel).FRepresentative histogram overlay of CTLA‐4 expression among total CD4 T cells in spleen of 45‐week‐old mice (left panel) and percentages of CTLA‐4^+^ cells among total CD4 T cells (right panel).GPercentages of Ki‐67^+^ cells among total CD4 T cells.HIFN‐γ production in CD4 T cells of spleen and pLNs after *in vitro* stimulation using 50 ng/ml of PMA and 750 ng/ml of ionomycin for 5 h.IThe selected significantly enriched GO‐terms and pathways among the downregulated genes in CD4 Tconv cells from 45‐week‐old *Dj‐1* KO mice versus the age‐ and gender‐matched WT littermates from microarray analysis (upper panel). Lower panel, volcano plot shows both downregulated and upregulated differentially expressed genes in splenic CD4 T cells from three 45‐week‐old *Dj‐1* KO mice versus three age‐matched WT littermates. The dashed line in y axis corresponds to the value of 1.3 (*P* = 0.05), while the two dashed lines in *x*‐axis correspond to −1 and 1 (change fold = 2). A two‐tailed Student *t*‐test was used to calculate the *P* values (for detailed microarray analysis method, refer to Materials and Methods).J, KComparison of naive CD4 (Tn) mitochondrial mass (mito mass, J) and membrane potential (mito potential, K) of young and 45‐week‐old *Dj‐1* KO and WT mice.L, MComparison of CD4 Tem mitochondrial mass (mito mass, L) and membrane potential (mito potential, M) of young and 45‐week‐old *Dj‐1* KO and WT mice. SP and LN represent spleen and lymph nodes, respectively. Data information: results represent at least four (B–G) and three (J–M) independent experiments. Data are mean of biological replicates ± SD. Each biological replicate indicates the measurement from one individual mouse. The *P*‐values are determined by a two‐tailed un‐paired Student’s *t*‐test. n.s. or unlabeled, not significant, *P ≤ 0.05, **P ≤ 0.01, ***P ≤ 0.001, and ****P ≤ 0.0001.

CD31 is a marker for recently emigrated thymic CD4 T cells and is mainly expressed in naïve T cells (Kimmig *et al*, 2002; Junge *et al*, 2007; Tanaskovic *et al*, 2010). To check whether the enhanced frequency of naïve T cells in 45‐week‐old *Dj‐1* KO mice is due to a higher thymic output, we analyzed the expression of CD31 among peripheral T cells. The expression of CD31 among CD4 T cells in both spleen and blood did not show a significant difference between 60‐week‐old *Dj‐1* KO versus WT mice (Fig EV3A), indicating that the observed enhanced frequency of naïve T cells was not simply attributable to a higher thymic output. We also observed no difference in CD31 expression among CD4 T cells from young *Dj‐1* KO versus WT mice (Fig EV3A). In CD8 T cells, CD31 is mainly expressed in naïve and central memory cells (Newman *et al*, 2018). Therefore, we also analyzed CD31 expression among CD8 T cells. In line with the enhanced frequency of CD8 Tn, interestingly, we also observed significantly higher levels of CD31 expression among CD8 T cells (Fig 2K and L), especially in blood of 60‐week‐old *Dj‐1* KO versus WT mice. In young mice, we again observed no difference in CD31 expression among CD8 T cells between *Dj‐1* KO and WT mice (Fig 2L). In short, the observed higher frequency of naïve T cells was not simply due to an enhanced thymic outcome in older *Dj‐1* KO versus WT mice.

Although CD8 T cells are more susceptible to aging‐related changes (Czesnikiewicz‐Guzik *et al*, 2008), we have also observed diminished immunoaging features in CD4 T cells of the index patient. Therefore, we also analyzed the murine CD4 T‐cell compartments. As expected, similar effects were observed in CD4 T cells of 45‐week‐old *Dj‐1* KO mice (Fig EV3B–H). To gain a more comprehensive picture, we performed transcriptomic analysis of CD4^+^CD25^−^ conventional T cells (Tconv) sorted from 45‐week‐old mice under homeostatic conditions. In accordance with the enhanced proportions of naïve T cells, our pathway enrichment analysis showed that several pathways involved in T‐cell receptor signaling pathway and positive regulation of cell differentiation were significantly affected among the downregulated genes in Tconv of 45‐week‐old *Dj‐1* KO versus WT mice (Fig EV3I). Notably, many memory T‐cell or aging‐related markers (Mogilenko *et al*, 2021) and/or memory T‐cell development‐related genes were also among the downregulated genes (Fig EV3I). These results show that DJ‐1 depletion also diminished immunoaging signs in CD4 T‐cell compartments.

**Figure EV4:**
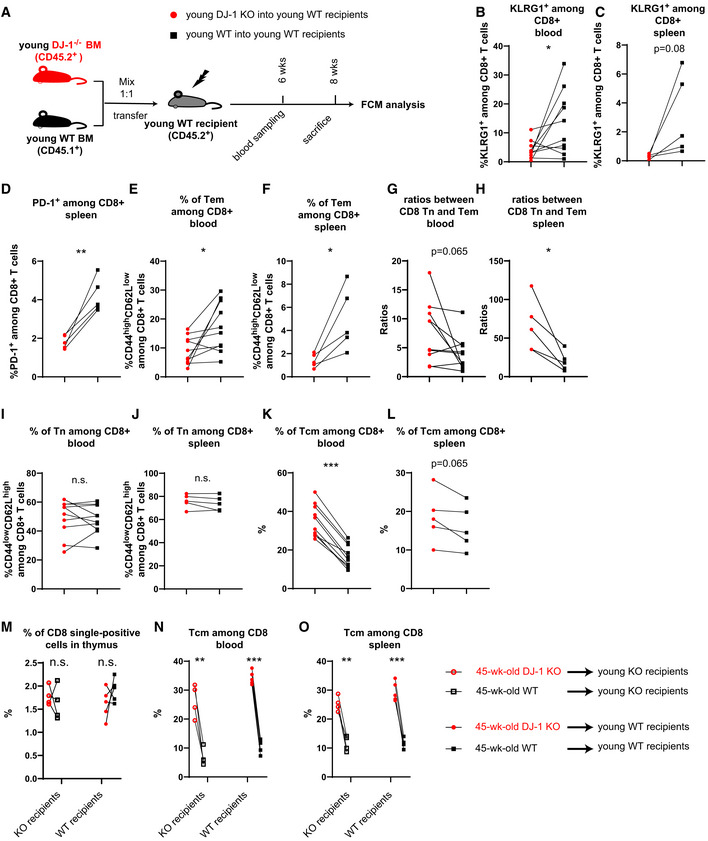
*Dj‐1* ablation regulated KLRG1 and PD‐1 expression as well as the ratios between CD8 Tn and Tem in a hematopoietic‐intrinsic manner but the accumulation of CD8 Tn and Tcm in a complicated manner ASchematic of the experimental setup of bone marrow transplantation. A total of 10E6 of bone marrow cells from young *Dj‐1* KO mice (CD45.2^+^) and WT mice (CD45.1^+^) (1:1 mix) were transferred into lethally‐irradiated young WT recipients (CD45.2^+^) by i.v. injection. Mice stably engrafted with donor cells were sacrificed for flow cytometry (FCM) analysis later.B, CPercentages of KLRG1^+^ CD8 T cells derived from young *Dj‐1* KO and WT donor BM cells in blood (B) and spleen (C) within young WT recipients (*n* = 5; blood sampled twice at both 6 and 8 weeks).DPercentages of PD‐1^+^ cells among total CD8 T cells derived from young *Dj‐1* KO and WT BM cells in spleen within young WT recipients.E, FPercentages of CD8 Tem derived from young *Dj‐1* KO and WT BM cells in blood (E) and spleen (F) within young WT recipients.G, HRatios between CD8 Tn and Tem cells developed from CD45.1 (WT) or CD45.2 (KO) BM cells in blood (G) and spleen (H) within young WT recipients.I, JPercentages of CD8 Tn in blood (I) and spleen (J) derived from young *Dj‐1* KO and WT BM cells within young WT recipients.K, LPercentage of CD8 Tcm among total CD8 T cells derived from young *Dj‐1* KO and WT BM cells in blood (K) and spleen (L) of young WT recipients.MPercentages of CD8 single‐positive cells among thymus originated from 45‐week‐old *Dj‐1* KO and WT BM cells within young *Dj‐1* KO or WT recipients following reconstitution.N, OPercentages of CD8 CD44^high^CD62L^high^ (Tcm) cells in blood (N) and spleen (O) derived from 45‐week‐old *Dj‐1* KO and WT BM cells within young *Dj‐1* KO or WT recipients. Data information: Results represent two independent experiments. The *P*‐values are determined by a two‐tailed paired Student’s *t*‐test. n.s., not significant, *P ≤ 0.05, **P ≤ 0.01, and ***P ≤ 0.001.

Since DJ‐1 is a multi‐functional protein ubiquitously expressed in different types of tissues and cells (Wilson, 2011) and immunoaging involves many types of immune and non‐immune cells (Nikolich‐Žugich, 2018), we next asked whether the reduced immunoaging in 45‐week‐old *Dj‐1* KO mice is the result of a hematopoietic‐intrinsic or non‐hematopoietic regulation. To this end, we generated mixed bone marrow (BM) chimeras by transferring BM cells mixed from young CD45.1 *Dj‐1* WT and CD45.2 *Dj‐1* KO donor mice into lethally‐irradiated young WT recipients (Fig EV4A). In this experiment, both *Dj‐1* KO and WT CD8 T cells developed under the same host WT environmental conditions. If the difference observed between that of KO and WT origins is consistent with the alteration in 45‐week‐old *Dj‐1* KO versus WT mice, it is attributable to hematopoietic‐intrinsic regulations. As demonstrated by others (Cho *et al*, 2000), the development of CD8 Tem from CD8 Tn is radically accelerated under lymphopenia (e.g., *Rag1* deficient mice). Following lethal irradiation in recipients, a similar accelerated development of CD8 Tem might also take place in our model (Fig EV4A). Because of this reason, we expect at least a certain degree of aging phenotypes in the BM chimera model, although the cells were derived from young donors. Interestingly, we indeed observed some hematopoietic‐intrinsic phenotypes for the *Dj‐1*‐mediated immunoaging role. For instance, the expression of the critical immunoaging marker KLRG1 among blood CD8 T cells of *Dj‐1*‐KO origin was already significantly lower than that of *Dj‐1*‐WT‐derived counterparts (Fig EV4B) and also showed a trend to lessen in spleen (*P* = 0.08, Fig EV4C). This clearer phenotype in blood is in accordance with the notion that Tem are mainly distributed in non‐lymphoid tissues (Masopust *et al*, 2001) and KLRG1 is mainly expressed among CD8 Tem. The exhaustion marker PD‐1, which increases during aging, also showed a similar change as did KLRG1 (Fig EV4D). The frequency of CD8 Tem was significantly lower in *Dj‐1*‐KO‐derived cells (Fig EV4E and F). In accordance with the data in 45‐week‐old *Dj‐1* KO mice, the ratios between CD8 Tn and Tem were already higher in the *Dj‐1*‐KO‐derived cells than that from WT‐origin cells (Fig EV4G and H). These data indicate that *Dj‐1* exhibits a hematopoietic‐intrinsic role for regulating not only the expression of several immunoaging‐related markers, but also the proportion of CD8 Tem and the ratio between CD8 Tn and Tem.

However, the percentages of CD8 Tn did not show any significant difference between lymphocytes developed from young CD45.2 and CD45.1 BM cells in both spleen and blood (Fig EV4I and J). Moreover, the percentages of CD8 Tcm derived from *Dj‐1* KO BM cells were higher than that from *Dj‐1‐*WT origin within young WT recipients (Fig EV4K and L). Considering the observation in 45‐week‐old *Dj‐1* KO mice (Fig EV2A), our results from young‐donor‐BM transplantation indicate that *Dj‐1* deficiency mediates accumulation of CD8 Tn and Tcm via either a hematopoietic‐extrinsic or an aging‐dependent manner or in combination of both. These mixed/divided phenotypes from the young‐donor‐BM chimeras urged us to investigate the potential effects of aging and non‐hematopoietic aspects on the *Dj‐1*‐mediated immunoaging.

**Figure 3:**
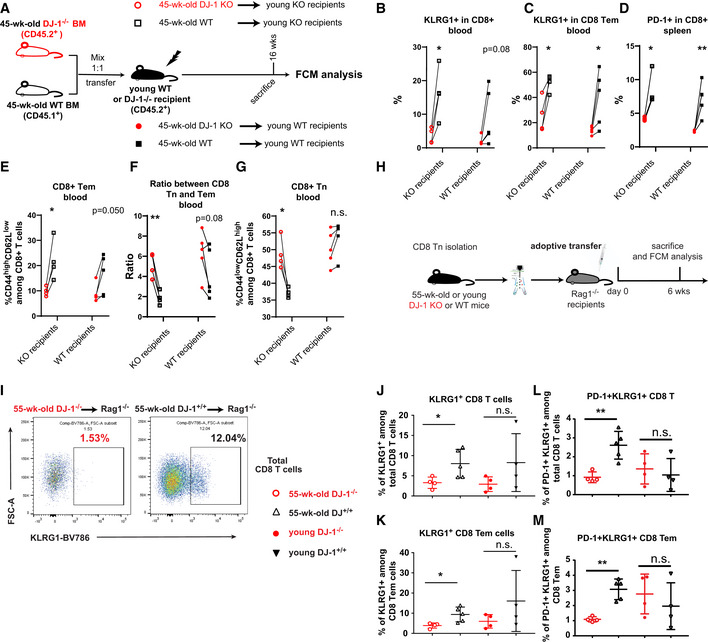
*Dj‐1* depletion inhibits the expression of key immunoaging markers KLRG1 and PD‐1 in a hematopoietic‐ and CD8‐Tn‐intrinsic manner Schematic of the experimental setup of BM transplantation. A total of 10E6 of bone marrow cells from 45‐week‐old *Dj‐1* KO mice (CD45.2^+^) and WT mice (CD45.1^+^) (1:1 mix) were transferred into lethally irradiated young *Dj‐1* KO or WT recipients (CD45.2^+^) by i.v. injection. Mice stably engrafted with donor cells were sacrificed for flow cytometry (FCM) analysis later.Percentages of KLRG1^+^ CD8 T cells derived from 45‐week‐old *Dj‐1* KO and WT BM cells within young *Dj‐1* KO (*n* = 4) or WT recipients (*n* = 5).Percentages of KLRG1^+^ among CD8 Tem derived from 45‐week‐old *Dj‐1* KO and WT BM cells within young *Dj‐1* KO or WT recipients.Percentages of PD‐1^+^ population among CD8 T cells derived from 45‐week‐old *Dj‐1* KO and WT BM cells within young *Dj‐1* KO (*n* = 4) or WT recipients (*n* = 5).Percentages of CD8^+^ CD44^high^CD62L^low^ (Tem) cells derived from 45‐week‐old *Dj‐1* KO and WT BM cells within young *Dj‐1* KO or WT recipients.Ratios between blood CD8 Tn and Tem cells developed from two types of BM cells within young *Dj‐1* KO or WT recipients.Percentages of CD8^+^ CD44^low^CD62L^high^ (Tn) cells derived from 45‐week‐old *Dj‐1* KO and WT BM cells within young *Dj‐1* KO or WT recipients.Schematic of the experimental setup of CD8 Tn adoptive transfer. 2.5E5 naïve CD8 T cells isolated from young or 55‐week‐old *Dj‐1* KO and WT littermates were injected into Rag‐1 deficient mice by i.v. injection. 6 weeks later, mice were sacrificed for FCM (flow cytometry) analysis.Representative flow‐cytometry plots of KLRG1^+^ population among total CD8 T following adoptive transfer of CD8 Tn from 55‐week‐old mice.Percentages of KLRG1^+^ population among total CD8 T cells (55‐week‐old KO, *n* = 4; 55‐week‐old WT, *n* = 5; young KO, *n* = 4; and young WT, *n* = 4).Percentages of KLRG1^+^ population among CD8 Tem cells.Percentages of KLRG1^+^PD‐1^+^ population among total CD8 T cells (55‐week‐old KO, *n* = 4; 55‐week‐old WT, *n* = 5; young KO, *n* = 4; and young WT, *n* = 4).Percentages of KLRG1^+^PD‐1^+^ population among CD8 Tem cells. Data information: Results from BM transfer and adoptive transfer of CD8 Tn represent two independent experiments. Data are mean ± SD. The *P*‐values are determined by a two‐tailed paired (B–G) or non‐paired (J–M) Student’s *t*‐test. n.s. or unlabeled, not significant, *P ≤ 0.05 and **P ≤ 0.01.

To study the effects aforementioned, we generated mixed BM chimeras by transferring 45‐week‐old CD45.1 *Dj‐1* WT BM cells and CD45.2 *Dj‐1* KO BM cells into irradiated young WT or KO recipients (Fig 3A). Due to the ethic limitation, we were unable to use aged or even middle‐aged mice as recipients. Similar to that in young‐donor‐BM chimeras (Fig EV4B–F), the percentages of cells expressing the immunoaging‐related markers, e.g., KLRG1 (Fig 3B and C) and PD‐1 (Fig 3D) as well as the frequency of CD8 Tem (Fig 3E) were lower among CD8 cells reconstituted from 45‐week‐old *Dj‐1* KO versus WT BM donors, independent from the types of recipients, again supporting a hematopoietic‐intrinsic mechanism. The ratios between CD8 Tn and Tem developed from 45‐week‐old *Dj‐1* KO versus WT BM donors were significantly higher in young KO recipients or with a tendency to increase also in young WT recipients (*P* = 0.08, Fig 3F). These consistent data from both young‐ and 45‐week‐old‐donor‐BM chimeras together suggests that *Dj‐1* exhibits an aging‐BM‐independent, but hematopoietic‐intrinsic role for regulating the expression of KLRG1 and PD‐1 among CD8 T cells, the frequency of CD8 Tem as well as the ratio between CD8 Tn and Tem.

Interestingly, the mixed BM chimeras did not show a significant difference in the frequency of CD8 Tn developed from 45‐week‐old *Dj‐1* KO versus WT BM donors as long as the recipients were WT mice, no matter from young (Fig EV4I and J) or 45‐week‐old donors (Fig 3G). Notably, consistent with the observation in 45‐week‐old *Dj‐1* KO versus WT mice, following reconstitution within young *Dj‐1*‐KO, but not ‐WT recipients, the proportion of blood CD8 Tn developed from 45‐week‐old *Dj‐1* KO versus WT BM cells was already significantly higher (Fig 3G). At the same time, no significant change was observed in the frequency of CD8 single‐positive cells in thymus among 45‐week‐old *Dj‐1*‐KO versus ‐WT origins, within either type of young *Dj‐1* KO or WT recipients (Fig EV4M), ruling out an abnormal thymic development of matured CD8 T cells. These results suggest that the enhanced frequency of CD8 Tn in 45‐week‐old *Dj‐1* KO versus WT mice requires the involvement of *Dj‐1*‐deficient non‐hematopoietic cells. Opposed to the observations in 45‐week‐old *Dj‐1* KO versus WT mice, the percentages of CD8 Tcm of 45‐week‐old *Dj‐1*‐KO versus *Dj‐1*‐WT origin were higher within both types of young recipients (Fig EV4N and O). The results about CD8 Tcm percentages were consistent between young‐ and 45‐week‐old‐BM models, regardless of within WT or KO recipients. These together essentially suggest the involvement of *Dj‐1*‐deficient aging microenvironmental factors in reduced accumulation of CD8 Tcm within 45‐week‐old *Dj‐1* KO mice. In short, our data firmly demonstrate that *Dj‐1* depletion enhances the ratio between CD8 Tn and Tem and inhibits the expression of immunoaging‐related markers in a hematopoietic‐intrinsic but aging‐BM‐independent way, while regulating the CD8 Tn and Tcm accumulation in a more complicated manner.

**Figure EV5:**
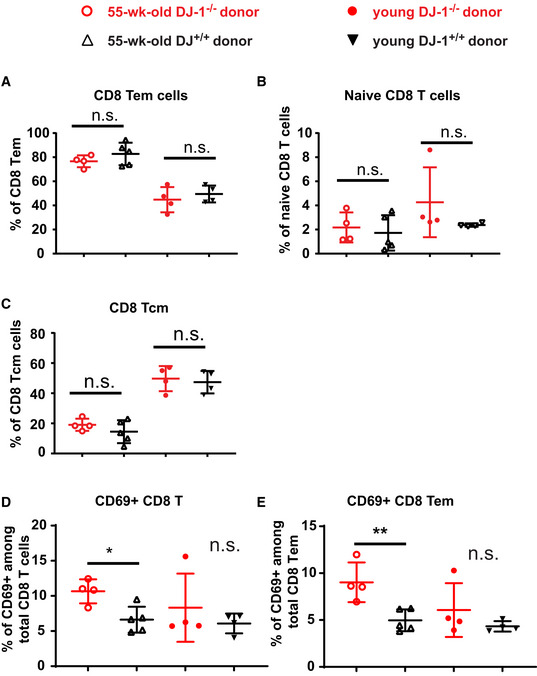
Extended characterization of CD8 T‐cell compartments following adoptive transfer of CD8 Tn into *Rag1*‐deficient mice A–CPercentages of splenic CD8 Tem (CD44^high^CD62L^low^) (A), Tn (CD44^low^CD62L^high^) (B), and Tcm (CD44^high^CD62L^high^) (C) cells (55‐week‐old KO, *n* = 4; 55‐week‐old WT, *n* = 5; young KO, *n* = 4; and young WT, *n* = 4).D, EPercentage of CD69^+^ cells among total CD8 T cells (D) and CD8 Tem cells (E) in adoptive transfer experiment. Data information: Each symbol represents one mouse. Results represent two independent experiments. Data are mean ± SD. The *P*‐values are determined by a two‐tailed non‐paired Student’s *t*‐test. n.s. or unlabeled, not significant, *P ≤ 0.05 and **P ≤ 0.01.

The immunoaging process in T‐cell compartments involves many types of cells (Nikolich‐Žugich, 2018) and BM‐derived cells include far more than T cells. Previous data have shown that the homeostatic proliferation of CD8 Tn is enhanced during natural aging (Rudd *et al*, 2011) and the homeostatic proliferation of CD8 Tn drives the differentiation into CD8 Tem in Rag1^–/–^ lymphopenic mice (Cho *et al*, 2000). In 45‐week‐old *Dj‐1* KO versus WT mice, the homeostatic proliferation of CD8 Tn was reduced (Fig 2F). Therefore, we hypothesized that this reduced homeostatic proliferation of CD8 Tn might impede or dysregulate the development into Tem cells and consequently the aging process of T cells. To test whether *Dj‐1* depletion has a T‐cell‐intrinsic effect on the transition from CD8 Tn into Tem subsets, we performed adoptive transfer of CD8 Tn sorted from young or 55‐week‐old *Dj‐1* KO or WT mice into young *Rag1^–/–^* recipients, where the development of CD8 Tem is radically accelerated under lymphopenia (Cho *et al*, 2000) (Fig 3H). Remarkably, the essential murine immunoaging marker KLRG1 was significantly lower in total CD8 T cells developed from 55‐week‐old *Dj‐1* KO versus WT donor cells (Fig 3I and J). This phenomenon in total CD8 T cells was mainly due to the fact that from 55‐week‐old donor cells, CD8 T cells mainly consisted of CD8 Tem in this model (Fig EV5A–C), where KLRG1 was significantly reduced in KO versus WT‐originated cells (Fig 3K). Furthermore, the frequency of the double‐positive cells expressing both KLRG1 and PD‐1 among total CD8 T cells and CD8 Tem was significantly lower in 55‐week‐old *Dj‐1* KO CD8‐Tn‐originated cells (Fig 3L and M). In line with the reduced expression of key immunoaging markers, the expression of the T‐cell activation marker CD69 was significantly increased in total CD8 T cells and CD8 Tem derived from CD8 Tn of 55‐week‐old *Dj‐1*‐KO versus ‐WT origins (Fig EV5D and E). Nevertheless, the frequency of CD8 Tn, Tem, and Tcm in the *Rag1*‐null recipients was not different in both comparisons of CD8 T cells from young *Dj‐1* KO versus ‐WT origins as well as that from 55‐week‐old *Dj‐1*‐KO versus ‐WT origins (Fig EV5A–C), indicating the existence of CD8‐Tn‐extrinsic factors and/or requiring aging‐microenvironmental factors. Nevertheless, the observed CD8‐Tn‐intrinsic role for *Dj‐1* in regulating key immunoaging‐related hallmarks in CD8 T cells, at least already partially, contribute to the diminished immunoaging phenotype in middle‐aged *Dj‐1* KO mice.

**Figure 4:**
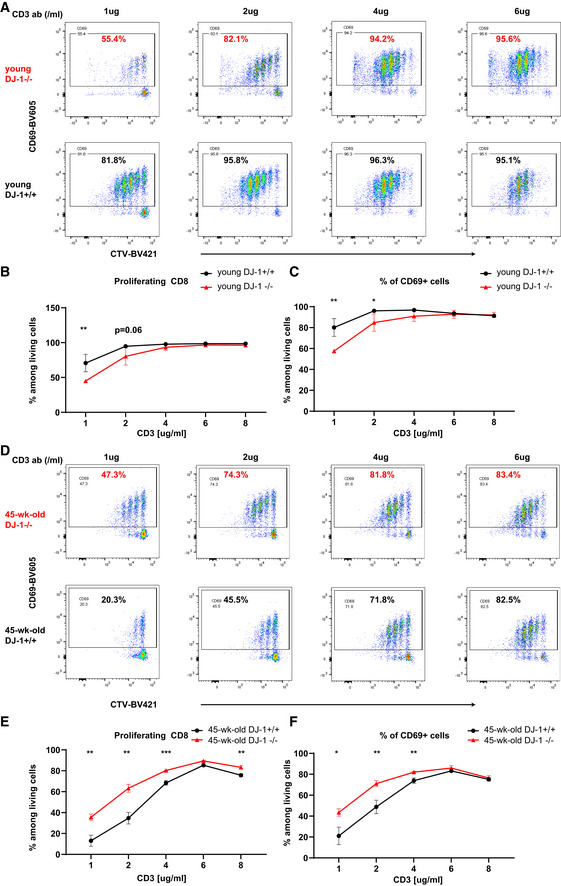
*Dj‐1* depletion compromises TCR sensitivity of CD8 Tn in young mice but relatively enhances TCR sensitivity of CD8 Tn in 45‐week‐old mice in a dose‐dependent manner Representative flow cytometry data of CD69 and Celltrace violet (CTV) staining on gated living CD8 T cells. The purified CD8 Tn were isolated from young mice and stimulated by different doses of anti‐CD3 ab for 72 h. Enlarged number representing the percentage of the corresponding gate.The percentages of proliferating cells among living CD8 singlets (CD8 Tn were from young mice).The percentages of CD69^+^ cells among living CD8 T singlets (CD8 Tn were from young mice).Representative flow cytometry data of CD69 and CTV staining on gated living CD8 T cells. The purified CD8 Tn were isolated from 45‐week‐old mice and stimulated by different doses of anti‐CD3 ab for 72 h. Enlarged number representing the percentage of the corresponding gate.The percentages of proliferating cells among living CD8 singlets (CD8 Tn were from 45‐week‐old mice).The percentages of CD69^+^ cells among living CD8 T singlets (CD8 Tn were from 45‐week‐old mice). Data information: Results represent at least two independent experiments (A‐F). Data are mean ± SD. All the CD8 Tn cells were first pooled from 3 to 4 mice of the same group before exposing to different doses of anti‐CD3 ab. The error bar (SD) here essentially refers to technical replicates. The *P*‐values are determined by a two‐tailed non‐paired (B‐C, E‐F) Student’s *t*‐test. Unlabeled, not significant, *P ≤ 0.05, **P ≤ 0.01, and ***P ≤ 0.001.

We next asked whether a “younger” CD8 cellular phenotype is also reflected at the functional levels from older *Dj‐1* KO relative to age‐ and gender‐matched WT mice. To this end, we analyzed TCR sensitivity of isolated CD8 Tn using different doses of anti‐CD3 antibodies (ab). Interestingly, *Dj‐1* KO versus WT CD8 Tn isolated from young mice showed reduced TCR sensitivity at the concentration of 1 and 2 µg/ml of plate‐coated anti‐CD3 ab (Fig 4A–C). A dose‐dependent effect indicates that the sensitivity of *Dj‐1* KO CD8 Tn to TCR stimulation was compromised, but not completely lost. In contrast to the observation in young mice, in line with the reduced immunoaging notion, we indeed observed a relatively higher proliferation and activation of *Dj‐1* KO versus WT CD8 T cells from 45‐week‐old mice at concentrations of 1 and 2 µg/ml of anti‐CD3 ab (Fig 4D–F). The difference, although still significant, gradually disappeared while further increasing the anti‐CD3 ab concentration (Fig 4D–F). The relatively enhanced TCR sensitivity of CD8 Tn isolated from 45‐week‐old *Dj‐1* KO versus WT mice could be well explained by the fact that the TCR sensitivity of *Dj‐1* KO CD8 Tn was quite preserved, while the TCR sensitivity in WT mice was dramatically decreased during aging. Moreover, as shown here, the TCR sensitivity difference increased in an aging‐dependent manner, again indicating that *Dj‐1* regulates immunoaging at the functional levels. It has been well documented that multiple rounds of stimulation (Spaulding *et al*, 1999) or extensive homeostatic proliferation (Minato *et al*, 2020), as implicated during the natural aging process, might eventually cause T cell senescence or aging phenotypes. The compromised TCR sensitivity of *Dj‐1* KO CD8 Tn generated at a younger age indicates that a reduced activation could accumulate fewer overall homeostatic replications during the long‐lasting natural aging process. After reaching a certain age, those long‐lived CD8 Tn (Vrisekoop *et al*, 2008) with fewer accumulated replication rounds would still have a higher proliferation potential when stimulated.

**Figure 5:**
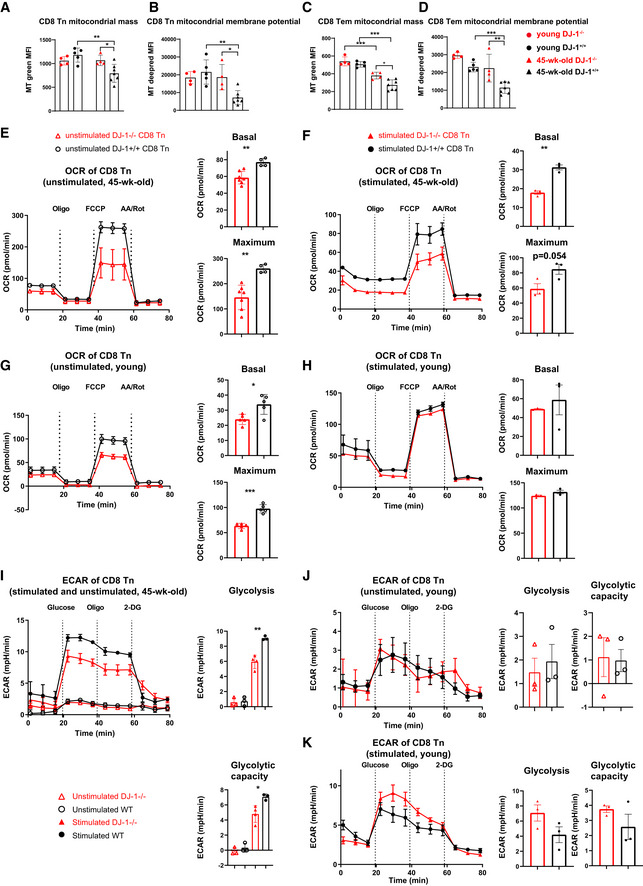
*Dj‐1* depletion impairs OXPHOS essentially in resting CD8 Tn A, BComparison of splenic CD8 Tn mitochondrial mass [Mitotracker (MT) green MF, A] and membrane potential (MT Deep Red, B) of young and 45‐week‐old *Dj‐1* KO and WT mice.C, DComparison of CD8 Tem mitochondrial mass (C) and membrane potential (D) of young and 45‐week‐old *Dj‐1* KO and WT mice.E–HOxygen consumption rate (OCR) of unstimulated (E) and stimulated CD8 Tn (F) by 2 µg/ml of anti‐CD3 and 1 µg/ml of anti‐CD28 for 24 h. The CD8 Tn were isolated from 45‐week‐old mice. OCR of unstimulated (G) and stimulated CD8 Tn (H) by anti‐CD3 and –CD28 for 24 h. The CD8 Tn were isolated from young mice (G and H). Left of each OCR measurement panel, representation plots following Mito stress test. Right of each panel, quantitation of basal and maximum respiration under Mito stress test conditions The dashed line indicates the time when the corresponding modulator oligomycin (Oligo), carbonyl cyanide‐4 (trifluoromethoxy) phenylhydrazone (FCCP) or rotenone and antimycin (AA/Rot) was added.I–KExtracellular acidification rate (ECAR) of unstimulated and stimulated CD8 Tn by anti‐CD3 and ‐CD28 antibodies for 24 h (I). The CD8 Tn were isolated from 45‐week‐old mice. ECAR of unstimulated (J) and stimulated (K) CD8 Tn by anti‐CD3 and ‐CD28 for 24 h. The CD8 Tn were isolated from young mice (J and K). Left of each ECAR measurement panel, representation plots following Glycolysis stress test. Right of each panel, quantitation of glycolysis and glycolytic capacity under glycolysis stress test conditions. The dashed line indicates the time when the corresponding modulator glucose, oligomycin (Oligo), or 2‐deoxy‐glucose (2‐DG) was added. Data information: Of note, except for the results within the same subfigure (e.g., J), the values are not necessarily fully comparable due to the measurements at different days and not‐fully‐identical cell numbers between unstimulated and stimulated conditions from young and 45‐week‐old mice. Results represent two independent experiments. Data are mean ± SD. All the CD8 Tn cells were first pooled from 4 to 5 mice of the same group before being stimulated or not. The error bar (SD) here essentially refers to technical replicates. The *P*‐values are determined by a two‐tailed Student’s *t*‐test. ns or unlabeled, not significant, *P ≤ 0.05, **P ≤ 0.01, and ***P ≤ 0.001.

We next wondered what could be the potential mechanisms causing the enhanced frequency of CD8 Tn and decreased frequency of Tem, i.e., the reduced immunoaging process in 45‐week‐old *Dj‐1* KO mice. T‐cell‐conditional deletion of the mitochondrial transcription factor A (*Tfam*), suppressing mitochondria DNA content, accelerates inflammaging (Desdin‐Mico *et al*, 2020) and aging impairs mitochondrial homeostasis (Picca *et al*, 2018). Therefore, we first checked whether our observation in cellular phenotypes has anything to do with mitochondrial fitness. To this end, we measured mitochondrial mass and membrane potential in different T cell subsets. Similar to the observation in skeletal muscle (Crane *et al*, 2009), both mitochondrial mass and membrane potential of different CD8 T‐cell subsets (CD8 Tn, Tem, and Tcm) were declined in 45‐week‐old WT versus young WT mice (Figs 5A–D and EV2H and I). Notably, in both CD8 Tn and Tem from 45‐week‐old mice, the mitochondrial mass and membrane potential were significantly enhanced in *Dj‐1* KO versus WT mice (Fig 5A–D). Still similar to other CD8 subsets, CD8 Tcm mitochondria mass (*P* = 0.07) and membrane potential (*P* = 0.09) showed a tendency to increase in 45‐week‐old *Dj‐1* KO versus WT mice (Fig EV2H and I). Consistent with other cellular data in young mice, significant difference was observed in neither mitochondrial mass nor membrane potential of different CD8 T‐cell subsets between young *Dj‐1* KO and WT mice (Figs 5A–D and EV2H and I). Thus, the concurring phenotypes between enhanced mitochondrial mass and functions in the CD8 subsets, especially in Tn and Tem, are in agreement with reduced immunoaging at cellular levels of 45‐week‐old *Dj‐1* KO versus WT mice in consideration of the observations in T cells devoid of *Tfam* (Desdin‐Mico *et al*, 2020). We also observed a similar effect on mitochondrial mass and membrane potential in CD4 T cells of *Dj‐1* KO mice (Fig EV3J–M). The role of aging in mitochondrial fitness of T cells is still a matter of controversy. For instance, Quinn *et al* have observed increased mitochondrial footprints/mass in T cells of 45‐week‐old relative to younger mice (Quinn *et al*, 2020), although not in the context of *Dj‐1* KO mice. On the other hand, in line with what we observed, Henson *et al* have demonstrated a reduced mitochondrial mass in the more differentiated CD8 T cell subset [i.e., CD8 terminally‐differentiated effector cells (TEMRA)] relative to other subsets (Henson *et al*, 2014), indicating a decrease in mitochondrial mass of T cells during the aging process. These controversial reports indicate that the general mitochondrial features might not be sufficient to interpret the T‐cell immunoaging phenotypes we observed here and suggest the need of further investigation.

Metabolic shift is crucial for the formation of memory CD8 T cells from naïve CD8 T cells (van der Windt *et al*, 2012; O'Sullivan & Pearce, 2015) and we observed reduced frequency of CD8 Tem but enhanced frequency of CD8 Tn in 45‐week‐old *Dj‐1* KO mice. As demonstrated so far, the enhanced fraction of CD8 Tn was not due to an enhanced thymic outcome in 45‐week‐old *Dj‐1* KO mice. Therefore, we asked whether the impairment in generating CD8 Tem is related to the potential shift of metabolic pathways in *Dj‐1* KO CD8 Tn. To this end, we first analyzed mitochondrial oxidative phosphorylation (OXPHOS), as reflected by O2 consumption rate (OCR) in unstimulated and stimulated CD8 Tn. Notably, not only the basal OXPHOS but also the maximum OXPHOS respiratory capacity was significantly lower in both unstimulated and stimulated CD8 Tn isolated from 45‐week‐old *Dj‐1* KO versus WT mice (Fig 5E and F). Since mitochondrial respiratory capacity is critical for memory CD8 T cell development (van der Windt *et al*, 2012), the reduced OXPHOS in CD8 Tn might well explain the reduced formation of CD8 Tem. *Dj‐1* KO versus WT CD8 Tn from young mice displayed impaired OXPHOS (both basal and maximum levels) only in resting, but not stimulated naïve CD8 T cells, although anti‐CD3/‐CD28 stimulation has enhanced OXPHOS in general, independent of the genotype groups (Fig 5G and H). Meanwhile, we also could not exclude the possibility that the observed non‐significant effect of *Dj‐1* depletion on OXPHOS in stimulated CD8 Tn might be a simple dose‐dependent response to TCR stimulation. This has already been indicated in our TCR sensitivity experiments (Fig 4A–C), where the effect of 2 µg/ml of anti‐CD3 ab was already much weaker compared with that of dose of 1 µg/ml.

To better understand the potential metabolic shift in *Dj‐1* KO mice, we further analyzed the glycolytic pathway using Seahorse glycolysis stress tests by measuring the extracellular acidification rate (ECAR). The unstimulated quiescent CD8 Tn mainly rely on OXPHOS (van der Windt & Pearce, 2012; Zhang & Romero, 2018). Indeed, the unstimulated CD8 Tn isolated from 45‐week‐old mice of both genotype groups displayed a very low level of glycolysis and glycolytic capacity (Fig 5I). Following stimulation, CD8 Tn shift their metabolism and rely more on the glycolytic pathway (van der Windt & Pearce, 2012). After stimulation by anti‐CD3 and ‐CD28 for 24 h, CD8 Tn of 45‐week‐old mice, no matter from WT or KO mice, exhibited an enhanced level in both glycolysis and glycolytic capacity, compared with resting CD8 Tn (Fig 5I). However, *Dj‐1* KO versus WT CD8 Tn from 45‐week‐old mice showed a reduced glycolysis and glycolytic capacity (Fig 5I). On one hand, the reduction in both OXPHOS and glycolysis in CD8 Tn of 45‐week‐old *Dj‐1* KO mice could explain the reduced formation of CD8 Tem during aging. On the other hand, the substantial impairment in the essential energy‐producing pathways in *Dj‐1* KO versus WT CD8 Tn of 45‐week‐old mice, is counterintuitive to the increased TCR sensitivity in CD8 Tn of 45‐week‐old mice (Fig 4D–F). A possible explanation could be found in the recent report by Quinn *et al* (2020), which showed that metabolic characteristics do not necessarily predict CD8 T‐cell functional level. In short, the enhanced TCR sensitivity of *Dj‐1* KO CD8 Tn that we observed in 45‐week‐old mice correlated well with enhanced mitochondrial mass and membrane potential (Fig 5A and B), rather than impaired metabolic functions.

As expected, both the glycolysis and glycolytic capacity were almost undetectable in resting CD8 Tn of young mice, regardless of the genotype (Fig 5J), and were not clearly impaired following stimulation in KO versus WT cells (Fig 5K). On the other hand, *Dj‐1* KO CD8 Tn isolated from young mice show impaired OXPHOS in a resting state (Fig 5G), despite both mitochondrial mass and membrane potential of CD8 subsets being comparable between young *Dj‐1* KO versus WT mice (Fig 5A–D). Therefore, although there existed a disassociation between TCR sensitivity and OXPHOS/glycolysis in middle‐aged, i.e., 45‐week‐old mice, the difference in relevant metabolic pathways well predicted the degree of response in young mice. In short, the reduced OXPHOS in resting *Dj‐1* KO CD8 Tn and impaired TCR sensitivity already starting at a younger age accumulatively result in more remaining proliferation potential of those long‐lived CD8 Tn, and consequently lead to a less senescent phenotype in T‐cell compartments after reaching a critical age.

Our findings reveal an unexpected causal link between deficiency in a key monogenic PD gene PARK7/DJ‐1 and reduced immunoaging process in the T cell compartments. The data consistency between both human and mice with DJ‐1 deficiency supports a highly potent strategy to interfere with or model immunoaging for various complex diseases and infectious diseases. Our data showed not only a “younger” cellular phenotype but also a higher functional potential remained, e.g., a relatively higher sensitivity to TCR stimulation of CD8 Tn already in 45‐week‐old (middle‐aged) *Dj‐1* KO versus WT mice. During natural aging, TCR sensitivity of CD8 Tn diminishes in WT mice. Different from that in WT mice, the TCR sensitivity of *Dj‐1* KO CD8 Tn was compromised already at a young age and caused a lower overall homeostatic proliferation of CD8 Tn during the long‐lasting aging course. Meanwhile, the TCR sensitivity in *Dj‐1* KO CD8 Tn was declined not as dramatically as in WT mice during aging. Such a type of compromise in TCR sensitivity of DJ‐1 KO CD8 Tn eventually leads to a higher remaining proliferation potential of CD8 Tn in 45‐week‐old *Dj‐1* KO mice relative to age‐ and gender‐matched WT mice. In short, the difference in the CD8 TCR sensitivity, which was already observed between young *Dj‐1* KO and WT mice, increases in an aging‐dependent manner.

The reduced TCR sensitivity of CD8 Tn already starting in young mice was attributable to impaired OXPHOS, especially at resting/steady state. At the same time, in young mice, we did not observe a clear impairment on both OXPHOS and glycolysis activities in stimulated *Dj‐1* KO CD8 T cells, which indicates that following the accomplishment of stimulation and metabolic mode shift, their key metabolic pathways were able to function normally, at least at the anti‐CD3/CD28 ab concentration we tested. The non‐compromised functionality of stimulated cells was also in accordance with enhanced homeostatic proliferation in relatively more active subsets (i.e., CD8 Tem) of 45‐week‐old *Dj‐1* KO versus WT mice as observed here. Our work supports that the compromised OXPHOS of *Dj‐1* KO CD8 Tn plays a critical role in controlling the conversion process of naïve to Tem CD8 T cells. This DJ‐1‐depletion‐mediated metabolic incompetence to properly develop CD8 Tem eventually contributes to more accumulation of CD8 Tn and consequently a relatively “younger” immune phenotype already in middle‐aged *Dj‐1* KO mice. However, the impairment, but not complete loss of certain metabolic activities, e.g., OXPHOS, is not sufficient to trigger changes in memory/naïve T cell portions at a young age under homeostatic conditions. Meanwhile, it has been well established that mild inhibition of mitochondrial respiration extends the lifespan of various species, including mice and primates (Liu *et al*, 2005; Hwang *et al*, 2012; Pontzer *et al*, 2014), although none of those studies has ever touched immune aspects. It is also worthy to note that the better‐preserved functional potential of CD8 Tn in middle‐aged *Dj‐1* KO mice might be already consistent with others’ observations that the frequency of IFN‐γ^+^ cells is increased among CD4 *Dj‐1* KO T cells following PMA/ionomycin stimulation of total splenocytes from young mice (Jung *et al*, 2014). In summary, here we demonstrated that reduced energy‐producing metabolic activities, especially impaired mitochondrial OXPHOS and consequently contracted TCR sensitivity of CD8 Tn already starting from a younger age, contribute to the overall reduced immunoaging process in *Dj‐1* KO T cells. Our work paves the way for building up a regulatory link between immunometabolism and immunoaging in T cell compartments via DJ‐1. Our work also offers a unique animal model with reduced immunoaging phenotypes to allow researchers to explore the potential roles of a relatively juvenile immune system in the immune‐ and aging‐associated diseases. Understanding the detailed molecular mechanisms through which DJ‐1 regulates immunoaging still requires further investigation using cell‐type‐specific conditional *Dj‐1* KO mice.

## Materials and Methods

### Mice

B6.129P2‐Park7^Gt (XE726) Byg^/Mmucd mice were previously described (Pham *et al*, 2010). The *Dj‐1* knockout mice line used in our lab has been generated by crossing the original line with B6N mice at least 10 generations. *Rag1* ^−/−^ mice with C57BL/6 (B6) background were purchased from the Jackson Laboratory and maintained in the specific pathogen‐free (SPF) animal facility of Luxembourg Institute of Health. CD45.1 mice with B6 background were purchased from Charles River Laboratory and maintained in the SPF animal facility of University of Luxembourg. The *Dj‐1* ^−/−^ (KO), *Dj‐1* ^+/−^, and *Dj‐1* ^+/+^ (WT) mice used in our experiments were gender‐ and age‐matched siblings generated from heterozygous *Dj‐1* ^+/−^ breeding pairs or trios. The *Dj‐1* line including both young‐ and older‐mice cohorts was also kept under the SPF conditions. Animal protocols have been approved either by animal welfare structure from LIH or animal experimentation ethics committee from University of Luxembourg, depending on where the experiments were performed. After institutional checking, each of the protocols was officially authorized by Luxembourg Ministry of Agriculture before starting the animal experiments. We determined the sample size without applying a statistical method in advance, by choosing at least five mice (except for at least three mice for the Tvm and CD31 analysis) per genotype group with the same sex at the given age, according to the best practice in the field. In this project, it not relevant to randomize the animal allocation because we studied the natural immunoaging process of *Dj‐1* KO or WT mice. The littermates were kept in the same cages. The people who performed genotyping were different from the major experimental analysis operators. The investigators were blind to the genotyping information before finishing the flow cytometry analysis.

### Flow cytometry analysis for murine cells

The mice were sacrificed by dislocation of the neck. Spleens and peripheral lymph nodes (pLN) were collected and stored in the ice cold flow‐cytometry staining (FCM) buffer [Ca^2+^ and Mg^2+^ free PBS (Lonza, BE17‐516F) with 2% inactivated fetal bovine serum (FBS) and 2 mM EDTA, pH 8.0]. The pLNs here referred to a mixture of cervical, axillary, and inguinal LNs. Spleens and lymph nodes were minced through a 70‐µM cell strainer. Centrifuge the cell suspension at 350 *g* for 5 min at 4°C. Discard the supernatant and add 3 ml of 1× Red blood cell lysis buffer (BD, 555899) per mouse and incubate at room temperature for 7 min. Add 10 ml of FCM buffer to stop the reaction and centrifuge the cells and discard the supernatant. One million of cells per sample from spleen or pLNs were incubated in FCM buffer together with anti‐mouse CD16/CD32 Fc blocker (BD Biosciences, 553141) at 4°C for 15 min. Dead cells were distinguished using LIVE/DEAD™ Fixable Near‐IR Dead Cell Stain Kit (Life Technologies, L34975). The extracellular antibodies were added and incubated at 4°C for 30 min protected from light. To detect intracellular nuclear proteins and transcriptional factors, cells were fixed and permeabilized using FOXP3/Transcription Factor Fixation kit (eBioscience, 00‐5523‐00) according to the manufacturer’s protocol. Samples were incubated with intracellular antibodies diluted in permeabilized buffer for 30 min at 4°C. Cell acquisitions were performed on a BD LSRFortessa™ and data were analyzed using FlowJo software (v10, TreeStar, now part of BD). All the extracellular and intracellular antibodies are summarized in Appendix Table S1. Of note, not necessarily all the abs listed there were analyzed in the same panel.

### Mitotracker staining

Splenocytes were isolated as described above and stained for 30 min at 37°C in complete RPMI medium [RPMI 1640 medium (Thermo Fisher Scientific, 21870084) containing 10% heat‐inactivated FBS, 1 mM sodium pyruvate (Thermo Fisher Scientific, 11360070), 1X non‐essential amino acids (Sigma Aldrich, M7145), 2 mM GlutaMAX™ (Thermo Fisher Scientific, 35050061), 50 µM beta‐mercaptoethanol (Sigma Aldrich, M7522), 10 mM HEPES (Thermo Fisher Scientific, 15630080), 50 U/ml penicillin, and 50 µg/ml streptomycin (Thermo Fisher Scientific, 15070063)] containing anti‐CD4 PE, anti‐CD8 BUV737, anti‐CD25 BUV395, anti‐CD44 PE‐Cy7, anti‐CD62L PerCP‐Cy5.5, 1:2,000 DAPI, 1:100,000 MitoTracker Green FM (Thermo Fisher Scientific, M7514), and 1:10,000 MitoTracker Deep Red (Thermo Fisher Scientific, M22426). The cells were washed in cold FCM buffer and immediately acquired on a BD LSRFortessa™. The antibodies detecting surface markers for different subsets are provided in Appendix Table S1.

### Seahorse Mito and glycolysis stress tests

Naïve CD8 T cells were isolated using the Naive CD8a^+^ T Cell Isolation Kit (Miltenyi Biotec, 130‐096‐543) and stimulated or not for 24 h with 2 μg/ml anti‐CD3 (BD, 553057, plate coated) and 1 μg/ml anti‐CD28 (BD, 553294, not coated). Glycolysis and OXPHOS were measured in stimulated or unstimulated naïve CD8 T cells, using the Seahorse XF Glycolysis Stress Test Kit (Agilent, 103020‐100; for more seahorse‐related materials, refer to Appendix Table S2) or the Seahorse XF Cell Mito Stress Test Kit (Agilent, 103015‐100), respectively, following manufacture’s instruction. The cells (between 300K to 400K depending on the availability of the given batch) were seeded in Cell‐TAK (354240, Corning)‐coated Seahorse Bioanalyzer XFe96 culture plates with Seahorse XF base medium (102353‐100, Agilent Technologies) containing 1 mM pyruvate (S8636, Sigma‐Aldrich), 2 mM glutamine (G8540, Sigma‐Aldrich), and 2.5 mM glucose (G8769, Sigma‐Aldrich). OCR was measured under basal conditions and in response to 1 µM Oligomycin (103015‐100, Agilent Technologies), 1.0 µM FCCP [carbonyl cyanide‐4 (trifluoromethoxy) phenylhydrazone] (103015‐100, Agilent Technologies), and 1 µM Rotenone and Antimycin A (103015‐100, Agilent Technologies) by the Seahorse XF96 analyzer (Agilent). The results were analyzed by Wave 2.6.0 (Agilent Technologies). For the glycolysis stress test, we used the assay medium: Seahorse XF base medium (102353‐100, Agilent Technologies) plus 2 mM l‐glutamine. The modulators we used include glucose (10 mM), oligomycin (1 μM), and 2‐deoxy‐glucose (2‐DG, Sigma, D8375) (100 mM).

### TCR sensitivity experiment

Naïve CD8 T cells were isolated using the Naive CD8a+ T Cell Isolation Kit (Miltenyi Biotec, 130‐096‐543) and stained with CellTrace™ Violet Cell (CTV) Proliferation Kit (Thermo Fisher Scientific, C34557). CTV was resuspended in 20 µl DMSO to obtain a stock solution of 5 mM, before preparing a 5‐µM working solution in PBS. Naïve CD8 T cells were re‐suspended in 500 µl per 1 million of cells and incubated for 7 min in the incubator at 37°C. After incubation, the cells were washed in 12 ml pre‐warmed complete RPMI medium and centrifuged at 350 *g* for 10 min. The cells were then re‐suspended in complete RPMI and 150K cells were seeded per well of a flat‐bottom 96‐well plate, coated with different concentrations of anti‐CD3 antibodies. After 72 h in the incubator at 37°C, the cells were stained with the antibodies in Appendix Table S1 and acquired on a BD LSRFortessa™ and analyzed by Flowjo software.

### Intracellular cytokine quantification

For intracellular cytokine measurement, cells (2E5) from spleen or pLNs were stimulated by 50 ng/ml PMA (Phorbol 12‐myristate 13‐acetate, Sigma‐Aldrich, P8139) and 750 ng/ml ionomycin (Sigma‐Aldrich, I0634) in the presence of Golgiplug (BD Biosciences, 555029) and Golgistop (BD Biosciences, 554724) for 5 h in 96‐well round‐bottom plates. Following cell surface staining, cells were fixed and permeabilized with Cytofix/Cytoperm buffer (BD Biosciences, 554714). The cytokine antibodies diluted in permeabilized buffer were added and incubated at 4°C for 30 min protected from light. Cells were acquired on a BD LSRFortessa™ and analyzed by Flowjo v10.

### Bone marrow (BM) transplantation

BM cells pooled from the femurs and tibias of *Dj‐1* ^−/−^ mice and age‐, gender‐matched CD45.1 B6 mice were isolated and 1:1 mixed in 100 µl of cold PBS solution (Ca^2+^ and Mg^2+^ free; Lonza, BE17‐516F). Gender‐matched *Dj‐1* KO or WT mice aged 8–12 weeks as recipients were lethally irradiated from a gamma source (RS2000 X‐Ray Biological Irradiator from Rad Source Technologies, two doses of 450 rads with 3 h resting period between the two doses) and 6 h later received 10E6 mixed donor BM cells by intravenous injection. For BM chimeras generated from young donor mice, blood was sampled at 6 and 8 weeks post transplantation. Spleen was analyzed at 8 weeks after transplantation. For BM chimeras generated from 45‐week‐old donor mice, both blood and spleen were analyzed at 4 months post transplantation. Cells were acquired on a BD LSRFortessa™ followed by the analysis with Flowjo v10.

### Adoptive transfer of naïve CD8^+^ T cells

CD90.2^+^ cells from spleen and pLNs of *Dj‐1* ^−/−^ mice and *Dj‐1* ^+/+^ littermates aged 8–12 weeks or ~55 weeks were magnetically isolated and first enriched by using anti‐mouse CD90.2 microbeads (Miltenyi Biotec, 130‐049‐101, also refer to Appendix Table S2). Naïve CD8^+^ T cells (CD3^+^CD8^+^CD44^low^CD62L^high^) were then sorted by FACS sorting (BD FACSAria™ III sorter; the sorting antibody information was already provided in Appendix Table S1). 2.5E5 purified naïve CD8^+^ T cells in 100 µl of PBS (Lonza, BE17‐516F) were injected intravenously into 8–12 week‐old *Rag‐1* ^−/−^ mice. Six weeks post adoptive transfer, CD8^+^ T cells from spleens of the recipients were stained with abs and then acquired by BD LSRFortessa™.

### Microarray analysis of murine T cells

CD25^−^CD4^+^ Tconv cells from ~45‐week‐old *Dj‐1* ^−/−^ mice and *Dj‐1* ^+/+^ littermates were sorted using BD FACSAria^TM^ III sorter. The cell pellets were immediately lysated with RLT buffer supplemented with 1% beta‐mercaptoethanol (Sigma‐Aldrich, 63689) and frozen at −80°C for further analysis. RNA was extracted by using the RNeasy Mini Spin Kit (Qiagen, 74104) and genome DNA was removed. Samples were analyzed via RNA 6000 Pico kit (Agilent, 50671513) by using an Agilent Bioanalyzer 2100, ensuring that only the samples have RIN > 8.5 were further used for microarray measurement.

RNA samples were analyzed with the Affymetrix mouse Gene 2.0 ST Array at EMBL Genomics core facilities (Heidelberg). The microrray data analysis was performed in the same way as described in our previous work (preprint: Danileviciute *et al*, 2019). To ease the reading of this work, we described the major filtering steps here again. The expression signal at the exon level was summarized by the Affymetrix PLIER algorithm with DABG and PM‐GCBG options by means of the sketch‐quantile normalization approach (Affymetrix Expression Console v1.4). The corresponding probesets were considered differentially expressed if they passed the following combinatory filters (Yosef *et al*, 2013): (i) whether the change folds were ≥ 2 between the means of *Dj‐1* KO and WT Tconv; (ii) whether the *P*‐value, resulting from a two‐tailed Student’s *t*‐test, was ≤ 0.05; (iii) whether the cross‐hyb type of the probeset was equal to 1; (iv) whether the probeset with the highest expression level was > 100 (with the median value of ~90 for each our sample); and (v) for a given group (e.g. WT) with the higher mean intensity value of the probeset, whether the probeset in all the replicates of the given group was detected as “present” according to the default setting of the Affymetrix Expression Console. DAVID v6.7 was used to perform functional enrichment analysis for the lists of differentially up‐ or down‐regulated genes (Huang *et al*, 2008). *Dj‐1*‐deficient Tconv cells exhibited much larger fractions of downregulated genes (416) compared with those of upregulated genes (53). This is why we focused on the analysis of the downregulated genes in this work.

### PBMC isolation and flow cytometry analysis of the DJ‐1‐mutant patient family

The experiments conformed to the principles set out in the World Medical Association (WMA) Declaration of Helsinki and the Department of Health and Human Services Belmont Report. We complied with all the relevant ethic regulations and Luxembourg CNER (Comité National d'Ethique de Recherche) has approved the PD patients/participants related study. The family carrying the c.192G>C mutation in the DJ‐1 gene has been already described elsewhere (Boussaad *et al*, 2020) and written informed consent for all participating individuals was obtained. The index patient (56 years) carries the homozygous c.192G>C mutation and has been affected by PD for 22 years. The general disease progression over time is benign with a retained good response to levodopa therapy. The patient was currently presenting with a bilateral akinetic‐rigid syndrome more pronounced on the left side with some postural instability and variable gait problems not interfering with his autonomy and corresponding to a Hoehn&Yahr stage 3. The two unaffected siblings (60 and 63 years) are both heterozygous carriers of the c.192G>C mutation and were devoid of any clinical sign of PD at the recent neurological examination, as expected for this autosomal‐recessively inherited condition. The experimental investigators were blind to the genotype of the three siblings until the flow cytometry analysis was complete. Peripheral blood mononuclear cells (PBMCs) were isolated from the participants’ blood by Ficoll gradient centrifugation using SepMate tubes (StemCell, 86450, also refer to Appendix Table S3) and Lymphoprep (StemCell, 07801). Following three washing steps 1E6 isolated PBMCs per participant for each staining panel were blocked for 15 min using Fc blocking antibodies (BD, 564765), then stained for 30 min at 4°C using brilliant stain buffer (BD, 563794) containing the antibodies against cell surface markers from Appendix Table S4. The cells were washed with human FCM buffer (PBS + 2% FBS, PBS was also Ca^2+^ and Mg^2+^ free; of note, without EDTA, slightly different from the FCM buffer used for mice cell staining) and fixed for 1 h using the True‐Nuclear Transcription factor buffer set (BioLegend, 424401). Following fixation, intracellular markers were stained for 30 min at room temperature in permeabilization buffer. After three washing steps, the cells were re‐suspended in FCM buffer and acquired on a BD Fortessa. The flow cytometry data were analyzed using FlowJo software (v10, Tree Star).

### TCR repertoire sequencing and mRNA microarray of the DJ‐1‐mutant patient family

Fresh PBMCs were stained with anti‐CD4 FITC (BD, 555346, also refer to Appendix Table S4), anti‐CD8 BV605 (BioLegend, 301040), and LIVE/DEAD™ Fixable Near‐IR Dead Cell Stain Kit (Thermo Fisher Scientific, L34976) for 30 min at 4°C. Stained cells were washed with human FCM buffer (PBS + 2%FBS) and total CD8 T cells were sorted using the BD FACSAria™ III. The cell pellets were immediately lysated with RLT buffer supplemented with 1% beta‐mercaptoethanol (Sigma‐Aldrich, 63689) and frozen at −80°C for further analysis. The same procedure was applied for both human and mice cells to extract RNA (refer to the section above). The RIN values were analyzed by RNA 6000 Pico‐kit (Agilent, 50671513) by using an Agilent Bioanalyzer 2100 and all the three samples had a RIN value of 10. Human RNA samples were analyzed with the Affymetrix Human Gene 2.0 ST Array at EMBL Genomics Core Facility (Heidelberg). A very similar procedure (refer to the section above) was applied to analyze both human and murine microarray datasets, with specific consideration of there were only three samples from human samples. The expression signal at the exon level was summarized by the Affymetrix PLIER algorithm with DABG and PM‐GCBG options by means of the sketch‐quantile normalization approach (Affymetrix Expression Console v1.4). The corresponding probesets were considered for further analysis if they passed the combinatory filters below: (ii) the change folds were ≥ 2 between both the two comparisons P2 versus P1 and P2 versus P3; (ii) the cross‐hyb type of the probeset was equal to 1; (iii) the probeset with the highest expression level was > 100; (iv) if the expression of the given probeset was regarded higher in the patient P2, the probeset in P2 had to be detected as “present” according to the default setting of the Affymetrix Expression Console; if the expression of the given probeset was regarded lower in the patient P2, the probeset in both P1 and P3 had to be detected as “present”.

For TCR repertoire analysis, we used cryopreserved PBMCs. PMBCs were cryopreserved in liquid nitrogen in aliquots of 5E6 cells in 1 ml (90% FBS + 10% DMSO). The thawed PBMCs were washed twice with prewarmed (37°C) supplemented complete IMDM [for the exact media components, refer to our previous work (preprint: Danileviciute *et al*, 2019; Capelle *et al*, 2021)] and recovered over night at 37°C, 7.5% CO_2_. Antibodies used to stain for FACS sorting of naïve (CD3^+^CD45RO^−^CD45RA^+^) CD4^+^ or CD8^+^ T cells from participants’ PBMC are listed in Appendix Table S4. Stained cells were washed with FCM buffer (PBS + 2% FBS) and naïve CD4 and CD8 T cells were sorted using the BD FACSAria™ III. DNA was extracted from the flash frozen pellets to perform TCR repertoire sequencing. Genomic DNA (gDNA) was extracted from the sorted naïve CD4 and CD8 T cells using the QIAamp DNA Blood Mini Kit (Qiagen, 51104) following the manufacturer’s instructions. The gDNA was eluted in 55 µl RNase‐ and DNase‐free water to match the volume and concentration requirements for survey analysis of TCR beta repertoire sequencing by ImmunoSEQ (Adaptive Biotechnologies, Seattle, USA). All the analyses (TCR richness estimation and clonality) were performed using the online tool of ImmunoSEQ Analyzer 3.0. The lower bound of TCR repertoire of CD4 Tn and CD8 Tn by applying nonparametric statistics using the iChao1 estimator. The richness of TCR repertoire of CD4 Tn and CD8 Tn by applying a nonparametric empirical Bayes estimation using the Efron‐Thisted estimator (extrapolation value is 120K from ImmunoSEQ Analyzer 3.0). The sample clonality of TCR repertoire of CD4 Tn and CD8 Tn (Clonality is equal to 1 – normalized Shannon’s Entropy).

### Plasma cytokine measurement using the MSD platform

The concentration of the selected eight cytokines (IFN‐γ, IL‐10, IL‐17A, IL‐1β, IL‐4, IL‐5, IL‐6, and TNF‐α) was measured in undiluted plasma samples of the three subjects using a multiplex MSD (Mesoscale Discoveries) U‐plex Biomarker Group 1 (human) assay (MSD, K15067L) in the MSD MESO QuickPlex SQ 120 platform following the manufacturer’s instructions.

### CMV ELISA

The CMV seropositivity was measured in the plasma samples of the three DJ‐mutant participants using the anti‐CMV IgG Human ELISA Kit (Abcam, ab108724) following the manufacturer’s instructions. The plasma was used at a 1:100 dilution.

## Author contributions

NZ designed and performed major parts of the mouse‐related experiments and analyzed mouse‐related data. CMC performed and analyzed all the human and parts of mouse‐related experiments. TK, SC, VT, and AB performed parts of mice‐related experiments. DC and CL supervised parts of mouse‐related experiments. HK, JB, AMW, DB, RK, RB, and MO provided insights and supervised parts of the experiments. NZ and CMC drafted the manuscript. RK, MO, and RB revised the manuscript. FQH conceived and directed the project and revised the manuscript.

## Conflict of interest

The authors F.Q. H., M.O., and R.B. have a pending patent on the DJ‐1 inhibiting treatment on immunoaging‐related diseases. There are no conflicts of financial interests to report for the remaining coauthors.

## Supporting information



Appendix

Expanded View Figures PDF

Source Data for Figure 2

## Data Availability

The microarray data from human CD8 T cells of the DJ‐1 mutation carrier family and 45‐week‐old murine CD4 Tcon cells are deposited in Gene expression Omnibus (GEO) repository with the access code GSE173903 and GSE173904, respectively. Our single‐cell TCR‐beta chain repertoire sequencing data of three siblings are available in ImmuneACCESS (https://clients.adaptivebiotech.com/pub/zeng‐2022‐embor or via doi https://doi.org/10.21417/NZ2022EMBOR).
