# Supplementary material for: DJ‐1 depletion prevents immunoaging in T‐cell compartments
Source: EMBO Rep. 2022 Jan 17;23(3):e53302. doi: 10.15252/embr.202153302 (PMC8892345; doi:10.15252/embr.202153302)
Supplement: Supplementary file 1 — Appendix [file EMBR-23-e53302-s001.pdf]

Appendix:

DJ-1 depletion prevents immunoaging in T-cell compartments

Appendix Table S1. List of mouse-related antibodies used in this work. **Pg 2**

Appendix Table S2. List of other materials used in mouse-related experiments of the study. **Pg 3**

Appendix Table S3. Materials or reagents used for human PBMC isolation and flow cytometry analysis. **Pg 5**

Appendix Table S4. List of antibodies used for sorting or analyzing human T cells. **Pg 5**

Appendix Table S1. List of mouse-related antibodies used in this work.

**Antibody**	**Clone**	**Company**	**Catalogue number**	**Dilution factor and application***
CD16/CD32	2.4G2	BD Biosciences	553141	1:50
CD3-BV421	145-2C11	BD Biosciences	562600	1:200
CD3-APC	17A2	Biolegend	100236	1:200 (sorting)
CD25-APC	PC61	BD Biosciences	557192	1:100 (sorting)
CD25-PE-Cy7	PC61.5	eBioscience	25-0251-82	1:200
CD25-BUV395	PC61	BD Biosciences	564022	1:100(mitotracker)
CD44-PE-Cy7	IM7	BD Biosciences	560569	1:200 (sorting)
CD4-BUV496	GK1.5	BD Biosciences	564667	1:200
CD4-FITC	RM4-5	eBioscience	11-0042-82	1:100 (sorting)
CD4-PE	RM4-5	Biolegend	100512	1:200 (mitotracker)
CD62L-PerCP-Cy5.5	MEL-14	BD Biosciences	560513	1:200 (sorting)
CD8-BUV805	53-6.7	BD Biosciences	564920	1:200
CD8-BV737	53-6.7	BD Biosciences	612759	1:200(mitotracker)
CD8-eFluor450	53-6.7	eBioscience	48-0081-82	1:100 (sorting)
CTLA4-PE	UC10-4B9	eBioscience	12-1522-83	1:100
IFNγ-BV711	XMG1.2	BD Biosciences	564336	1:100
Ki-67-BV605	16A8	BioLegend	652413	1:200
Ki-67-EF450	SolA15	eBioscience	48-5698-82	1:100

PD-1-BV711	J43	BD Biosciences	744547	1:100
KLRG1-PERCP-CY5.5	2F1/KLRG1	Biolegend	138417	1:100
KLRG1-BV785	2F1/KLRG1	Biolegend	138429	1:100
CD45.1-BV785	A20	Biolegend	110743	1:200
CD45.2-BV650	104	Biolegend	109835	1:200
**Markers used for Tvm and CD31 phenotypic analysis**				
CD3e	BV421	BD	562600	1:200
CD31/PECAM-1	FITC	eBioscience	11-0311-82	1:50
CD62L	PerCP/Cyanine5.5	Biolegend	104432	1:200
CD49d	PE	Biolegend	103705	1:100
CD44	PE/Cyanine7	Biolegend	103030	1:200
CD4	APC	eBioscience	17-0041-83	1:200
CD8	BUV737	BD	612759	1:200
**TCR sensitivity analysis markers**				
CD8	BUV737	BD	612759	1:200
CD69	BV605	BD	563290	1:100

*, if not specified, the application is conventional FACS analysis; Specified application is mentioned in the bracket.

Appendix Table S2. List of other materials used in mouse-related experiments of the study.

**Materials**	**Company**	**Cat. Number**
0.5 M EDTA	Sigma-Aldrich	E7889
Agilent RNA 6000 Nano kit	Agilent	5067-1511
Agilent RNA 6000 Pico kit	Agilent	5067-1513
Anti-Mouse CD28, Purified	BD	553294
Beta-Mercaptoethanol	Sigma-Aldrich	M7522

Ca2+ free PBS	Lonza	BE17-516F
CD90.2 MicroBeads, mouse	Miltenyi Biotec	130-049-101
CellTrace™ Violet Cell Proliferation Kit	Thermo Fisher Scientific	C34557
DNAse I	Qiagen	79254
eBioscience™ Foxp3 / Transcription Factor Staining Buffer Set	eBioscience	00-5523-00
Fetal bovine serum (FBS)	Gibco	10270106
Fixation/Permeabilization Solution Kit	BD Biosciences	554714
Glutamax (100X)	Thermo Fisher Scientific	35050061
Golgiplug	BD Biosciences	555029
Golgistop	BD Biosciences	554724
HEPES	Thermo Fisher Scientific	15630080
Ionomycin	Sigma-Aldrich	I0634
LS column	Miltenyi Biotec	130-042-401
MEM non-essential amino acids	Sigma-Aldrich	M7145
Naive CD8a+ T Cell Isolation Kit	Miltenyi Biotec	130-096-543
Penicillin+Streptomycin	Thermo Fisher Scientific	15070-063
PMA	Sigma-Aldrich	P8139
Purified NA/LE Hamster Anti-Mouse CD3e	BD	553057
Red blood cell lysis buffer	BD	555899
RNeasy Mini Spin Kit	Qiagen	74104
RPMI 1640	Lonza	12-167F
Seahorse Fluxpaks	Agilent	102601-100
Seahorse XF base medium	Agilent	102353-100
Seahorse XF Cell Mito Stress Test Kit	Agilent	103015-100
Seahorse XF Glycolysis Stress Test Kit	Agilent	103020-100
Sodium Pyruvate	Thermo Fisher Scientific	11360070

Appendix Table S3. Materials or reagents used for human PBMC isolation and flow cytometry analysis.

Name	Company	Ref. number
SepMate tubes	StemCell	86450
Lymphoprep	StemCell	07801
True-Nuclear Transcription Factor Buffer Set	BioLegend	424401
Brilliant Stain Buffer	BD	563794
UltraComp eBeads	eBioscience	01-2222-42

Appendix Table S4. List of antibodies used for sorting or analyzing human T cells.

Protein	Color	Dilution	Company	Ref. number	Clone
**Analysis staining abs**					
Fc Blocking Abs	/	1:50	BD	564765	/
CD4	BUV395	1:40	BD	563550	SK3 aka Leu3a
CD8	BUV496	1:40	BD	564804	RPA-T8
CD27	APC	1:40	BD	561786	M-T271
CD28	BUV785	1:40	BioLegend	302950	CD28.2
CD45RO	PE-CF594	1:40	BD	562299	UCHL1
CD57	FITC	1:40	BD	555619	NK-1
CD197 (CCR7)	Pacific Blue	1:40	BioLegend	353210	G043H7
Eomes	PE-Cy7	1:20	Thermo Fischer Scientific	25-4877-42	WD1928
FOXP3	APC	1:20	BioLegend	320114	206D
Ki-67	FITC	1:20	BD	561165	B56
PD-1	BV605	1:40	BioLegend	329924	EH12.2H7
T-bet	PE	1:20	BioLegend	644810	4B10
Live/Dead	APC-Cy7	1:500	Thermo Fischer Scientific	L34976	/
**Microarray analysis sorting panel**					
CD4	FITC	1:20	BD	555346	RPA-T4
CD8	BV605	1:20	BioLegend	301040	RPA-T8
Live/Dead	APC-Cy7	1:500	Thermo Fischer Scientific	L34976	/
**TCR repertoire analysis sorting panel**					
Fc Blocking Abs	/	BD Bioscience	/	564765	1:50
CD3	HIT3a	BD Bioscience	BV510	741822	1:100
CD4	RPA-T4	BD Bioscience	FITC	555346	1:100
CD8	RPA-T8	Biolegend	BV605	301040	1:100
CD45RA	HI100	Biolegend	Pacific Blue	304123	1:50
CD45RO	UCHL1	BD Bioscience	PE-CF594	562299	1:50
