# Supplementary material for: DJ‐1 depletion prevents immunoaging in T‐cell compartments
Source: EMBO Rep. 2022 Jan 17;23(3):e53302. doi: 10.15252/embr.202153302 (PMC8892345; doi:10.15252/embr.202153302)
Supplement: Supplementary file 2 — Expanded View Figures PDF [file EMBR-23-e53302-s003.pdf]

Expanded View Figures

Figure EV1. The DJ-1-devoid index patient showed diminished immunoaging features also in CD4 T cells.

A Gating strategy to define CD4 and CD8 T cells in human peripheral blood mononuclear cells (PBMC).
B Percentages of total CD4 and CD8 T cells among the living lymphocyte singlets of peripheral blood of three participates [P1 (heterozygous mutation), P2 (homozygous mutation), and P3 (heterozygous)].
C, D Coexpression of CCR7 and CD45RO (C), CD27 and CD45RO (D) on peripheral blood CD4 T cells of three participants.
E–G Expression of CD57 (E), PD-1 (F), and T-bet (G) on peripheral blood CD4 T cells of three participants.
H Frequency of $FOXP3^{+}CD4^{+}$ Tregs among total CD4 T cells in the peripheral blood from three participants.
I, J Comparison of the lower bound of TCR-beta repertoire (I) and richness (J) of sorted naïve CD4 T cells of three participants.
K The sample clonality index of TCR repertoire of naïve CD4 T cells of three participants.
L–N Cytokine measurement of IFN-γ (L), TNF-α (M), and IL-6 (N) in the plasma of three participants. The dots/symbols in cytokine results represent technical replicates. The other five tested cytokines were either undetectable or below fit curve. For cytokine measurement, data are mean $\pm$ SD.

 © 2022 The Authors

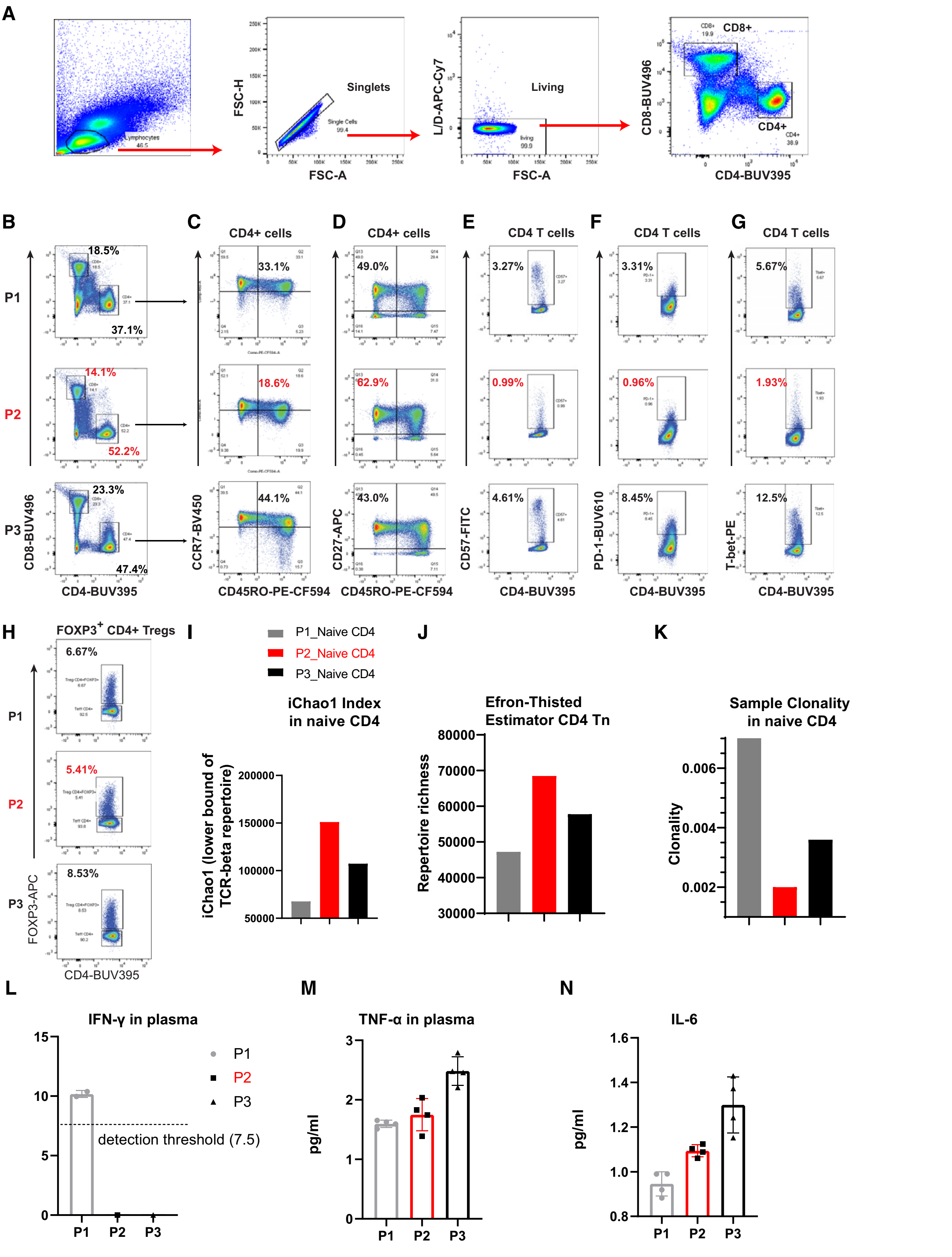

A
FSC-H
FSC-A
Singlets
L/D-APC-Cy7
Living
CD8-BUV496
CD8+
CD4+
CD4-BUV395
B
C
CD4+ cells
D
CD4+ cells
E
CD4 T cells
F
CD4 T cells
G
CD4 T cells
P1
P2
P3
18.5%
37.1%
14.1%
52.2%
23.3%
47.4%
33.1%
18.6%
44.1%
49.0%
62.9%
43.0%
3.27%
0.99%
4.61%
3.31%
0.96%
8.45%
5.67%
1.93%
12.5%
CCR7-BV450
CD45RO-PE-CF594
CD27-APC
CD57-FITC
PD-1-BUV610
T-bet-PE
H
FOXP3+ CD4+ Tregs
6.67%
5.41%
8.53%
FOXP3-APC
I
P1_Naive CD4
P2_Naive CD4
P3_Naive CD4
iChao1 Index in naive CD4
iChao1 (lower bound of TCR-beta repertoire)
J
Efron-Thisted Estimator CD4 Tn
Repertoire richness
K
Sample Clonality in naive CD4
Clonality
L
IFN-γ in plasma
detection threshold (7.5)
M
TNF-α in plasma
pg/ml
N
IL-6
pg/ml

Figure EV1.

Figure EV2. Extended characterization of diminished signs of immunoaging in CD8 T-cell compartments.

A Percentages of $CD44^{high}$ $CD62L^{high}$ cells (Tcm) among total CD8 T cells of spleen and pLNs from young and 45-week-old *Dj-1* KO and WT littermates (young KO, $n = 5$; young WT, $n = 5$; 45-week-old KO, $n = 15$; 45-week-old WT, $n = 7$; for 45-week-old mice, data pooled from 3 independent experiments).

B Percentages of PD-1^{+} cells among splenic CD8 Tn, Tem, and Tcm subsets in the 45-week-old *Dj-1* KO and age- and sex-matched WT mice (45-week-old KO, $n = 11$ and 45-week-old WT, $n = 7$).

C–E Absolute number of CD8 Tn (C), Tmem (D), and Tvm (E) within the acquired 20K of living CD3 T cells from spleen of young or 60-week-old mice (young KO, $n = 4$; young WT, $n = 3$; 60-week-old KO, $n = 4$; and 60-week-old WT, $n = 5$).

F Frequency of CD3 T cells among living singlet lymphocytes from spleen of young or 60-week-old mice (young KO, $n = 4$; young WT, $n = 3$; 60-week-old KO, $n = 4$; and 60-week-old WT, $n = 5$).

G The total number of splenocytes of young or 60-week-old mice (young KO, $n = 4$; young WT, $n = 3$; 60-week-old KO, $n = 4$; and 60-week-old WT, $n = 5$).

H, I Comparison of CD8 Tcm mitochondrial mass (H) and membrane potential (I) of young and 45-week-old *Dj-1* KO and WT mice (young KO, $n = 4$; young WT, $n = 5$; 45-week-old KO, $n = 4$; and 45-week-old WT, $n = 7$). MT, MitoTracker.

Data information: Results represent at least four (A, B), two (C–G), and three (H, I) independent experiments. Data are mean $\pm$ SD. The *P*-values are determined by a two-tailed non-paired Student's *t*-test (A, B, H, and I). Ordinary one-way ANOVA with Sidak's multiple comparison test was applied in C-G. n.s. or unlabeled, not significant, $*P \leq 0.05$, $**P \leq 0.01$, and $***P \leq 0.001$.

© 2022 The Authors

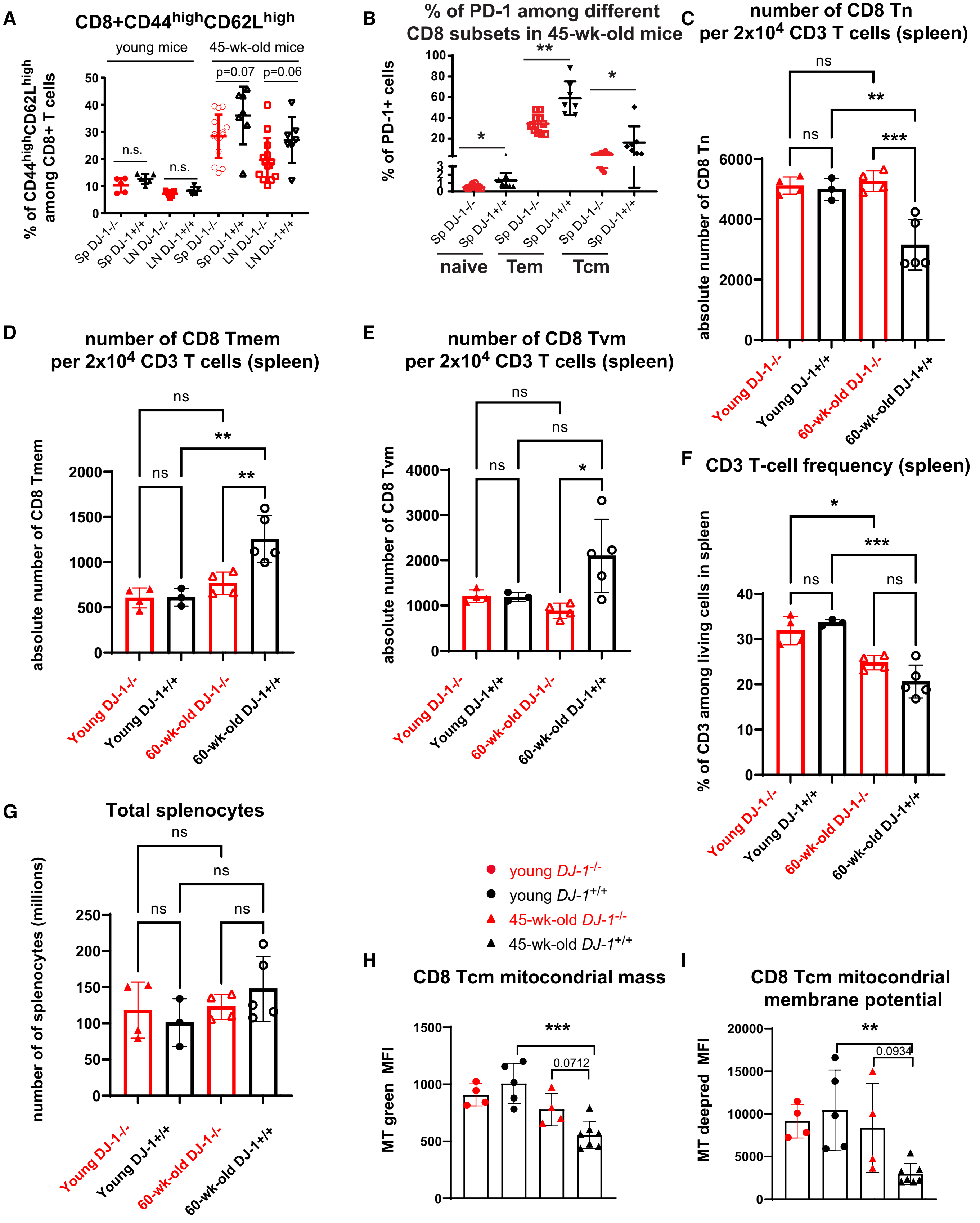
A
CD8+CD44highCD62Lhigh
young mice
45-wk-old mice
n.s.
n.s.
p=0.07
p=0.06
% of CD44highCD62Lhigh among CD8+ T cells
Sp DJ-1-/-
Sp DJ-1+/+
LN DJ-1-/-
LN DJ-1+/+
B
% of PD-1 among different CD8 subsets in 45-wk-old mice
% of PD-1+ cells
naive
Tem
Tcm
C
number of CD8 Tn per 2x10^4 CD3 T cells (spleen)
absolute number of CD8 Tn
D
number of CD8 Tmem per 2x10^4 CD3 T cells (spleen)
absolute number of CD8 Tmem
E
number of CD8 Tvm per 2x10^4 CD3 T cells (spleen)
absolute number of CD8 Tvm
F
CD3 T-cell frequency (spleen)
% of CD3 among living cells in spleen
G
Total splenocytes
number of of splenocytes (millions)
Young DJ-1-/-
Young DJ-1+/+
60-wk-old DJ-1-/-
60-wk-old DJ-1+/+
young DJ-1-/-
young DJ-1+/+
45-wk-old DJ-1-/-
45-wk-old DJ-1+/+
H
CD8 Tcm mitochondrial mass
MT green MFI
0.0712
I
CD8 Tcm mitochondrial membrane potential
MT deepred MFI
0.0934

Figure EV2.

Figure EV3. *Dj-1* depletion also reduced signs of immunoaging in CD4 T-cell compartments.

A Expression level of CD31 among total blood or splenocyte CD4 T cells of 45-week-old (left) and young (right) mice.

B Representative flow-cytometry plots of CD44 and CD62L expression on total CD4 T cells of 45-week-old *Dj-1* KO and age- and sex-matched WT mice (young KO, $n = 5$; young WT, $n = 5$; 45-week-old KO, $n = 8$; 45-week-old WT, $n = 6$; for 45-week-old mice, data pooled from 2 independent experiments; of note, more than one pLNs might be taken from several mice).

C, D Percentages of $CD44^{low}$ $CD62L^{high}$ (Tn) (C) and $CD44^{high}$ $CD62L^{low}$ (Tem) (D) cells among total CD4 T cells of spleen and pLNs from young and 45-week-old *Dj-1* KO and WT littermates.

E Representative histogram overlay of PD-1 expression among total CD4 T cells in spleen of 45-week-old mice (left panel) and percentages of PD-1^+ cells among total CD4 T cells (right panel).

F Representative histogram overlay of CTLA-4 expression among total CD4 T cells in spleen of 45-week-old mice (left panel) and percentages of CTLA-4^+ cells among total CD4 T cells (right panel).

G Percentages of Ki-67^+ cells among total CD4 T cells.

H IFN-γ production in CD4 T cells of spleen and pLNs after *in vitro* stimulation using 50 ng/ml of PMA and 750 ng/ml of ionomycin for 5 h.

I The selected significantly enriched GO-terms and pathways among the downregulated genes in CD4 Tconv cells from 45-week-old *Dj-1* KO mice versus the age- and gender-matched WT littermates from microarray analysis (upper panel). Lower panel, volcano plot shows both downregulated and upregulated differentially expressed genes in splenic CD4 T cells from three 45-week-old *Dj-1* KO mice versus three age-matched WT littermates. The dashed line in y axis corresponds to the value of 1.3 ($P = 0.05$), while the two dashed lines in *x*-axis correspond to −1 and 1 (change fold = 2). A two-tailed Student *t*-test was used to calculate the *P* values (for detailed microarray analysis method, refer to Materials and Methods).

J, K Comparison of naive CD4 (Tn) mitochondrial mass (mito mass, J) and membrane potential (mito potential, K) of young and 45-week-old *Dj-1* KO and WT mice.

L, M Comparison of CD4 Tem mitochondrial mass (mito mass, L) and membrane potential (mito potential, M) of young and 45-week-old *Dj-1* KO and WT mice. SP and LN represent spleen and lymph nodes, respectively.

Data information: results represent at least four (B–G) and three (J–M) independent experiments. Data are mean of biological replicates ± SD. Each biological replicate indicates the measurement from one individual mouse. The *P*-values are determined by a two-tailed un-paired Student's *t*-test. n.s. or unlabeled, not significant, $^*P \le 0.05$, $^{**}P \le 0.01$, $^{***}P \le 0.001$, and $^{****}P \le 0.0001$.

© 2022 The Authors

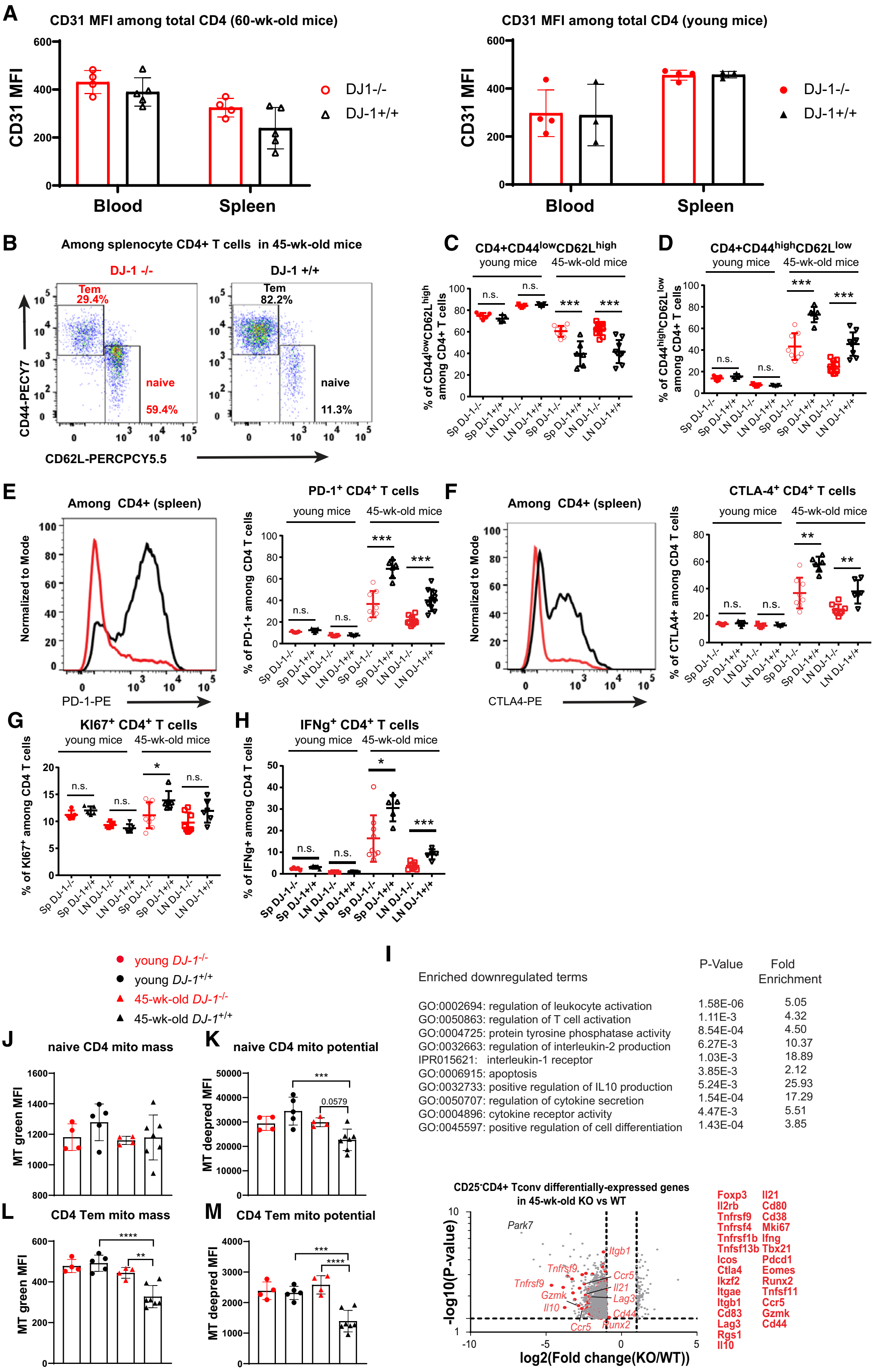
A
CD31 MFI among total CD4 (60-wk-old mice)
CD31 MFI among total CD4 (young mice)
CD31 MFI
Blood
Spleen
DJ1-/-
DJ-1+/+
DJ-1-/-
B
Among splenocyte CD4+ T cells in 45-wk-old mice
DJ-1 -/-
DJ-1 +/+
Tem 29.4%
Tem 82.2%
naive 59.4%
naive 11.3%
CD44-PECY7
CD62L-PERCPCY5.5
C
CD4+CD44lowCD62Lhigh
D
CD4+CD44highCD62Llow
young mice
45-wk-old mice
E
Among CD4+ (spleen)
PD-1+ CD4+ T cells
Normalized to Mode
PD-1-PE
% of PD-1+ among CD4 T cells
F
CTLA-4+ CD4+ T cells
CTLA4-PE
% of CTLA4+ among CD4 T cells
G
KI67+ CD4+ T cells
% of KI67+ among CD4 T cells
H
IFNg+ CD4+ T cells
% of IFNg+ among CD4 T cells
young DJ-1-/-
young DJ-1+/+
45-wk-old DJ-1-/-
45-wk-old DJ-1+/+
I
Enriched downregulated terms
P-Value
Fold Enrichment
GO:0002694: regulation of leukocyte activation 1.58E-06 5.05
GO:0050863: regulation of T cell activation 1.11E-3 4.32
GO:0004725: protein tyrosine phosphatase activity 8.54E-04 4.50
GO:0032663: regulation of interleukin-2 production 6.27E-3 10.37
IPR015621: interleukin-1 receptor 1.03E-3 18.89
GO:0006915: apoptosis 3.85E-3 2.12
GO:0032733: positive regulation of IL10 production 5.24E-3 25.93
GO:0050707: regulation of cytokine secretion 1.54E-04 17.29
GO:0004896: cytokine receptor activity 4.47E-3 5.51
GO:0045597: positive regulation of cell differentiation 1.43E-04 3.85
CD25-CD4+ Tconv differentially-expressed genes in 45-wk-old KO vs WT
-log10(P-value)
log2(Fold change(KO/WT))
Foxp3 Il2rb Tnfrsf9 Tnfrsf4 Tnfrsf1b Tnfsf13b Icos Ctla4 Ikzf2 Itgae Itgb1 Cd83 Lag3 Rgs1 Il10
Il21 Cd80 Cd38 Mki67 Ifng Tbx21 Pdcd1 Eomes Runx2 Tnfsf11 Ccr5 Gzmk Cd44
J
naive CD4 mito mass
MT green MFI
K
naive CD4 mito potential
MT deepred MFI
L
CD4 Tem mito mass
M
CD4 Tem mito potential

Figure EV3.

Figure EV4. *Dj-1* ablation regulated KLRG1 and PD-1 expression as well as the ratios between CD8 Tn and Tem in a hematopoietic-intrinsic manner but the accumulation of CD8 Tn and Tcm in a complicated manner.

A Schematic of the experimental setup of bone marrow transplantation. A total of 10E6 of bone marrow cells from young *Dj-1* KO mice ($CD45.2^+$) and WT mice ($CD45.1^+$) (1:1 mix) were transferred into lethally-irradiated young WT recipients ($CD45.2^+$) by i.v. injection. Mice stably engrafted with donor cells were sacrificed for flow cytometry (FCM) analysis later.

B, C Percentages of $KLRG1^+$ CD8 T cells derived from young *Dj-1* KO and WT donor BM cells in blood (B) and spleen (C) within young WT recipients ($n = 5$; blood sampled twice at both 6 and 8 weeks).

D Percentages of PD-1^+ cells among total CD8 T cells derived from young *Dj-1* KO and WT BM cells in spleen within young WT recipients.

E, F Percentages of CD8 Tem derived from young *Dj-1* KO and WT BM cells in blood (E) and spleen (F) within young WT recipients.

G, H Ratios between CD8 Tn and Tem cells developed from CD45.1 (WT) or CD45.2 (KO) BM cells in blood (G) and spleen (H) within young WT recipients.

I, J Percentages of CD8 Tn in blood (I) and spleen (J) derived from young *Dj-1* KO and WT BM cells within young WT recipients.

K, L Percentage of CD8 Tcm among total CD8 T cells derived from young *Dj-1* KO and WT BM cells in blood (K) and spleen (L) of young WT recipients.

M Percentages of CD8 single-positive cells among thymus originated from 45-week-old *Dj-1* KO and WT BM cells within young *Dj-1* KO or WT recipients following reconstitution.

N, O Percentages of CD8 $CD44^{high}CD62L^{high}$ (Tcm) cells in blood (N) and spleen (O) derived from 45-week-old *Dj-1* KO and WT BM cells within young *Dj-1* KO or WT recipients.

Data information: Results represent two independent experiments. The *P*-values are determined by a two-tailed paired Student's *t*-test. n.s., not significant, $^*P \le 0.05$, $^{**}P \le 0.01$, and $^{***}P \le 0.001$.

 © 2022 The Authors

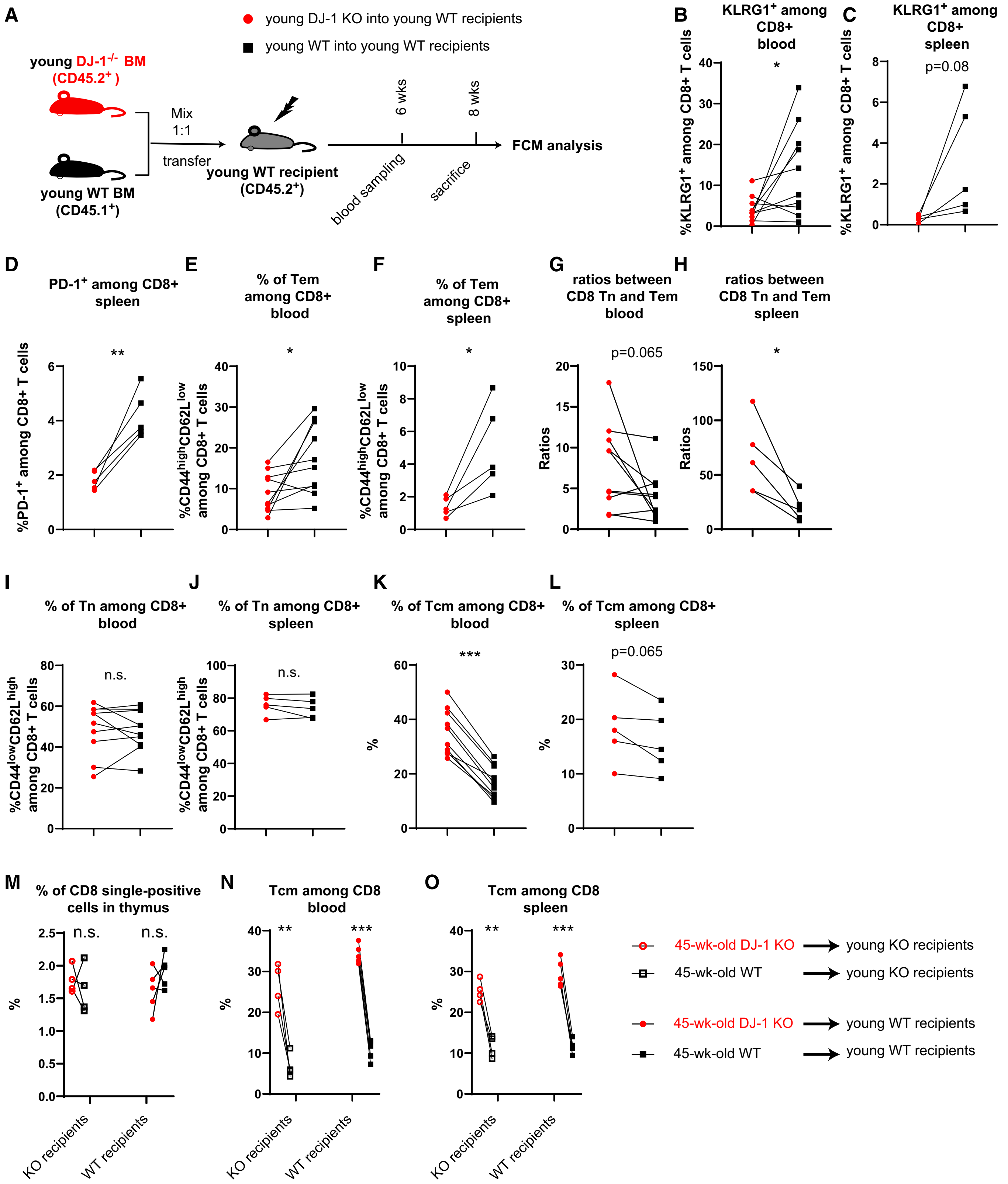
A
young DJ-1 KO into young WT recipients
young WT into young WT recipients
young DJ-1-/- BM (CD45.2+)
young WT BM (CD45.1+)
Mix 1:1
transfer
young WT recipient (CD45.2+)
6 wks
8 wks
blood sampling
sacrifice
FCM analysis
B
KLRG1+ among CD8+ blood
%KLRG1+ among CD8+ T cells
C
KLRG1+ among CD8+ spleen
p=0.08
D
PD-1+ among CD8+ spleen
%PD-1+ among CD8+ T cells
E
% of Tem among CD8+ blood
%CD44highCD62Llow among CD8+ T cells
F
% of Tem among CD8+ spleen
G
ratios between CD8 Tn and Tem blood
p=0.065
Ratios
H
ratios between CD8 Tn and Tem spleen
I
% of Tn among CD8+ blood
%CD44lowCD62Lhigh among CD8+ T cells
n.s.
J
% of Tn among CD8+ spleen
K
% of Tcm among CD8+ blood
L
% of Tcm among CD8+ spleen
p=0.065
M
% of CD8 single-positive cells in thymus
N
Tcm among CD8 blood
O
Tcm among CD8 spleen
KO recipients
WT recipients
45-wk-old DJ-1 KO → young KO recipients
45-wk-old WT → young KO recipients
45-wk-old DJ-1 KO → young WT recipients
45-wk-old WT → young WT recipients

Figure EV4.

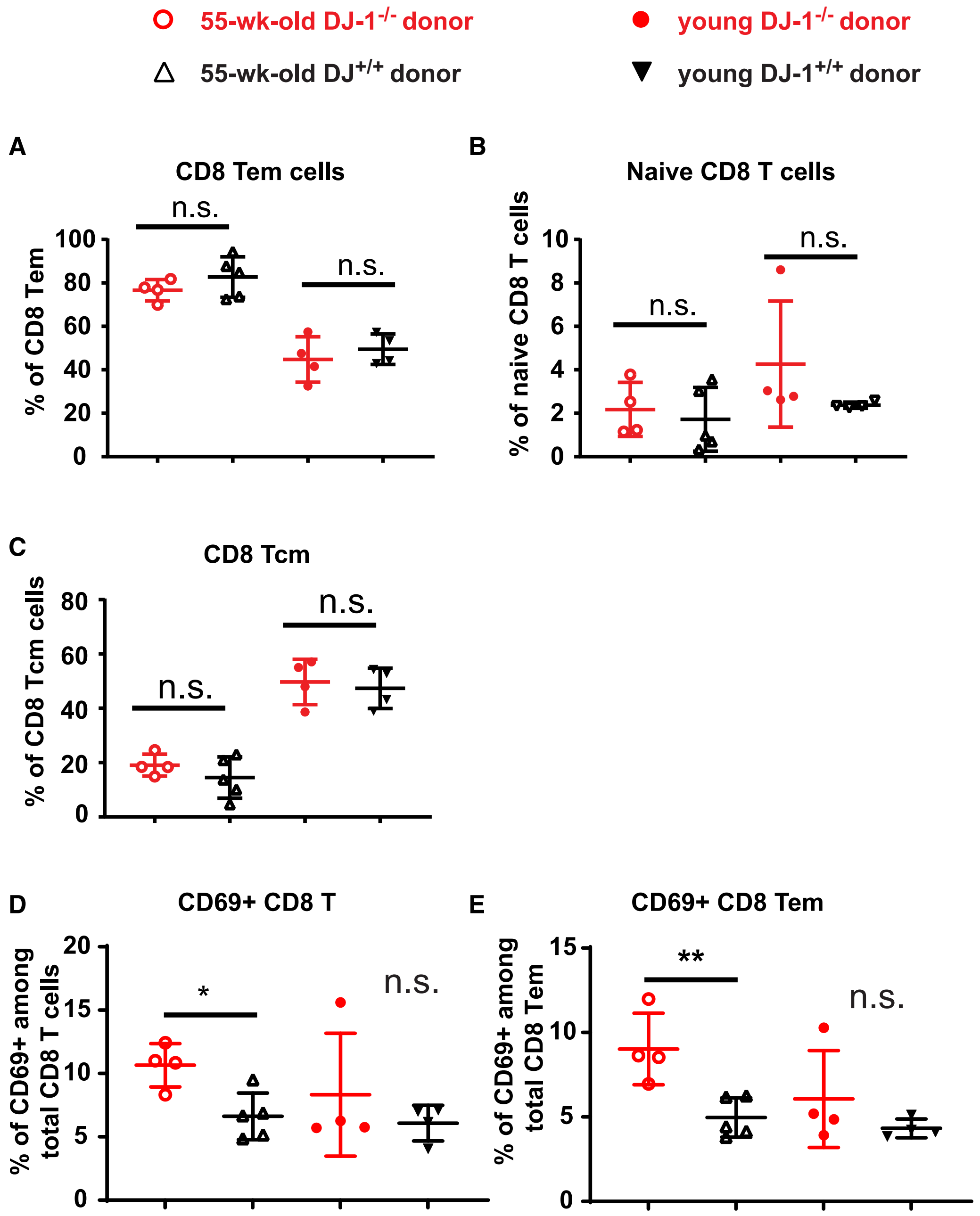

Figure EV5. Extended characterization of CD8 T-cell compartments following adoptive transfer of CD8 Tn into *Rag1*-deficient mice.

A–C Percentages of splenic CD8 Tem (CD44^{high}CD62L^{low}) (A), Tn (CD44^{low}CD62L^{high}) (B), and Tcm (CD44^{high}CD62L^{high}) (C) cells (55-week-old KO, n = 4; 55-week-old WT, n = 5; young KO, n = 4; and young WT, n = 4).

D, E Percentage of CD69^{+} cells among total CD8 T cells (D) and CD8 Tem cells (E) in adoptive transfer experiment.

Data information: Each symbol represents one mouse. Results represent two independent experiments. Data are mean ± SD. The *P*-values are determined by a two-tailed non-paired Student's *t*-test. n.s. or unlabeled, not significant, *$P \leq 0.05$ and **$P \leq 0.01$.

 © 2022 The Authors
