# Supplementary material for: DJ‐1 depletion prevents immunoaging in T‐cell compartments
Source: EMBO Rep. 2022 Jan 17;23(3):e53302. doi: 10.15252/embr.202153302 (PMC8892345; doi:10.15252/embr.202153302)
Supplement: Supplementary file 4 [file EMBR-23-e53302-s002.pdf]

EMBO reports

DJ-1 depletion prevents immunoaging in T-cell compartments

Ni Zeng, Christophe Capelle, Alexandre Baron, Takumi Kobayashi, Severine Cire, Vera Tslaf, Cathy Léonard, Djalil Coowar, Haruhiko Koseki, Astrid Westendorf, Jan Buer, Dirk Brenner, Rejko Krüger, Rudi Balling, Markus Ollert, and Feng Hefeng
DOI: 10.15252/embr.202153302

Corresponding author(s): Feng Hefeng (feng.he@lih.lu)

Review Timeline:

Submission Date:	20th May 21
Editorial Decision:	28th Jun 21
Revision Received:	4th Oct 21
Editorial Decision:	2nd Dec 21
Revision Received:	9th Dec 21
Accepted:	16th Dec 21

Editor: Deniz Senyilmaz Tiebe

Transaction Report:

(Note: With the exception of the correction of typographical or spelling errors that could be a source of ambiguity, letters and reports are not edited. Depending on transfer agreements, referee reports obtained elsewhere may or may not be included in this compilation. Referee reports are anonymous unless the Referee chooses to sign their reports.)

Dear Dr. Hefeng,

Thank you for the submission of your research manuscript to our journal, which was now seen by three referees, whose reports are copied below.

Referees find the proposed role of DJ-1 in immunoaging in principle interesting. However, they also raise significant concerns that need to be addressed for publication here. In particular,

- Functional assays testing the effects of DJ-1 on CD8+ T cells are required to substantiate the claims on reduced immunosenescence (referee #1, major point 2; referee #2, second paragraph).
- Thymic output of aged mice should be assessed (referee #1, major point 4).
- Immunometabolic status of T cell populations needs to be better characterized (referee #1, major point 3).
- Immunophenotypic characterization of patients' CD8+ T cells should be presented in a complete form (referee #1, major point 1).
- The effect of DJ-1 depletion on TVM cell population needs to be investigated (referee #2, paragraph 3).

Should you be able to address these concerns fully, we would like to invite you to revise your manuscript with the understanding that the referee concerns (as in their reports) must be fully addressed and their suggestions taken on board. Please address all referee concerns in a complete point-by-point response. Acceptance of the manuscript will depend on a positive outcome of a second round of review. It is EMBO reports policy to allow a single round of revision only and acceptance or rejection of the manuscript will therefore depend on the completeness of your responses included in the next, final version of the manuscript.

We generally allow three months as standard revision time. As a matter of policy, competing manuscripts published during this period will not negatively impact on our assessment of the conceptual advance presented by your study. However, we request that you contact the editor as soon as possible upon publication of any related work, to discuss how to proceed. Should you foresee a problem in meeting this three-month deadline, please let us know in advance and we may be able to grant an extension.

*** Temporary update to EMBO Press scooping protection policy:
We are aware that many laboratories cannot function at full efficiency during the current COVID-19/SARS-CoV-2 pandemic and have therefore extended our 'scooping protection policy' to cover the period required for a full revision to address the experimental issues highlighted in the editorial decision letter. Please contact the scientific editor handling your manuscript to discuss a revision plan should you need additional time, and also if you see a paper with related content published elsewhere.***

IMPORTANT NOTE: we perform an initial quality control of all revised manuscripts before re-review. Your manuscript will FAIL this control and the handling will be DELAYED if the following APPLIES:
1. A data availability section providing access to data deposited in public databases is missing (where applicable).
2. Your manuscript contains statistics and error bars based on n=2. Please use scatter plots in these cases.

You can submit the revision either as a Scientific Report or as a Research Article. For Scientific Reports, the revised manuscript can contain up to 5 main figures and 5 Expanded View figures. If the revision leads to a manuscript with more than 5 main figures it will be published as a Research Article. In this case the Results and Discussion section should be separate. If a Scientific Report is submitted, these sections have to be combined. This will help to shorten the manuscript text by eliminating some redundancy that is inevitable when discussing the same experiments twice. In either case, all materials and methods should be included in the main manuscript file

Supplementary/additional data: The Expanded View format, which will be displayed in the main HTML of the paper in a collapsible format, has replaced the Supplementary information. You can submit up to 5 images as Expanded View. Please follow the nomenclature Figure EV1, Figure EV2 etc. The figure legend for these should be included in the main manuscript document file in a section called Expanded View Figure Legends after the main Figure Legends section. Additional Supplementary material should be supplied as a single pdf labeled Appendix. The Appendix includes a table of content on the first page with page numbers, all figures and their legends. Please follow the nomenclature Appendix Figure Sx throughout the text and also label the figures according to this nomenclature. For more details please refer to our guide to authors.

Please note that for all articles published beginning 1 July 2020, the EMBO Reports reference style will change to the Harvard style for all article types. Details and examples are provided at
https://www.embopress.org/page/journal/14693178/authorguide#referencesformat

When submitting your revised manuscript, please carefully review the instructions that follow below. Failure to include requested items will delay the evaluation of your revision.

1) a .docx formatted version of the manuscript text (including legends for main figures, EV figures and tables). Please make sure

that the changes are highlighted to be clearly visible.

2) individual production quality figure files as .eps, .tif, .jpg (one file per figure).

3) a .docx formatted letter INCLUDING the reviewers' reports and your detailed point-by-point responses to their comments. As part of the EMBO Press transparent editorial process, the point-by-point response is part of the Review Process File (RPF), which will be published alongside your paper. For more details on our Transparent Editorial Process, please visit our website: https://www.embopress.org/page/journal/14693178/authorguide#transparentprocess
You are able to opt out of this by letting the editorial office know (emboreports@embo.org). If you do opt out, the Review Process File link will point to the following statement: "No Review Process File is available with this article, as the authors have chosen not to make the review process public in this case."

4) a complete author checklist, which you can download from our author guidelines (<http://embor.embopress.org/authorguide>). Please insert information in the checklist that is also reflected in the manuscript. The completed author checklist will also be part of the RPF.

5) Please note that all corresponding authors are required to supply an ORCID ID for their name upon submission of a revised manuscript (<https://orcid.org/>). Please find instructions on how to link your ORCID ID to your account in our manuscript tracking system in our Author guidelines (<http://embor.embopress.org/authorguide>).

6) We replaced Supplementary Information with Expanded View (EV) Figures and Tables that are collapsible/expandable online. A maximum of 5 EV Figures can be typeset. EV Figures should be cited as 'Figure EV1, Figure EV2" etc... in the text and their respective legends should be included in the main text after the legends of regular figures.

- For the figures that you do NOT wish to display as Expanded View figures, they should be bundled together with their legends in a single PDF file called *Appendix*, which should start with a short Table of Content. Appendix figures should be referred to in the main text as: "Appendix Figure S1, Appendix Figure S2" etc. See detailed instructions regarding expanded view here: <http://embor.embopress.org/authorguide#expandedview>.

- Additional Tables/Datasets should be labeled and referred to as Table EV1, Dataset EV1, etc. Legends have to be provided in a separate tab in case of .xls files. Alternatively, the legend can be supplied as a separate text file (README) and zipped together with the Table/Dataset file.

7) We would also encourage you to include the source data for figure panels that show essential data.

Numerical data should be provided as individual .xls or .csv files (including a tab describing the data). For blots or microscopy, uncropped images should be submitted (using a zip archive if multiple images need to be supplied for one panel). Additional information on source data and instruction on how to label the files are available <http://embor.embopress.org/authorguide#sourcedata>.

8) Our journal encourages inclusion of *data citations in the reference list* to directly cite datasets that were re-used and obtained from public databases. Data citations in the article text are distinct from normal bibliographical citations and should directly link to the database records from which the data can be accessed. In the main text, data citations are formatted as follows: "Data ref: Smith et al, 2001" or "Data ref: NCBI Sequence Read Archive PRJNA342805, 2017". In the Reference list, data citations must be labeled with "[DATASET]". A data reference must provide the database name, accession number/identifiers and a resolvable link to the landing page from which the data can be accessed at the end of the reference. Further instructions are available at <http://embor.embopress.org/authorguide#datacitation>.

9) Please make sure to include a Data Availability Section before submitting your revision - if it is not applicable, make a statement that no data were deposited in a public database. Primary datasets (and computer code, where appropriate) produced in this study need to be deposited in an appropriate public database (see <http://embor.embopress.org/authorguide#dataavailability>).

Please remember to provide a reviewer password if the datasets are not yet public.

The accession numbers and database should be listed in a formal "Data Availability " section (placed after Materials & Method) that follows the model below. Please note that the Data Availability Section is restricted to new primary data that are part of this study.

Data availability

The datasets (and computer code) produced in this study are available in the following databases:

- RNA-Seq data: Gene Expression Omnibus GSE46843 (https://www.ncbi.nlm.nih.gov/geo/query/acc.cgi?acc=GSE46843)
- [data type]: [name of the resource] [accession number/identifier/doi] ([URL or identifiers.org/DATABASE:ACCESSION])

*** Note - All links should resolve to a page where the data can be accessed. ***

10) Regarding data quantification, please ensure to specify the name of the statistical test used to generate error bars and P values, the number (n) of independent experiments underlying each data point (not replicate measures of one sample), and the test used to calculate p-values in each figure legend. Discussion of statistical methodology can be reported in the materials and methods section, but figure legends should contain a basic description of n, P and the test applied.
Please note that error bars and statistical comparisons may only be applied to data obtained from at least three independent biological replicates.
Please also include scale bars in all microscopy images.

We would also welcome the submission of cover suggestions, or motifs to be used by our Graphics Illustrator in designing a cover.

I look forward to seeing a revised version of your manuscript when it is ready. Please let me know if you have questions or comments regarding the revision.

Kind regards,

Deniz Senyilmaz Tiebe

Deniz Senyilmaz Tiebe, PhD
Editor
EMBO Reports

Referee #1:

The study by Zeng et al describes as possible role of DJ-1 in regulating the aging of the immune system, by claiming that DJ-1 depletion slows down the aging of T cells compartment of adaptive immunity.
The manuscript is interesting and does suggest a possible new role of DJ-1, but in my opinion, in the actual form it fails to prove a functional role of DJ-1 in immunoaging, as it does not provide any real insight on the mechanisms that link DJ-1 KO to slow down immunosenescence. In some cases, the rationale of the proposed experiments is not completely clear

Major points.

Thera are four major flaws in this manuscript:

- I could not find the "classic" immunophenotypic characterization of patients' CD8+ T cells, i.e. Tn/tcm/Tem/Temra, while it is shown for CD4+ T cells in supplementary data. The authors should show this figure. Is there any difference in the proportion of T cell subsets between the proband, the sibling and age and sex-matched controls?

-The observations on CD8+ T cells are phenotypic but do not demonstrate an actual reduced senescence of T cells. One cannot exclude that these cells are phenotypically "younger" but functionally old. Thus, a functional test is needed on patients' T cells to prove that they are actually less senescent, for example by stimulating T cells in vitro with aCD3/aCD28 followed by division rate quantification via CFSE labeling and analysis of T cell division in T cells subsets.

-The experiments on mitochondria are merely descriptive but do not help understand the role of the organelle on the delayed immunoaging. On the basis of the studies by Pierce et al, which provided evidence of the crucial role of metabolic shift and mitochondrial reorganization in the swittch from Tn to Tm cells, I think it could be much more informative an immunometabolic evaluation of T cell subsets (OCR, ECAR) in patients, as well in mouse models. I suggest performing this experiment before and after T cell stimulation, to clarify if there is an impairment in T cell activation that can explain the lower levels of cells with exhausted or senescent phenotype.

-I do not understand the rationale below the choice of performing BM transplantation. As well established in the past, one of the most important causes of T cell compartment senescence is thymus involution, whose progressive shrinking does not allow

proper development of new naïve T cells. Have the authors monitor parameters related to thymic output, such as CD31 expression or sjTREC+ T cells in aged mice? Without this element, it is hard to understand if the effect of DJ-1 is a general effect on the immune system or a specific effect on T cells.

Minor points

-I strongly suggest not to overlap the use of exhausted and senescent throughout the manuscript. Although overlapping, the two concepts are clearly different, as well described by Xu and Larbi (2017), and sliding from one to the other does not help the comprehension of the message.

-Since I do not see the presence of CD3 Ab in the list of Abs used for analysing human T cells, I think it is necessary to show the gating strategy for the immunophenotype.

- In the extended data figure 1, the percentage of T CD4+ and CD8+ T cells are calculated as a fraction of what? T cells? lymphocytes as determined by physical parameters?

-In extended data figure 1, the levels of different cytokines are measured. Since detection threshold of IFNG is 7.5 pg/ml, how have the values of P2 and P3 - clearly below this threshold - been detected?

- There are some typos in the text. Please be consistent in how molecules are named in the text (for example IFN-g, not IFNg

- I understand that we are in COVID-19 pandemic era, but I think it is superfluous to cite COVID-19 in a paper that has nothing to do with it.

Referee #2:

The study begins with an index case containing a homozygous DJ-1 mutation that has driven early onset Parkinson's disease. Previous work had shown that DJ-1 mutation resulted in diminished Treg cells in aged but not young mice. Further analysis of an aged phenotype in this individual revealed a reduced frequency of senescent CD4 and CD8 T cells, reduced expression of markers associated with senescence, differentiation or exhaustion, and reduced TCR narrowing, compared to two unaffected siblings.
To further probe the DJ-1 deficiency, global DJ-1 KO mice were used. Similar to the human phenotype, DJ-1 KO mice show a retention of Tn cells and a reduction of Tem cells with age and a general reduction in markers and characteristics associated with aging, i.e. markers of exhaustion and senescence, and markers of terminal differentiation. Notably, the amelioration of the aged phenotype mediated by loss of DJ-1 was only observed in aged mice and not young mice, and was largely found, via BM chimeras, to be a cell-intrinsic effect of DJ-1 KO. Ostensibly, these data appear to support the contention that DJ-1 plays a role in mediating age-related effects on CD4 and CD8 T cells, with removal of DJ-1 restoring, at least phenotypically, these cells to a younger status.

It is concerning to me that there is no analysis of sensitivity of these cells to stimulation, either in aged or young mice. Given that age-related senescence and/or exhaustion in memory phenotype cells (both antigen-experienced memory cells and inexperienced virtual memory cells) is thought to be due to prolonged or chronic stimulation, it seems possible that the DJ-1 KO cells are refractory to stimulation either through the TCR or cytokine stimulation, which may indirectly prevent an ageing phenotype. This appears to be supported by the transcriptional analysis of Tconv CD4+ cells (Extended data Fig 3I), the mixed bone marrow chimera experiments showing reduced Tem cells, and also by reduced expression of KLRG-1, which, it should be noted is not so much known as an immunoaging marker as a marker of activation and terminal differentiation. Also, the overall reduction in Ki67+ cells also suggests a loss of sensitivity to either tonic TCR stimulation or homeostatic cytokine stimulation. The authors should stimulate young WT and DJ-1 KO cells through the TCR (anti-CD3 rather than PMA/ionomycin) and via cytokines to investigate this. Similarly, if the authors are suggesting that loss of DJ-1 ameliorates an ageing phenotype, this should be analysed functionally, by stimulating KO and WT cells and assessing proliferation, in addition to the phenotypic analysis.

It seems an obvious T cell population to analyse in the context of ageing is the virtual memory population (TVM cells; CD44hi CD49dlo) which have been shown to significantly increase proportionally with age and are contained within the conventional TCM population (CD44hi CD62Lhi). Moreover, of the antigen-inexperienced population, they contain the vast majority of the age-related proliferative T cell dysfunction and express multiple markers of senescence (Quinn et al, Cell Rep 2018). It would be

interesting to know the impact of the DJ-1 KO on the Tvm population.

The mixed BM chimera data (ext data fig 4) is unclear to me. It seems the mixed BM chimeras were used to try to distinguish whether the changes observed in the global DJ-1 knockouts were intrinsic or extrinsic to the haematopoietic compartment. However, firstly, changes were observed in the young BM chimeras that were not observed in the young global knockouts. It is difficult to understand how this can be interpreted as suggesting a "hematopoietic-intrinsic role for regulating ... the expression of immunoaging related markers" given this was done in young mice. Again, generally speaking and especially in young mice, KLRG-1 and PD-1 are not immunoaging-related markers; they are markers of activation and terminal differentiation. I presume that this statement "...DJ-1 exhibits an aging-BM-independent.... role ..." is because shifts from WT were observed whether or not the DJ-1 KO BM used was from young or old mice. But it is difficult to know what this means given that such changes were not observed in young global DJ-1 KO mice.

The transfer of aged BM assesses the impact of DJ-1 only in cells that are generated de novo in old age and ignores the likelihood that much of the T cell ageing phenotype typically observed is driven by prolonged T cell survival and exposure to stimuli over an extended period of time. This is particularly relevant if loss of DJ-1 makes cells more refractory to stimulation.

It is not clear to me why the mixed BM chimera results with respect to the Tn cells in particular were interpreted as either a haematopoietic-extrinsic or aging dependent phenomena. It is already clear from Figure 2b that the changes in Tn in DJ-1 KO mice are aging related. So given the mixed BM chimeras were young, one would not have expected a shift in relative percentages of WT and DJ-1 KO Tn cells. In light of this, I don't understand why the "haematopoietic-extrinsic" interpretation is being suggested.

The study makes an interesting observation regarding a level of maintenance of mitochondrial mass and membrane potential in aged DJ-1 KO mice that ostensibly aligns with the other markers of aging. However, this appears to be at odds with observations that mitochondrial mass increases in T cells with age (Quinn et al, Nat Commun, 2020). This should be discussed and relevant literature cited.

In summary, the study clearly shows a phenotype of DJ-1 knockout, however it should investigate whether the amelioration of the ageing phenotype by DJ-1 KO is manifest functionally as well as phenotypically, should investigate the impact on TVM cells, and should discern whether it is secondary to an overall reduced sensitivity to stimulation by TCR and/or cytokines.

Minor Points

The analysis of Ki67 staining in homeostatic conditions (Fig 2) doesn't address the question of T cell activation vs cell exhaustion, as stated. Homeostatic proliferation is not a reflection of activation. This analysis investigates whether homeostatic proliferation might be responsible for elevated activation/exhaustion markers. This should be reworded.

The following sentence is a bit misleading, "Our recent work have demonstrated that DJ-1 depletion (Bonifati et al. 2003) reduced CD4 regulatory T cells (Treg) cellularity only in aged, but not young adult mice (Danileviciute et al. 2019)." The recent work referred to is Danileviciute et al and not Bonifati et al. Please amend.

Note that the Britanova et al, 2014 paper cited does not analyse TCR repertoire of naïve cells, but rather total cells from naïve individuals. Thus, the reference does not support the statement "During aging, the TCR repertoire diversity decreases in naïve T cells." and should be removed.

Recommend editing the title for Extended Data Fig 3 for grammar.

Y axis on extended data Fig 4f needs to be fixed.

Referee #3:

In the submitted manuscript 'DJ-1 depletion slows down immunoaging in T-cell compartments', Zeng and colleagues report on the implication of loss of DJ-1 on the T lymphocyte compartment during aging in mouse and human. DJ-1 deficiency is correlated with a reduction in several signs of immunoaging related to T cells, with respect to prevalence of certain subpopulations to the expression of immunosenescence marker.

All in all, this is a great example of combining clinical findings with genetic model systems for further validation with significant interest for the general readers interested in aging. DJ-1 had not been put into context of immunoaging. The manuscript is well-written and the results are convincingly reported. I recommend this manuscript to be published after revising some minor points:

Minor comments

1. The authors need to make sure that it is clear in any case (esp. in the abstract, introduction and discussion/summary paragraph), that they refer to immunoaging in the T lymphocyte compartment, as the effect on myeloid cells has not been studied.

2. Please note that 'gender' refers to your own perceived sexual identity. The authors should replace gender in all incidences with 'sex'.

3. The authors use the term 'delay' to describe the found phenotypes related to immunoaging. Can this be specified how long this is? Otherwise, I would suggest to write about the 'diminished signs of immunoaging'.

4. Please cite and discuss the literature on DJ-1 and T cells, e.g. PMID 24953490 reporting on increased IFNg levels in DJ-1 KO Th1 cells.

5. End of 3rd paragraph: 'No increase or decrease trend was observed in systematic levels of relevant pro-inflammatory cytokines...' This should probably read 'systemic' if I am not mistaken.

6. Page 4, last paragraph: '...we have also observed delayed immunoaging features in CD4 T cells of the index patent.' This should probably read 'patient'.

Referee #1:

The study by Zeng et al describes as possible role of DJ-1 in regulating the aging of the immune system, by claiming that DJ-1 depletion slows down the aging of T cells compartment of adaptive immunity.
The manuscript is interesting and does suggest a possible new role of DJ-1, but in my opinion, in the actual form it fails to prove a functional role of DJ-1 in immunoaging, as it does not provide any real insight on the mechanisms that link DJ-1 KO to slow down immunosenescence. In some cases, the rationale of the proposed experiments is not completely clear

Major points.

Thera are four major flaws in this manuscript:

- I could not find the "classic" immunophenotypic characterization of patients' CD8+ T cells, i.e. Tn/tcm/Tem/Temra, while it is shown for CD4+ T cells in supplementary data. The authors should show this figure. Is there any difference in the proportion of T cell subsets between the proband, the sibling and age and sex-matched controls?

Reply: Thanks for pointing out this critical issue. We now provided the FACS plots for the expression of CD45RO and CCR7 as well as that of CD45RO and CD27 in Figure 1a, b of the revised version. As shown in the figure, according to those classic markers, the proband vs the two siblings had indeed much more naïve CD8 T cells, but fewer TEM CD8 cells, which is clearly in line with what we observed in aged DJ- KO mice and other patient's data. We also noticed that the proband had much fewer CD8 TEMRA (we referred to CD45RO-CCR7-CD27-) and more TCM cells. Now, the data should be more complete and even more convincing. We also added one more sentence in line 84-88 and page 3 to describe these results.

-The observations on CD8+ T cells are phenotypic but do not demonstrate an actual reduced senescence of T cells. One cannot exclude that these cells are phenotypically "younger" but functionally old. Thus, a functional test is needed on patients' T cells to prove that they are actually less senescent, for example by stimulating T cells in vitro with aCD3/aCD28 followed by division rate quantification via CFSE labeling and analysis of T cell division in T cells subsets.

Reply: Thanks for raising this constructive suggestion. It is not possible for us to easily access the fresh blood of those patients anymore. Instead, we now isolated naïve CD8 T cells from young and aged DJ-1 KO or WT mice, labelled with Celltrace Violet (known as CTV; similar to CFSE, but less cytotoxic), stimulated with different doses of CD3 ab and measured the proliferation and activation status. Notably, in line with reduced immunoaging notion, the aged naïve DJ-1 KO T cells vs. WT T cells proliferated more (as indicated by CTV peaks) with higher expression of stimulation markers (e.g., CD69) at the low aCD3 concentrations (1 and 2 ug/ml), while the difference, although still significant, gradually disappeared at higher aCD3 concentrations (new Figure 4). Inversely, the young naïve DJ-1 KO T cells vs. WT T cells showed less proliferation and lower levels of activation at the concentration of 1 and 2 ug/ml. A dose-dependent effect indicates that the sensitivity of DJ-1 KO CD8 Tn to TCR stimulation

was compromised, not completely lost. These results indicate that the DJ-1 KO naïve CD8 T cells in young mice are less sensitive to TCR stimulation. Multiple rounds of stimulation (https://pubmed.ncbi.nlm.nih.gov/10530789/) or extensive homeostatic proliferation (https://www.ncbi.nlm.nih.gov/pmc/articles/PMC7150735/), as implicated during the natural aging process, might eventually cause T cell senescence. The compromised TCR sensitivity of DJ-1 KO CD8 Tn generated at a younger age indicates that a reduced activation could accumulate fewer overall homeostatic replications during the long-lasting natural aging process. After reaching a certain age, those long-lived CD8 Tn (https://pubmed.ncbi.nlm.nih.gov/18420820/) with fewer accumulated replication rounds would still have a higher proliferation potential when stimulated.

We have also discussed this point in line 270-285, page 8.

-The experiments on mitochondria are merely descriptive but do not help understand the role of the organelle on the delayed immunoaging. On the basis of the studies by Pierce et al, which provided evidence of the crucial role of metabolic shift and mitochondrial reorganization in the swittch from Tn to Tm cells, I think it could be much more informative an immunometabolic evaluation of T cell subsets (OCR, ECAR) in patients, as well in mouse models. I suggest performing this experiment before and after T cell stimulation, to clarify if there is an impairment in T cell activation that can explain the lower levels of cells with exhausted or senescent phenotype.

Reply: Thanks for this important suggestion. Following this suggestion, we now put great efforts to isolate naïve CD8 T cells (Tn) from young and aged mice and performed Mito stress and glycolysis stress tests with and without anti-CD3/-CD28 stimulation for 24h. From aged mice, stimulated DJ-1 KO vs WT CD8 Tn showed not only reduced mitochondrial OXPHOS (both basal and maximal levels), but also reduced glycolysis and glycolytic capacity (new Fig. 5 generated during revision).The OXPHOS levels were already lower in DJ-1 KO CD8 Tn compared to WT CD8 Tn in the unstimulated naïve T cells. The overall levels of ECAR were very low and therefore did not show a clear difference between DJ-1 KO and WT naïve T cells, possibly due to the fact that unstimulated naïve T cells mainly rely on the OXPHOS pathway.

From young mice, we could only observe a clear reduction in OXPHOS (both basal and maximal respiratory capacity) in unstimulated DJ-1 KO vs WT CD8 Tn. Mitochondrial respiratory capacity is critical for memory CD8 T cell development (van der Windt et al, 2012). The reduced OXPHOS in resting DJ-1 KO CD8 Tn and impaired TCR sensitivity at a younger age, accumulatively result in more remaining proliferation potential of those long-lived CD8 Tn and consequently lead to a less senescent phenotype in T-cell compartments after reaching a critical age.

On the other hand, the enhanced TCR sensitivity of naïve CD8 T cells we observed in aged mice correlated well with enhanced mitochondrial mass and membrane potential, rather than impaired metabolic functions. In line with our puzzling observations, Quinn et al recently also showed that metabolic characteristics not necessarily predict CD8 T-cell functional levels (Quinn et al, Nature Communications, 2020). We have now added several paragraphs to describe and discuss those metabolism results in page 9-11.

-I do not understand the rationale below the choice of performing BM transplantation. As well established in the past, one of the most important causes of T cell compartment senescence is thymus involution, whose progressive shrinking does not allow proper development of new naïve T cells. Have the authors monitor parameters related to thymic output, such as CD31 expression or sjTREC+ T cells in aged mice? Without this element, it is hard to understand if the effect of DJ-1 is a general effect on the immune system or a specific effect on T cells.

Reply: We now analyzed CD31 expression in both CD4 and CD8 T cells. CD31, as a marker of recent thymic emigrant cells, is fully established for CD4 T cells. As expected, the CD31 expression level was lower in aged vs. younger mice, no matter which genotype group. However, no matter from young and aged mice, we cannot observe a clear difference in CD31 expression among CD4 T cells between DJ-1 KO and WT mice. Therefore, the higher proportion of naïve T cells was not simply attributable to a higher thymic outcome in aged DJ-1 KO mice.

It has been recently shown that not only naïve, but also central memory T cells expressed CD31 among CD8 T cells (Newman, J Leukoc Biol, 2018). Interestingly, the expression level of CD31 was higher in aged DJ-1 KO vs WT CD8 total T cells. This is very much in line with our observation of higher frequency of naïve CD8 T cells in aged mice. We now also add one paragraph in the text (line 156-168, page 5).

Minor points

-I strongly suggest not to overlap the use of exhausted and senescent throughout the manuscript. Although overlapping, the two concepts are clearly different, as well described by Xu and Larbi (2017), and sliding from one to the other does not help the comprehension of the message.

Reply: Thanks for this constructive suggestion. We fully agree with the reviewer that exhaustion and senescence are two different concepts and should not have been exchanged or overlapped in using these two terms. In fact, this is one of the essential reasons why we use “Immmunoaging”, rather than “immunosenescence” in the title because the former includes both immune aspects happening during the aging process. In any case, we now removed “immunosenescence” in several places, such as the first sentence of the main text, to avoid giving the wrong impression that we have been essentially discussing immunosencescence. We also now cited the paper from Xu and Larbi (2017) to emphasize this aspect. Throughout the manuscript, we now consistently made all the statements about

“immunoaging”, but not “immunosenescence”. In this way, our statements should be more precise.

-Since I do not see the presence of CD3 Ab in the list of Abs used for analysing human T cells, I think it is necessary to show the gating strategy for the immunophenotype.

Reply: Yes, several years ago, when we analyzed the patient samples, we did not include CD3 ab. Instead, we used the FSC and SSC to only gate on lymphocytes. We now provided the full gating strategy for the patient analysis results in Supplementary Figure 1a.

- In the extended data figure 1, the percentage of T CD4+ and CD8+ T cells are calculated as a fraction of what? T cells? lymphocytes as determined by physical parameters?

Reply: The percentages of CD4 and CD8 T cells were calculated as a fraction of living lymphocyte singlets. As replied to the point above, we now not only provided the full gating strategy in Supplementary Figure 1a, but also updated the legend of Supplementary Figure 1 to make it more clear.

-In extended data figure 1, the levels of different cytokines are measured. Since detection threshold of IFNG is 7.5 pg/ml, how have the values of P2 and P3 - clearly below this threshold - been detected?

Reply: Thanks for pointing out this mistake. We now reset the IFNg value as zero for P2 and P3 since they were below the detection threshold. In the original version, we should not have calculated IFNg values based on the standard curves since the readouts were lower than the detection thresholds.

- There are some typos in the text. Please be consistent in how molecules are named in the text (for example IFN-g, not IFNg

Reply: We now systemically corrected this type of nomenclature issues throughout the text. For example, we now consistently use IFN-γ, rather than anything else. We now use IL-2, rather than IL2 and so on.

- I understand that we are in COVID-19 pandemic era, but I think it is superfluous to cite COVID-19 in a paper that has nothing to do with it.

Reply: We now removed it and the related reference, although we would like to point out that the original purpose is to mention the potential clinical relevance of this work in infectious diseases, including COVID-19. But we agreed with the reviewer that this is not necessary.

Referee #2:

The study begins with an index case containing a homozygous DJ-1 mutation that has driven early onset Parkinson's disease. Previous work had shown that DJ-1 mutation resulted in diminished Treg cells in aged but not young mice. Further analysis of an aged phenotype in this individual revealed a reduced frequency of senescent CD4 and CD8 T cells, reduced expression of markers associated with senescence, differentiation or exhaustion, and reduced TCR narrowing, compared to two unaffected siblings.
To further probe the DJ-1 deficiency, global DJ-1 KO mice were used. Similar to the human phenotype, DJ-1 KO mice show a retention of Tn cells and a reduction of Tem cells with age and a general reduction in markers and characteristics associated with aging, i.e. markers of exhaustion and senescence, and markers of terminal differentiation. Notably, the amelioration of the aged phenotype mediated by loss of DJ-1 was only observed in aged mice and not young mice, and was largely found, via BM chimeras, to be a cell-intrinsic effect of DJ-1 KO. Ostensibly, these data appear to support the contention that DJ-1 plays a role in mediating age-related effects on CD4 and CD8 T cells, with removal of DJ-1 restoring, at least phenotypically, these cells to a younger status.

It is concerning to me that there is no analysis of sensitivity of these cells to stimulation, either in aged or young mice. Given that age-related senescence and/or exhaustion in memory phenotype cells (both antigen-experienced memory cells and inexperienced virtual memory cells) is thought to be due to prolonged or chronic stimulation, it seems possible that the DJ-1 KO cells are refractory to stimulation either through the TCR or cytokine stimulation, which may indirectly prevent an ageing phenotype. This appears to be supported by the transcriptional analysis of Tconv CD4+ cells (Extended data Fig 3I), the mixed bone marrow chimera experiments showing reduced Tem cells, and also by reduced expression of KLRG-1, which, it should be noted is not so much known as an immunoaging marker as a marker of activation and terminal differentiation. Also, the overall reduction in Ki67+ cells also suggests a loss of sensitivity to either tonic TCR stimulation or

homeostatic cytokine stimulation. The authors should stimulate young WT and DJ-1 KO cells through the TCR (anti-CD3 rather than PMA/ionomycin) and via cytokines to investigate this. Similarly, if the authors are suggesting that loss of DJ-1 ameliorates an ageing phenotype, this should be analysed functionally, by stimulating KO and WT cells and assessing proliferation, in addition to the phenotypic analysis.

Reply: We really appreciate this critical point raised by the reviewer. We also acknowledge the reasoning provided by the reviewer, which indeed helps our thinking a lot. To address this critical point, we now isolated naïve CD8 T cells from young or aged DJ-1 KO or WT mice, labelled with Celltrace Violet (CTV, similar to CFSE, but with less cytotoxic) and stimulated them with different doses of CD3 ab. In line with several datasets we already have generated and the reviewer's reasoning, from young mice, naïve DJ-1 KO CD8 T cells vs WT CD8 T cells were less sensitive to tonic TCR stimulation (at least at the dose of 1ug/ml and 2ug/ml, refer to the new Figure 4). With higher doses (such as 4ug/ml or more), the effect disappeared, indicating that the TCR sensitivity was only compromised, but not completely lost. These results indicated a compromised sensitivity of CD8 T cells, which eventually causes a reduced accumulation of TEM, but more naïve T cells leftover during the long-lasting natural aging process.

Notably and unexpectedly, from aged mice, DJ-1 KO vs WT CD8 Tn showed enhanced TCR sensitivity at the dose of 1ug/ml and 2ug/ml (new Fig. 4 generated during revision). At first glance, we thought the results were wrong. After repeating the experiments and critical thinking, we think the results are reasonable. The compromised TCR sensitivity of DJ-1 KO CD8 Tn generated at a younger age would reduce activation and accumulate fewer overall homeostatic replication times of the DJ-1 KO CD8 Tn during the natural aging process. After reaching a critical age, those long-lived CD8 Tn (Vrisekoop et al, 2008) that were generated at a younger age would keep a higher proliferation potential. We have now added one paragraph in line 270-285, page 8 and discussed it in several places and in the end of the text (page 11).

It seems an obvious T cell population to analyse in the context of ageing is the virtual memory population (TVM cells; CD44hi CD49dlo) which have been shown to significantly increase proportionally with age and are contained within the conventional TCM population (CD44hi CD62Lhi). Moreover, of the antigen-inexperienced population, they contain the vast majority of the age-related proliferative T cell dysfunction and express multiple markers of senescence (Quinn et al, Cell Rep 2018). It would be interesting to know the impact of the DJ-1 KO on the Tvm population.

Reply: We also thanks the reviewer for proposing this constructive suggestion. We now particularly analyzed the CD8 TVM subset by co-staining CD44 and CD49d in both blood and spleen from young and aged mice. Encouragingly, in line with our notion of reducing immunoaging, the fraction of TVM , which increases with age, was indeed still lower in both blood and spleen of aged DJ-1 KO vs WT mice. But this difference does not appear yet in young mice (Refer to Figure 2 i, j, generated during the revision), again indicating the aging-dependent phenotype as we observed through this work. With this more strict gating strategy after excluding antigen-inexperienced TVM, the naïve CD8 T cells still showed much higher frequency in aged DJ-1 KO mice relative to WT mice. We also now cited work from Quinn et al (Cell report, 2018) to discuss the related results. We also now added one paragraph in line 147-155, page 4 to describe and discuss these results.

The mixed BM chimera data (ext data fig 4) is unclear to me. It seems the mixed BM chimeras were used to try to distinguish whether the changes observed in the global DJ-1 knockouts were intrinsic or extrinsic to the haematopoietic compartment. However, firstly, changes were observed in the young BM chimeras that were not observed in the young global knockouts. It is difficult to understand how this can be interpreted as suggesting a "hematopoietic-intrinsic role for regulating ... the expression of immunoaging related markers" given this was done in young mice. Again, generally speaking and especially in young mice, KLRG-1 and PD-1 are not immunoaging-related markers; they are markers of activation and terminal differentiation. I presume that this statement "...DJ-1 exhibits an aging-BM-independent.... role ..." is because shifts from WT were observed whether or not the DJ-1 KO BM used was from young or old mice. But it is difficult to know what this means given that such changes were not observed in young global DJ-1 KO mice.

Reply: Thanks for your detailed explanation on your concern. We now revised that sections and discussed several aspects in the text (line 188-195, page 5-6) to make it clearer. We also briefly addressed and discussed some essential points here.

If we directly compared the data of BM chimeras from young mice with young DJ-1 whole-body knockouts, it is indeed difficult to understand the meaning of such changes. BM donor and recipient mice were 8-12-wk old at the time of transplantation. After 8 weeks of reconstitution period, the recipients had already reached an age of ~20-wk old (Figure EV4a), almost half way towards 45-wk old (the age at which, the mice were regarded "aged" in our work).

Furthermore, as demonstrated by others (Cho et al., *JEM*, 2000), the development of CD8 Tem is radically accelerated under lymphopenia (adoptive transfer of naïve CD8 T cells to Rag1 KO recipient mice). We believe such an accelerated homeostatic development process of CD8 TEM, could be extended to the BM model we used in this Figure, where lethal irradiation has been implemented (Figure EV4). Therefore, we expect at least a partial aging phenotype in the BM chimera model, although derived from young donors. The already reduced frequency of CD8 TEM in young BM chimeras might still make sense and can already partially represent the reduced immunoaging phenotypes we observed in aged DJ-1 KO mice. At the same time, we did not observe any clear change in the frequency of CD8 naïve T cells in young BM chimeras, while aged BM chimeras (in KO recipients) already showed a significant change in CD8 naïve T cells. Therefore, the divided results from young BM chimeras have already partially reflected the observation in aged DJ-1 KO mice.

We also very much agree with the reviewer that in normal young mice, KLRG1 and PD-1 represent more status of activation and terminal differentiation. As we just argued above, in the models of accelerated memory development and within 20-week old mice, those markers might not necessarily only represent activation or terminal differentiation status anymore.

Furthermore, also because of the divided results in young BM chimeras, we moved forward to use aged BM chimeras in our work. Without those encouraging results from young BM chimeras, we might not have moved further to use aged BM chimeras.

The transfer of aged BM assesses the impact of DJ-1 only in cells that are generated de novo in old age and ignores the likelihood that much of the T cell ageing phenotype typically observed is driven by prolonged T cell survival and exposure to stimuli over an extended period of time. This is particularly relevant if loss of DJ-1 makes cells more refractory to stimulation.

Reply: We fully agree with the reviewer that the T-cell aging phenotype is very much driven by prolonged T cell survival and stimulation over an extended period of time. As we replied to the comments above, we now particularly stimulated purified naïve CD8 T cells with different doses of CD3 ab. Indeed, we observed that DJ-1 KO naïve CD8 T cells from young mice were more refractory to TCR stimulation. The compromised (but not fully lost) sensitivity to TCR stimulation in DJ-1 KO CD8 Tn over the long-lasting aging process might eventually contribute to the reduced immunoaging phenotypes (enhanced frequency of CD8 Tn but decreased fraction of CD8 Tem) we observed in this work.

In line with what the reviewer speculated, in fact, we only observed enhanced frequency of CD8 Tn of DJ-1-KO origin cells from aged BM within KO recipients, but not WT recipients.

This data clearly indicates that the non-hematopoietic cells (as part of the stimulation resource during the aging process) might play an important role in the observed reduced immunoaging phenotype. During the revision process, we have now performed TCR sensitivity experiments, which again indicates the critical role of prolonged T-cell activation in DJ-1-mediated immunoaging phenotypes.

Furthermore, as we already discussed in the text, ethically speaking, we cannot use aged mice as recipients (due to the harmful lethal irradiation procedure) and therefore cannot directly address whether the aging micro-environments regulates the immunoaging process in vivo. Since now we performed TCR sensitivity experiments, it can already at least address the related concern in vitro.

It is not clear to me why the mixed BM chimera results with respect to the Tn cells in particular were interpreted as either a haematopoietic-extrinsic or aging dependent phenomena. It is already clear from Figure 2b that the changes in Tn in DJ-1 KO mice are aging related. So given the mixed BM chimeras were young, one would not have expected a shift in relative percentages of WT and DJ-1 KO Tn cells. In light of this, I don't understand why the "haematopoietic-extrinsic" interpretation is being suggested.

Reply: We already replied to another relevant comment above. But we briefly discuss again here. Although the mixed BM chimera were from young donors, the mice already reached around 20 weeks after 8-week reconstitution of the immune system when being sacrificed and analyzed. Therefore, we expect at least partial immunoaging phenotypes and the BM chimera model could still partially reflect the immunoaging process. We now revised the text to make this point more clear (line 188-195, page 5-6).

The study makes an interesting observation regarding a level of maintenance of mitochondrial mass and membrane potential in aged DJ-1 KO mice that ostensibly aligns with the other markers of aging. However, this appears to be at odds with observations that mitochondrial mass increases in T cells with age (Quinn et al, Nat Commun, 2020). This should be discussed and relevant literature cited.

Reply: We now discussed this controversial point in our manuscript (line 304-311, page 9). It is difficult for us to fully understand the difference between different reports so far. Although Quinn et al observed increased mitochondrial mass in T cells, other reports might support our observations. For instance, Henson et al (*JCI,* 2014) have demonstrated reduced mitochondrial mass in more differentiated CD8 T cell subsets (CD8 TEMRA) relative to other subsets. Furthermore, recently, Desdin-Mico et al (*Science,* 2020) showed that depletion of

TFAM (mitochondrial transcription factor A) in T cells, causing reduced mtDNA and pre-matured senescence in T cells, indicative of an anti-aging role of mitochondria. Last but not least, a long time ago, it has already been documented that aging reduced skeletal muscle mitochondria (Crane et al, 2009, *Journal of Gerontology: Series A*).

In summary, the study clearly shows a phenotype of DJ-1 knockout, however it should investigate whether the amelioration of the ageing phenotype by DJ-1 KO is manifest functionally as well as phenotypically, should investigate the impact on TVM cells, and should discern whether it is secondary to an overall reduced sensitivity to stimulation by TCR and/or cytokines.

Reply: we fully agree with the reviewer that we need to investigate the aging phenotype of DJ-1 KO T cells functionally and should investigate the effect on TVM cells. We have now analyzed TVM frequency in both aged and young mice. In line with the notion of reduced immunoaging phenotype in DJ-1 KO mice, we indeed observed reduced frequency in CD8 TVM, from aged DJ-1 KO vs WT mice (added to Fig. 2i,j). But there was no difference in young DJ-1 KO and WT mice.

Furthermore, we also stimulated CD8 Tn isolated from aged mice with different doses of CD3 ab and showed an encouraging 'younger' phenotype at the functional levels, at least in terms of proliferation and activation (new Fig. 4 generated during revision). From young mice, DJ-1 KO vs WT CD8 Tn was indeed less sensitive to TCR stimulation (Fig 4). The compromised TCR sensitivity of DJ-1 KO CD8 Tn generated at a younger age would reduce activation and accumulate fewer overall homeostatic replication times of the DJ-1 KO CD8 Tn during the natural aging process. After reaching a critical age, those long-lived CD8 Tn (Vrisekoop et al, 2008) that were generated at a younger age would remain more proliferation potential (Fig. 4), i.e., relatively 'younger' also at the functional level. At the same time, the reduced TCR sensitivity of DJ-1 KO CD8 Tn from a younger age would eventually accumulate a lower frequency of CD8 Tem but a higher frequency of CD8 Tn after reaching a critical age, also indirectly leading to a "younger" immune phenotype. We have now added several paragraphs to describe and discuss those various results and OXPHOS and glycolysis results (new Fig 5, as suggested by another reviewer) in line 312-355, page 9-10.

Minor Points

The analysis of Ki67 staining in homeostatic conditions (Fig 2) doesn't address the question of T cell activation vs cell exhaustion, as stated. Homeostatic proliferation is not a reflection of

activation. This analysis investigates whether homeostatic proliferation might be responsible for elevated activation/exhaustion markers. This should be reworded.

The following sentence is a bit misleading, "Our recent work have demonstrated that DJ-1 depletion (Bonifati et al. 2003) reduced CD4 regulatory T cells (Treg) cellularity only in aged, but not young adult mice (Daniléviciute et al. 2019)." The recent work referred to is Daniléviciute et al and not Bonifati et al. Please amend.

Reply: Thanks for pointing out this mistake. In line 77, page 2, we now removed reference (Bonifati et al 2003).

Note that the Britanova et al, 2014 paper cited does not analyse TCR repertoire of naïve cells, but rather total cells from naïve individuals. Thus, the reference does not support the statement "During aging, the TCR repertoire diversity decreases in naïve T cells." and should be removed.

Reply: Thanks for pointing out this mistake. In line 101, page 3, we now removed this citation and instead inserted other citations (Qi et al , 2004 and Egorov et al., 2008, where the authors analyzed TCR repertoire in naïve T cells).

Recommend editing the title for Extended Data Fig 3 for grammar.

Reply: We now edited the title of Extended data Figure 3 (Now called Figure EV3) as suggested. The new title now read as "DJ-1 depletion also reduced signs of immunoaging in CD4 T-cell compartments from aged mice".

Y axis on extended data Fig 4f needs to be fixed.

Reply: We now fixed the Y axis in Supplementary Figure 4F (now called Figure EV4). We previously added the axis title twice with unknown reasons.

Referee #3:

In the submitted manuscript 'DJ-1 depletion slows down immunoaging in T-cell compartments', Zeng and colleagues report on the implication of loss of DJ-1 on the T lymphocyte compartment during aging in mouse and human. DJ-1 deficiency is correlated with a reduction in several signs of immunoaging related to T cells, with respect to prevalence of certain subpopulations to the expression of immunosenescence marker.

All in all, this is a great example of combining clinical findings with genetic model systems for further validation with significant interest for the general readers interested in aging. DJ-1 had not

been put into context of immunoaging. The manuscript is well-written and the results are convincingly reported. I recommend this manuscript to be published after revising some minor points:

Reply: Thanks for the overall appreciation on our work.

Minor comments

1. The authors need to make sure that it is clear in any case (esp. in the abstract, introduction and discussion/summary paragraph), that they refer to immunoaging in the T lymphocyte compartment, as the effect on myeloid cells has not been studied.

Reply: Thanks for stressing this point. Yes, to be precise, we did not mention anywhere the myeloid cells and we now checked the abstraction, main text (Introduction, Results and Discussion part) and even figure legends to make sure that we only referred to Immunoaging in T cells.

2. Please note that 'gender' refers to your own perceived sexual identity. The authors should replace gender in all incidences with 'sex'.

Reply: Thanks for pointing out this mistake. We now systematically replaced all "gender" with the word "sex".

3. The authors use the term 'delay' to describe the found phenotypes related to immunoaging. Can this be specified how long this is? Otherwise, I would suggest to write about the 'diminished signs of immunoaging'.

Reply: Thanks for mentioning this aspect. We cannot specify how long the phenotype was delayed. Therefore, we now rephrased everything to either "reduced immunoaging" or "diminished signs of immunoaging" or "diminished immunoaging phenotypes" or "diminished immunoaging features" as the reviewer suggested. This correction has been done even in title and abstract.

4. Please cite and discuss the literature on DJ-1 and T cells, e.g. PMID 24953490 reporting on increased IFNg levels in DJ-1 KO Th1 cells.

Reply: We now discussed this paper in the end, in line 373-376, page 11.

5. End of 3rd paragraph: 'No increase or decrease trend was observed in systematic levels of relevant pro-inflammatory cytokines...' This should probably read 'systemic' if I am not mistaken.

Reply: Thanks for your careful reading. Yes, we now revised it to “systemic”.

6. Page 4, last paragraph: '...we have also observed delayed immunoaging features in CD4 T cells of the index patent.' This should probably read 'patient'.

Reply: Yes, we now corrected the typo “patent” to “patient”.

Dear Dr. Hefeng,

Thank you for submitting your revised manuscript. It has now been seen by two of the original referees.

I apologize for the delay in getting back to you, it took longer than anticipated to receive the referee reports.

Referees acknowledge that the revision significantly improved the manuscript. However, referee #2 has significant remaining concerns. I have discussed these concerns with referee #1. Referee #1 partially agrees with these concerns, but following the discussion, we concluded that the concerns do not invalidate the main message of the manuscript. However, textual revisions are required for addressing these concerns.

In particular, referee #2 finds that 45 week old mice are not old enough to be considered as old mice (points 1 and 2). I have discussed this concern further with referee #1, who agrees with the concern. However, referee #1 finds that mice at this age already start showing the decline of naive T cells and the increase of memory cells. Therefore, please refer to these mice with their age (e.g. 45 week old mice), instead of calling them old mice, throughout the figures and the text. To address the 3rd concern of referee #2, please also present the absolute numbers of Tvm cells. As per the 4th concern of referee #2, referee #1 agrees that young DJ-1 knockout mice exhibit increased CD8 T cell sensitivity, but he/she also finds that this difference increases in old (middle-aged) animals, which suggests that there is an age-dependent effect, it is not a mere effect on sensitivity that is preserved. However, please make sure to discuss and acknowledge that the sensitivity difference observed in already in the young mice, which increases in an age-dependent manner. Please mark the changes in the text.

Furthermore, I need you to address the editorial points below before I can accept the manuscript.

• For technical reasons, we can only accommodate 5 keywords, and there are currently 6. Please remove one of the keywords.
• We note that Figures EV1 i-k are currently not called out in the text.
• There are callouts to Appendix Fig S1 which need correcting/deleting.
• Papers published in EMBO Reports include a 'synopsis' and 'bullet points' to further enhance discoverability. Both are displayed on the html version of the paper and are freely accessible to all readers. The synopsis includes a short standfirst summarizing the study in 1 or 2 sentences (max 35 words) that summarize the paper and are provided by the authors and streamlined by the handling editor. I would therefore ask you to include your synopsis blurb and 3-5 bullet points listing the key experimental findings.
• In addition, please provide an image for the synopsis. This image should provide a rapid overview of the question addressed in the study but still needs to be kept fairly modest since the image size cannot exceed 550x400 pixels.

Thank you again for giving us to consider your manuscript for EMBO Reports, I look forward to your minor revision.

Kind regards,

Deniz Senyilmaz Tiebe

--
Deniz Senyilmaz Tiebe, PhD
Scientific Editor
EMBO Reports

Referee #1:

In the previous review round, my main observations regarded the fact that most observations on the effects of DJ-1 on T cell aging were phenotypic, rather than functional, particularly as far as mitochondrial functionality is concerned. The authors have fully addressed my main concerns; the new data concerning immunometabolic properties of T cells in the DJ-1 mouse model during aging are properly presented and discussed.

I have no further comments or requests.

Referee #2:

The authors have done a number of the suggested experiments and provided explanations for existing data. I accept that there is a real consequence of loss of DJ-1 especially as it relates to sensitivity of the T cells to stimulation. However, I do not feel that all issues have been adequately addressed, as outlined below.
- Firstly, it has become clear to me that the 'aged' mice are only 45 weeks old. This is not the widely accepted age range of mice considered to reflect elderly humans. This link defines that ageing in mice is considered to occur from 18-24 months of age

(https://www.jax.org/news-and-insights/jax-blog/2017/november/when-are-mice-considered-old#). Certainly our own experience suggests that classical hallmarks of immune aging do not occur until at least 15 months of age.
- For this reason, I do not accept that mice that are 20 weeks of age could be considered to be partly aged and find this to be a complicated explanation for the BM chimera data.
- While the authors have quantitated the % of Tvm cells and shown that they do not increase, it is the markers of senescence that are of particular interest. In WT mice, the % of Tvm cells only increases with age because the Tn cells decrease; the # of Tvm cells remains stable. Generally, there are only measures of percentages stated here, rather than absolute numbers which would be more telling.
- The new data showing TCR responsiveness essentially shows reduced sensitivity in DJ-1 KO cells in younger mice and that same sensitivity is maintained with age. Thus, it seems clear that DJ-1 plays a role primarily in sensitivity to stimulation rather than directly in T cell aging per se. This explains a great deal of the data without needing to refer to an 'aging phenotype'. For example, it makes sense that markers of activation such as PD1, Eomes, LAG3 etc would be increased on WT cells that are more receptive to stimulation, that there would be a larger proportion of true naïve T cells, increased clonal diversity, reduced Ki67 etc. This is all relative. There is very little in the way of a definitive measure of 'senescence' or an 'aging' phenotype per se.

Minor points:-
- Why are CD27+CD28+ CD8 T cells defined as 'non-senescent'? They are the definition of senescence in CD4+ T cell populations, for example in many studies by Akbar et al.
- The flow cytometric staining generally looks poor for PD1, Eomes, CD57 and Tbet.

Referee #1:

In the previous review round, my main observations regarded the fact that most observations on the effects of DJ-1 on T cell aging were phenotypic, rather than functional, particularly as far as mitochondrial functionality is concerned. The authors have fully addressed my main concerns; the new data concerning immunometabolic properties of T cells in the DJ-1 mouse model during aging are properly presented and discussed.

I have no further comments or requests.
Reply: Thanks for appreciating our work and accepting our work.

Referee #2:

The authors have done a number of the suggested experiments and provided explanations for existing data. I accept that there is a real consequence of loss of DJ-1 especially as it relates to sensitivity of the T cells to stimulation. However, I do not feel that all issues have been adequately addressed, as outlined below.
- Firstly, it has become clear to me that the 'aged' mice are only 45 weeks old. This is not the widely accepted age range of mice considered to reflect elderly humans. This link defines that ageing in mice is considered to occur from 18-24 months of age (https://www.jax.org/news-and-insights/jax-blog/2017/november/when-are-mice-considered-old#). Certainly our own experience suggests that classical hallmarks of immune aging do not occur until at least 15 months of age.

Reply: Thanks for highlighting this aspect. To address the concern raised by the reviewer, we now systematically rephrased "aged mice" as "45-wk-old mice", or specified the age of the old mice if the used mice were much older than 45 weeks in all the main and Expanded View Figures and manuscript text. In some places, we also mentioned the term "middle-aged" for discussion. In fact, when checking the precise age information, we noticed that some of the experiments (e.g., the Tvm analysis) performed during the last revision were based on the mice slightly older than 60 weeks (around 14-month old). Due to the long-lasting nature of the project, we have almost no chances to strictly to only work with mice within a very narrow window of age. The effect of DJ-1 on the frequency of CD8 T-cell subsets in 60-wk-old mice has already been shown in main **Figure 2 (i-l)** even in the last revision. In the updated **Figure EV2 (c-g)** of the current revision, where we displayed the cell numbers of different CD8 subsets, the same effect of DJ-1 on immunoaging phenotypes has been kept. To facilitate the reviewing process, we directly copied the updated **Figure EV2** below.

Last but not least, as noticed by another reviewer as mentioned in the decision letter, the immunoaging hallmarks we observed here could already happen in 45-wk-old mice in their laboratories. The difference in the age starting to show immunoaging hallmarks might be attributable to hygiene degree in different animal

facilities (although all under SPF), as more and more evidence shows that gut microbiota might modulate the immune systems (http://www.hbausa.org/wp-content/uploads/2016/10/hbausa_elections_2016_candidates.pdf) and possibly the long-lasting cumulative effects of even mild difference in gut microbiota compositions eventually cause the difference in immunoaging speed and timing.

In short, our observations, although the majority of which were made in 45-wk-old mice, are still valid and well support our major statements.

- For this reason, I do not accept that mice that are 20 weeks of age could be considered to be partly aged and find this to be a complicated explanation for the BM chimera data.

Reply: Thanks for pointing out this aspect. We agree with the reviewer that this explanation is not ideal. We now removed the related sentences in the text. We believe that the lymphopenia-induced hyperactive reconstitution and the accelerated development of the immune system might be already a sufficient and plausible explanation for the BM chimera data.

- While the authors have quantitated the % of Tvm cells and shown that they do not increase, it is the markers of senescence that are of particular interest. In WT mice, the % of Tvm cells only increases with age because the Tn cells decrease; the # of Tvm cells remains stable. Generally, there are only measures of percentages stated here, rather than absolute numbers which would be more telling.

Reply: Thanks for suggesting this. We now checked the original datasets and calculated the absolute number of CD8 Tn, Tvm and Tmem cells within the same amount (2e4) of acquired living CD3 T cells from young or 60-wk-old mice. We also directly copied the updated **Figure EV 2** in the response letter below. Clearly, even in terms of absolute number, DJ-1 depletion also reduced T-cell immunoaging signs in 60-wk-old mice. With our experimental setting, we were unable to precisely estimate the cell number of CD8 Tvm in total splenocytes. Different from many others, we stopped the flow cytometry acquisition, when the same number (20K) of living CD3 T cells was achieved, to make sure that the flow cytometry plots are really comparable. In any case, the absolute numbers of CD8 subsets within the same amount of living CD3 T cells are already informative enough. Considering there were no significant changes in both CD3 frequency and total splenocytes between 60-wk-old Dj-1 KO and WT mice, the estimated absolute numbers of CD8 subsets in total splenocytes should retain a similar pattern as the absolute numbers of CD8 subsets within the same amount of total CD3 T cells. We have also now described the related results in line 161-174, page 5.

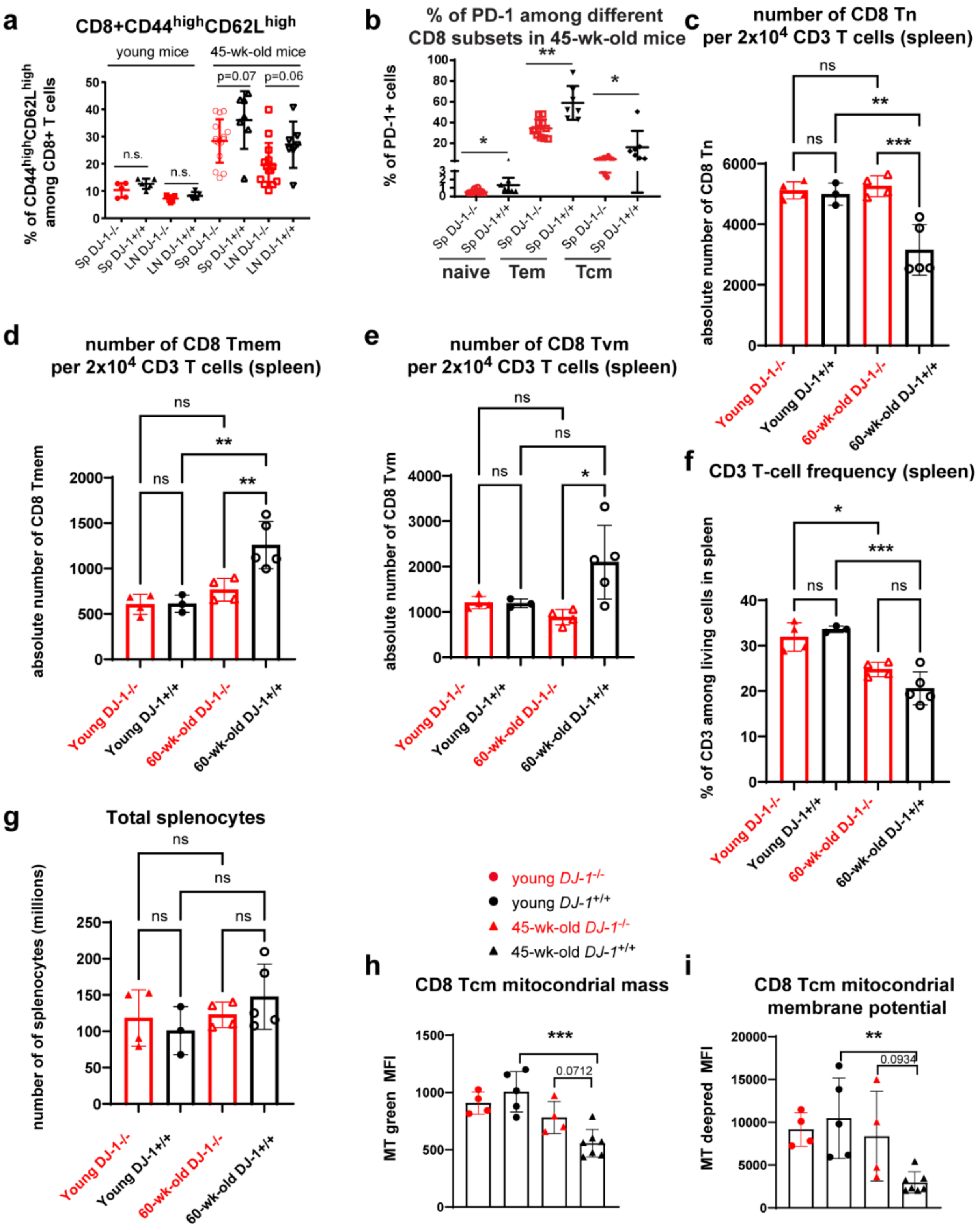

Updated Figure EV 2.

- The new data showing TCR responsiveness essentially shows reduced sensitivity in DJ-1 KO cells in younger mice and that same sensitivity is maintained with age. Thus, it seems clear that DJ-1 plays a role primarily in sensitivity to stimulation rather than directly in T cell aging per se.

This explains a great deal of the data without needing to refer to an 'aging phenotype'. For example, it makes sense that markers of activation such as PD1, Eomes, LAG3 etc would be increased on WT cells that are more receptive to stimulation, that there would be a larger proportion of true naïve T cells, increased clonal diversity, reduced Ki67 etc. This is all relative. There is very little in the way of a definitive measure of 'senescence' or an 'aging' phenotype per se.

Reply: We agree with the reviewer that the new TCR sensitivity data essentially showed that the TCR sensitivity of DJ-1 KO CD8 Tn was quite preserved between the young and older mice. We also agree that the TCR sensitivity was compromised already at a younger age. Nevertheless, the preserved TCR sensitivity alone cannot fully explain why we only observed all the immune cellular phenotypes in older (at least 45-wk-old) mice, but not in younger mice. In fact, as shown in **Figure 4**, the difference in TCR sensitivity between DJ-1 KO and WT mice that already started from a young age increased in an aging-dependent manner. In another words, during natural aging, TCR sensitivity of CD8 Tn decreased dramatically in WT mice, while that in DJ-1 KO CD8 Tn was declined not as dramatically as in WT mice. Of note, to more logically discuss the aspects raised by the reviewer, we now swapped the position of subpanels of TCR sensitivity data between young and older mice in **Figure 4.**

Furthermore, if we consider **Figure 2b, c** and many other cellular data presented in this work, a similar observation reoccurred throughout the manuscript. That is, the immunoaging markers showed a dramatic increase (or decrease) in WT mice, but that showed a much milder change in DJ-1 KO mice during the aging process. The differences in the changing slopes over time of various immunoaging markers between DJ-1 KO and WT mice together contributed to a relatively reduced immunoaging phenotype in DJ-1 KO mice. Importantly, we would like to stress again, almost no cellular phenotypes (e.g., frequency of naïve and memory T cells etc.) appeared in young mice, clearly indicating an aging-dependent phenotype.

We have also now discussed the better preserved TCR sensitivity in DJ-1 KO mice and also the fact that the difference in TCR sensitivity between DJ-1 KO and WT mice already starting from a young age increases in an aging-dependent manner in line 303-307, page 9 and line 395-402, page 11.

Minor points:-
- Why are CD27+CD28+ CD8 T cells defined as 'non-senescent'? They are the definition of senescence in CD4+ T cell populations, for example in many studies by Akbar et al.

Reply: Thanks for noticing this potential aspect. We just checked the papers from Akbar and colleagues again. We are sure that CD27+CD28+ CD8 T cells are non-senescent T cells. For instance, in the recent paper by

Pereira et al (https://www.nature.com/articles/s41590-020-0643-3), the authors wrote "*Expression of CD28 and CD27 is sequentially lost on CD8⁺ T cells as they transition from* ***CD27⁺CD28⁺ CD8⁺ T_N cells*** *to CD27⁺CD28⁻ intermediate CD8⁺ T cells and to terminal, or senescent-like, CD27⁻CD28⁻ CD8⁺ T cells.* ". In one of the previous papers also from Akbar's group (https://www.nature.com/articles/ni.3665), where they focused on CD4 T cells, the authors wrote "***Early-stage T cells within the CD4 compartment are CD27⁺CD28⁺****, those at an intermediate stage are CD27⁻CD28⁺, and senescent T cells are CD27⁻CD28⁻*". Therefore, we are confident that CD27+CD28+ CD8 T cells we referred here should be called "non-senescent" T cells and might be even called CD8 Tn if we are not conservative enough.

- The flow cytometric staining generally looks poor for PD1, Eomes, CD57 and Tbet.

Reply: We agree with the reviewer that the staining for PD-1, Eomes, CD57 and T-bet in human samples looks not so great. While we cannot change the staining quality anymore, we tried to improve the visualization of those panels **of Figure 1** by changing the axis scales of flow cytometry plots (e.g., changing from 10^5 to 10^4 to zoom in the populations in some cases). But we cannot get better ones for CD57. In the current revision, we also replaced the previous CD27 VS CD28 plots with better ones. In any case, even with the previous version, they all can deliver a clear and consistent message that the PD index patient (P2) had a much smaller fraction of cells expressing those markers than the two siblings. In combination with all the other results, we are confident that the flow cytometric data about those markers are reliable. To simplify the reviewing procedure, we also copied the updated part of **Figure 1** below.

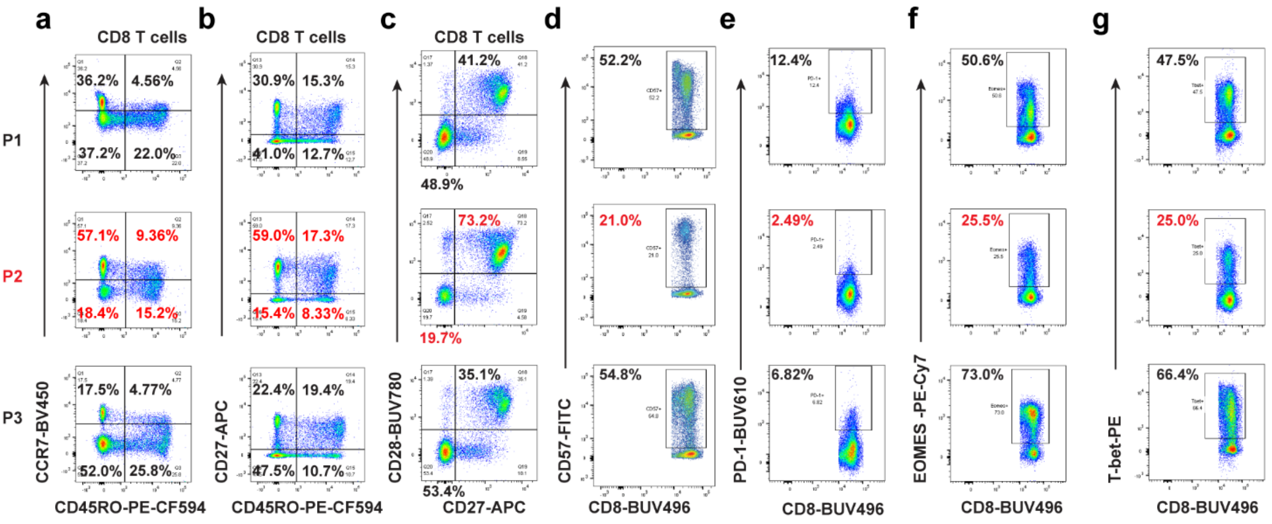

Dear Feng,

Thank you for submitting your revised manuscript. I have now looked at everything and all is fine. Therefore, I am very pleased to accept your manuscript for publication in EMBO Reports.

Congratulations on a nice work!

Kind regards,

Deniz
--
Deniz Senyilmaz Tiebe, PhD
Editor
EMBO Reports

--
At the end of this email I include important information about how to proceed. Please ensure that you take the time to read the information and complete and return the necessary forms to allow us to publish your manuscript as quickly as possible.

As part of the EMBO publication's Transparent Editorial Process, EMBO reports publishes online a Review Process File to accompany accepted manuscripts. As you are aware, this File will be published in conjunction with your paper and will include the referee reports, your point-by-point response and all pertinent correspondence relating to the manuscript.

If you do NOT want this File to be published, please inform the editorial office within 2 days, if you have not done so already, otherwise the File will be published by default [contact: emboreports@embo.org]. If you do opt out, the Review Process File link will point to the following statement: "No Review Process File is available with this article, as the authors have chosen not to make the review process public in this case." Please note that the author checklist will still be published even if you opt out of the transparent process.

Thank you again for your contribution to EMBO reports and congratulations on a successful publication. Please consider us again in the future for your most exciting work.

****

THINGS TO DO NOW:

You will receive proofs by e-mail approximately 2-3 weeks after all relevant files have been sent to our Production Office; you should return your corrections within 2 days of receiving the proofs.

Please inform us if there is likely to be any difficulty in reaching you at the above address at that time. Failure to meet our deadlines may result in a delay of publication, or publication without your corrections.

All further communications concerning your paper should quote reference number EMBOR-2021-53302V3 and be addressed to emboreports@wiley.com.

Should you be planning a Press Release on your article, please get in contact with emboreports@wiley.com as early as possible, in order to coordinate publication and release dates.

EMBO PRESS

YOU MUST COMPLETE ALL CELLS WITH A PINK BACKGROUND ➔

PLEASE NOTE THAT THIS CHECKLIST WILL BE PUBLISHED ALONGSIDE YOUR PAPER

Corresponding Author Name: Feng Q. Hefeng

Journal Submitted to: EMBO Reports

Manuscript Number: EMBOR-2021-53302V1

Reporting Checklist For Life Sciences Articles (Rev. June 2017)

This checklist is used to ensure good reporting standards and to improve the reproducibility of published results. These guidelines are consistent with the Principles and Guidelines for Reporting Preclinical Research issued by the NIH in 2014. Please follow the journal’s authorship guidelines in preparing your manuscript.

A- Figures

1. Data

The data shown in figures should satisfy the following conditions:

- ➔ the data were obtained and processed according to the field’s best practice and are presented to reflect the results of the experiments in an accurate and unbiased manner.
- ➔ figure panels include only data points, measurements or observations that can be compared to each other in a scientifically meaningful way.
- ➔ graphs include clearly labeled error bars for independent experiments and sample sizes. Unless justified, error bars should not be shown for technical replicates.
- ➔ if n< 5, the individual data points from each experiment should be plotted and any statistical test employed should be justified
- ➔ Source Data should be included to report the data underlying graphs. Please follow the guidelines set out in the author ship guidelines on Data Presentation.

2. Captions

Each figure caption should contain the following information, for each panel where they are relevant:

- ➔ a specification of the experimental system investigated (eg cell line, species name).
- ➔ the assay(s) and method(s) used to carry out the reported observations and measurements
- ➔ an explicit mention of the biological and chemical entity(ies) that are being measured.
- ➔ an explicit mention of the biological and chemical entity(ies) that are altered/varied/perturbed in a controlled manner.

- ➔ the exact sample size (n) for each experimental group/condition, given as a number, not a range;
- ➔ a description of the sample collection allowing the reader to understand whether the samples represent technical or biological replicates (including how many animals, litters, cultures, etc.).
- ➔ a statement of how many times the experiment shown was independently replicated in the laboratory.
- ➔ definitions of statistical methods and measures:
  - common tests, such as t-test (please specify whether paired vs. unpaired), simple χ2 tests, Wilcoxon and Mann-Whitney tests, can be unambiguously identified by name only, but more complex techniques should be described in the methods section;
  - are tests one-sided or two-sided?
  - are there adjustments for multiple comparisons?
  - exact statistical test results, e.g., P values = x but not P values < x;
  - definition of ‘center values’ as median or average;
  - definition of error bars as s.d. or s.e.m.

Any descriptions too long for the figure legend should be included in the methods section and/or with the source data.

In the pink boxes below, please ensure that the answers to the following questions are reported in the manuscript itself. Every question should be answered. If the question is not relevant to your research, please write NA (non applicable). We encourage you to include a specific subsection in the methods section for statistics, reagents, animal models and human subjects.

USEFUL LINKS FOR COMPLETING THIS FORM

http://www.antibodypedia.com	Antibodypedia
http://1degreebio.org	1DegreeBio
http://www.equator-network.org/reporting-guidelines/improving-bioscience-research-repor	ARRIVE Guidelines
http://grants.nih.gov/grants/olaw/olaw.htm	NIH Guidelines in animal use
http://www.mrc.ac.uk/Ourresearch/Ethicsresearchguidance/Useofanimals/index.htm	MRC Guidelines on animal use
http://ClinicalTrials.gov	Clinical Trial registration
http://www.consort-statement.org	CONSORT Flow Diagram
http://www.consort-statement.org/checklists/view/32-consort/66-title	CONSORT Check List
http://www.equator-network.org/reporting-guidelines/reporting-recommendations-for-tumo	REMARK Reporting Guidelines (marker prognostic studies)
http://datadryad.org	Dryad
http://figshare.com	Figshare
http://www.ncbi.nlm.nih.gov/gap	dbGAP
http://www.ebi.ac.uk/ega	EGA
http://biomodels.net/	Biomodels Database
http://biomodels.net/miriam/	MIRIAM Guidelines
http://jjj.biochem.sun.ac.za	JWS Online
https://osp.od.nih.gov/biosafety-biosecurity-and-emerging-biotechnology/	Biosecurity Documents from NIH
http://www.selectagents.gov/	List of Select Agents

B- Statistics and general methods

Please fill out these boxes ➔ (Do not worry if you cannot see all your text once you press return)

1.a. How was the sample size chosen to ensure adequate power to detect a pre-specified effect size?	For each subfigure related to mice, in most cases, at least five mice were chosen per genotype group at the given age and have been repeated at least three times in different cohorts. For the TVM and CD31 analysis, we chose at least three mice per genotype group at the given age.
1.b. For animal studies, include a statement about sample size estimate even if no statistical methods were used.	We chose at least 5 mice per genotype and age group (the same sex) for each analysis and each analysis has been repeated at least three times.
2. Describe inclusion/exclusion criteria if samples or animals were excluded from the analysis. Were the criteria pre-established?	NA.
3. Were any steps taken to minimize the effects of subjective bias when allocating animals/samples to treatment (e.g. randomization procedure)? If yes, please describe.	For the immunoaging phenotype analysis in mice, the WT or KO littermates were always maintained in the same cages. For the bone marrow transplanation experiments, the CD45.1 and CD45.2 mixture were transferred into the same recipients and therefore we tried our best to minimize the potential bias in our projects.
For animal studies, include a statement about randomization even if no randomization was used.	added in the Materials and Methods section under the subheading "Animals"
4.a. Were any steps taken to minimize the effects of subjective bias during group allocation or/and when assessing results (e.g. blinding of the investigator)? If yes please describe.	The operators were blind to the genetic information of the three human participants before the analysis. The operators were also blind to the genetic group of mice before finishing the flow cytometry analysis.
4.b. For animal studies, include a statement about blinding even if no blinding was done	added in the Materials and Methods section under the subheading "Animals"
5. For every figure, are statistical tests justified as appropriate?	Yes
Do the data meet the assumptions of the tests (e.g., normal distribution)? Describe any methods used to assess it.	Yes, we applied the Shapiro-Wilk test.
Is there an estimate of variation within each group of data?	Yes
Is the variance similar between the groups that are being statistically compared?	Yes

C- Reagents

6. To show that antibodies were profiled for use in the system under study (assay and species), provide a citation, catalog number and/or clone number, supplementary information or reference to an antibody validation profile. e.g., Antibodypedia (see link list at top right), 1DegreeBio (see link list at top right).	All the detailed information of the used antibodies and the key reagents have been provided in the Appendix. We added here again. We only used antibodies commercially available, which have been routinely validated by the providers and often by other literatures. Table S1. List of mouse-related antibodies used in this work.<br>Antibody Clone Company Catalogue number Dilution factor and application*<br>CD16/CD32 2.4G2 BD Biosciences 553141 1:50<br>CD3-BV421 145-2C11<br>BD Biosciences 562600<br>1:200<br>CD3-APC 17A2 Biolegend 100236 1:200 (sorting)<br>CD25-APC PC61<br>BD Biosciences 557192<br>1:100 (sorting)<br>CD25-PE-Cy7 PC61.5 eBioscience 25-0251-82 1:200<br>CD25-BUV395 PC61 BD Biosciences 564022 1:100(mitotracker)<br>CD44-PE-Cy7 IM7 BD Biosciences 560569 1:200<br>(sorting)<br>CD4-BUV496 GK1.5 BD Biosciences 564667 1:200<br>CD4-FITC RM4-5
	eBioscience 11-0042-82<br>1:100 (sorting)<br>CD4-PE RM4-5<br>Biolegend 100512 1:200 (mitotracker)<br>CD62L-PerCP-Cy5.5 MEL-14 BD Biosciences 560513<br>1:200<br>(sorting)<br>CD8-BUV805 53-6.7 BD Biosciences 564920<br>1:200<br>CD8-BV737 53-6.7 BD Biosciences 612759 1:200(mitotracker)<br>CD8-eFluor450 53-6.7 eBioscience 48-0081-82 1:100 (sorting)<br>CTLA4-PE UC10-4B9 eBioscience 12-1522-83 1:100<br>IFNγ-BV711 XMG1.2 BD Biosciences 564336 1:100<br>Ki-67-BV605 16A8 BioLegend 652413 1:200<br>Ki-67-EF450 SolA15 eBioscience 48-5698-82 1:100<br>PD-1-BV711 J43 BD Biosciences 744547 1:100<br>KLRG1-PERCP-CY5.5 2F1/KLRG1 Biolegend 138417<br>1:100<br>KLRG1-BV785 2F1/KLRG1 Biolegend 138429 1:100<br>CD45.1-BV785 A20 Biolegend 110743 1:200<br>CD45.2-BV650 104 Biolegend 109835 1:200 *, if not specified, the application is conventional FACS analysis;

	Table S2. List of other materials used in mouse-related experiments of the study.<br>Materials Company Cat. Number<br>Agilent RNA 6000 Nano kit Agilent 5067-1511<br>Agilent RNA 6000 Pico kit Agilent 5067-1513<br>CD90.2 MicroBeads, mouse Miltenyi Biotec 130-049-101<br>RPMI 1640 Lonza 12-167F<br>eBioscience™ Foxp3 / Transcription Factor Staining Buffer Set eBioscience 00-5523-00<br>Fetal bovine serum (FBS) Gibco 10270106<br>Fixation/Permeabilization Solution Kit BD Biosciences 554714<br>Golgiplug BD Biosciences 555029<br>Golgistop BD Biosciences 554724<br>Ionomycin Sigma-Aldrich I0634<br>Glutamax (100X) Thermo Fisher Scientific 35050061<br>Sodium Pyruvate Thermo Fisher Scientific 11360070<br>HEPES Thermo Fisher Scientific 15630080<br>PBS Lonza BE17-516F<br>PMA Sigma-Aldrich P8139<br>Red blood cell lysis buffer BD 555899<br><br>RNeasy Mini Spin Kit Qiagen 74104<br>DNAse I Qiagen 79254<br>MEM non-essential amino acids Sigma-Aldrich M7145<br>0.5 M EDTA Sigma-Aldrich E7889<br>Penicillin+Streptomycin Thermo Fisher Scientific 15070-063<br>Beta-Mercaptoethanol Sigma-Aldrich M7522
	Table S3. Materials or reagents used for human PBMC isolation and flow cytometry analysis.<br>Name Company Ref. number<br>SepMate tubes StemCell 86450<br>Lymphoprep StemCell 07801<br>True-Nuclear Transcription Factor Buffer Set BioLegend 424401<br>Brilliant Stain Buffer BD 563794<br>UltraComp eBeads eBioscience 01-2222-42<br><br>Table S4. List of antibodies used for sorting or analysing human T cells.<br>Protein Color Dilution Company Ref. number Clone<br>Analysis staining abs<br>Fc Blocking Abs / 1:50 BD 564765 /<br>CD4 BUV395 1:40 BD 563550 SK3 aka Leu3a<br>CD8 BUV496 1:40 BD 564804 RPA-T8<br>CD27 APC 1:40 BD 561786 M-T271<br>CD28 BUV785 1:40 BioLegend 302950 CD28.2<br>CD45RO PE-CF594 1:40 BD 562299 UCHL1<br>CD57 FITC 1:40 BD 555619 NK-1<br>CD197 (CCR7) Pacific Blue 1:40 BioLegend 353210 G043H7<br>Eomes PE-Cy7 1:20 Thermo Fischer Scientific 25-4877-42 WD1928<br>FOXP3 APC 1:20 BioLegend 320114 206D<br>Ki-67 FITC 1:20 BD 561165 B56<br>PD-1 BV605 1:40 BioLegend 329924 EH12.2H7<br>T-bet PE 1:20 BioLegend 644810 4B10<br>Live/Dead APC-Cy7 1:500 Thermo Fischer Scientific L34976 /
	Microarray analysis sorting panel<br>CD4 FITC 1:20 BD 555346 RPA-T4<br>CD8 BV605 1:20 BioLegend 301040 RPA-T8<br>Live/Dead APC-Cy7 1:500 Thermo Fischer Scientific L34976 /<br>TCR repertoire analysis sorting panel<br>Fc Blocking Abs / BD Bioscience / 564765 1:50<br>CD3 HIT3a BD Bioscience BV510 741822 1:100<br>CD4 RPA-T4 BD Bioscience FITC 555346 1:100<br>CD8 RPA-T8 Biolegend BV605 301040 1:100<br>CD45RA HI100 Biolegend Pacific Blue 304123 1:50<br>CD45RO UCHL1 BD Bioscience PE-CF594 562299 1:50
7. Identify the source of cell lines and report if they were recently authenticated (e.g., by STR profiling) and tested for mycoplasma contamination.	Only in vivo or ex vivo analysis has been performed. So the question is not relevant to our study.

* for all hyperlinks, please see the table at the top right of the document

D- Animal Models

8. Report species, strain, gender, age of animals and genetic modification status where applicable. Please detail housing and husbandry conditions and the source of animals.	B6.129P2-Park7Gt (XE726) Byg/Mmucd mice were previously described1. The DJ-1 knockout mice line used in our lab has been generated by crossing the original line with B6N mice at least 10 generations. Rag1-/- mice with C57BL/6 (B6) background were purchased from the Jackson Laboratory and maintained in the specific pathogen free (SPF) animal facility of Luxembourg Institute of Health. CD45.1 mice with B6 background were purchased from Charles River Laboratory and maintained in the SPF animal facility of University of Luxembourg. The DJ-1-/- (KO), DJ-1+/-, DJ-1+/+ (WT) mice used in our experiments were gender- and age-matched siblings generated from heterozygous DJ-1+/- breeding pairs or trios. The DJ-1 line including both young and aged cohorts was also kept under the SPF conditions.
9. For experiments involving live vertebrates, include a statement of compliance with ethical regulations and identify the committee(s) approving the experiments.	Animal protocols have been approved either by animal welfare structure from LIH or animal experimentation ethics committee from University of Luxembourg, depending on where the experiments were performed. After institutional checking, each of the protocols was officially authorized by Luxembourg Ministry of Agriculture before starting the animal experiments.
10. We recommend consulting the ARRIVE guidelines (see link list at top right) (PLoS Biol. 8(6), e1000412, 2010) to ensure that other relevant aspects of animal studies are adequately reported. See author guidelines, under 'Reporting Guidelines'. See also: NIH (see link list at top right) and MRC (see link list at top right) recommendations. Please confirm compliance.	NA.

E- Human Subjects

11. Identify the committee(s) approving the study protocol.	We complied with all the relevant ethic regulations and Luxembourg CNER (Comité National d'Ethique de Recherche) has approved the PD patients related study.
12. Include a statement confirming that informed consent was obtained from all subjects and that the experiments conformed to the principles set out in the WMA Declaration of Helsinki and the Department of Health and Human Services Belmont Report.	The family carrying the c.192G>C mutation in the DJ-1 gene has been already described elsewhere and written informed consent for all the three participating individuals was obtained.
13. For publication of patient photos, include a statement confirming that consent to publish was obtained.	NA.
14. Report any restrictions on the availability (and/or on the use) of human data or samples.	NA.
15. Report the clinical trial registration number (at ClinicalTrials.gov or equivalent), where applicable.	NA.
16. For phase II and III randomized controlled trials, please refer to the CONSORT flow diagram (see link list at top right) and submit the CONSORT checklist (see link list at top right) with your submission. See author guidelines, under 'Reporting Guidelines'. Please confirm you have submitted this list.	NA.
17. For tumor marker prognostic studies, we recommend that you follow the REMARK reporting guidelines (see link list at top right). See author guidelines, under 'Reporting Guidelines'. Please confirm you have followed these guidelines.	NA.

F- Data Accessibility

18: Provide a "Data Availability" section at the end of the Materials & Methods, listing the accession codes for data generated in this study and deposited in a public database (e.g. RNA-Seq data: Gene Expression Omnibus GSE39462, Proteomics data: PRIDE PXD000208 etc.) Please refer to our author guidelines for 'Data Deposition'.<br><br>Data deposition in a public repository is mandatory for:<br>a. Protein, DNA and RNA sequences<br>b. Macromolecular structures<br>c. Crystallographic data for small molecules<br>d. Functional genomics data<br>e. Proteomics and molecular interactions	Yes, we provided the section "Data Availability" under the Materials and Methods. The microarray data from human CD8 T cells of the DJ-1 mutation carrier family and aged murine CD4 Tcon cells are deposited in Gene expression Omnibus (GEO) repository with the access code GSE173903 and GSE173904, respectively. To review the human CD8 dataset, go to https://www.ncbi.nlm.nih.gov/geo/query/acc.cgi?acc=GSE173903 and enter the token avgryqwodlqnbqt into the box. To review the mice dataset, go to https://www.ncbi.nlm.nih.gov/geo/query/acc.cgi?acc=GSE173904 and enter the token utirsmiaxzyvxkn into the box. Upon acceptance, we will make all those data including single-cell TCR repertoire sequencing data publically available from Adapative Biotechnologies (if we submitted the sequencing data to share now, the sequencing data will be automatically public since there is no a token control).
19. Deposition is strongly recommended for any datasets that are central and integral to the study; please consider the journal's data policy. If no structured public repository exists for a given data type, we encourage the provision of datasets in the manuscript as a Supplementary Document (see author guidelines under 'Expanded View' or in unstructured repositories such as Dryad (see link list at top right) or Figshare (see link list at top right).	Yes, we have already deposited all the large-scale datasets. We also now provided the numeric source data used to generate essential CD8 immunoaging phenotypic subfigures in Fig 2.
20. Access to human clinical and genomic datasets should be provided with as few restrictions as possible while respecting ethical obligations to the patients and relevant medical and legal issues. If practically possible and compatible with the individual consent agreement used in the study, such data should be deposited in one of the major public access-controlled repositories such as dbGAP (see link list at top right) or EGA (see link list at top right).	NA.
21. Computational models that are central and integral to a study should be shared without restrictions and provided in a machine-readable form. The relevant accession numbers or links should be provided. When possible, standardized format (SBML, CellML) should be used instead of scripts (e.g. MATLAB). Authors are strongly encouraged to follow the MIRIAM guidelines (see link list at top right) and deposit their model in a public database such as Biomodels (see link list at top right) or JWS Online (see link list at top right). If computer source code is provided with the paper, it should be deposited in a public repository or included in supplementary information.	NA.

G- Dual use research of concern

22. Could your study fall under dual use research restrictions? Please check biosecurity documents (see link list at top right) and list of select agents and toxins (APHIS/CDC) (see link list at top right). According to our biosecurity guidelines, provide a statement only if it could.	NA.
